# Antipsychotic Drugs and the Risk of Diabetic Complications: A Systematic Review of Clinical Evidence

**DOI:** 10.3390/jcm15083107

**Published:** 2026-04-18

**Authors:** Nisrine Haddad, Nawal Farhat, Christopher A. Gravel, Yue Chen, Franco Momoli, Donald R. Mattison, Jeannette Goguen, Daniel Krewski

**Affiliations:** 1 School of Epidemiology and Public Health, University of Ottawa, Ottawa, ON K1H 8M5, Canada; 2 School of Mathematics and Statistics, Carleton University, Ottawa, ON K1S 5B6, Canada; 3 Department of Mathematics and Statistics, University of Ottawa, Ottawa, ON K1N 6N5, Canada; 4 Data Literacy Research Institute, University of Ottawa, Ottawa, ON K1N 6N5, Canada; 5 Risk Sciences International, Ottawa, ON K1Z 7T1, Canada; 6 Arnold School of Public Health, University of South Carolina, Columbia, SC 29208, USA; 7 Department of Medicine, University of Toronto, Toronto, ON M5S 3H2, Canada; 8 Division of Endocrinology, St. Michael’s Hospital, Toronto, ON M5C 2T2, Canada

**Keywords:** antipsychotic drugs, diabetic ketoacidosis, hyperglycemic hyperosmolar state, case reports

## Abstract

**Background/Objective:** In recent years, epidemiological and clinical evidence has suggested an association between the use of second-generation antipsychotics (SGAs) and hyperglycemic complications: notably, diabetic ketoacidosis (DKA) and hyperglycemic hyperosmolar state (HHS). However, the role of first-generation antipsychotics (FGAs) remains less well understood. To conduct a systematic review of evidence established in case reports (CRs) on adverse drug reactions, specifically DKA and HHS, associated with the use of both FGAs and SGAs in order to identify patterns that may inform clinical awareness and future research. **Methods:** Pertinent bibliographic databases (MEDLINE, EMBASE, PsycINFO and the Cochrane Central Register of Controlled Trials (CENTRAL)) were searched using index phrases and keywords up until 17 October 2025. Eligible CRs discussed exposure to at least one US FDA-approved antipsychotic drug (APD) and assessed either DKA or HHS. **Results:** A total of 151 CRs were included in the systematic review (DKA, *n* = 121; HHS, *n* = 28; both conditions, *n* = 2). Patients aged 30 to 39 years accounted for the highest number of emergencies (*n* = 49, 32.5%), which occurred mostly in males (*n* = 108, 71.5%). The most common mental health diagnosis was schizophrenia (*n* = 77, 51%), followed by bipolar disorder (*n* = 26, 17.2%). Olanzapine was associated with the highest number of DKA cases (*n* = 53, 43.1%), followed by clozapine (*n* = 24, 19.5%). The average blood glucose at presentation was 842.8 mg/dL for DKA patients and 1252.8 mg/dL for HHS patients. The average hemoglobin A1c levels (HbA1c) were 11.5% and 12%, respectively, for these two conditions. Of the 12 reported fatalities, treatment with olanzapine was noted in four DKA cases and in one HHS case. **Conclusions:** This analysis provides additional evidence of an association between the use of atypical APDs and DKA or HHS. Clinicians should continue to monitor metabolic risk factors for these conditions, as well as educating patients about the prevention of acute diabetic complications.

## 1. Introduction

### 1.1. Antipsychotic Drugs

Antipsychotic drugs (APDs) are grouped into two classes—first-generation antipsychotics (FGAs), also known as typical APDs, and second-generation antipsychotics (SGAs), or atypical APDs—based on their mechanisms of action. While some of the recent literature has proposed a third generation of antipsychotics characterized by partial dopamine D2 receptor agonism [1,2], the two-class framework remains the most widely recognized and traditionally used in clinical and pharmacological contexts. Atypical APDs generally cause fewer extrapyramidal side effects, such as dystonia, dyskinesia, akathisia and parkinsonism [3,4]. Atypical APDs are currently more commonly prescribed to treat serious mental illnesses, including schizophrenia, bipolar disorder, and other psychotic disorders [4]. While they can improve cognitive and functional outcomes in treatment-compliant patients [5,6], this class of drugs may result in the development of metabolic syndrome (MetS), which is characterized by weight gain, dyslipidemia, fasting hyperglycemia and increased blood pressure, which can lead to poor adherence to treatment in those suffering from schizophrenia and other psychotic disorders [4]. Metabolic syndrome is defined by the presence of central obesity, along with at least two of the following symptoms, according to the criteria established by the International Diabetes Federation: elevated triglycerides, reduced HDL cholesterol, elevated blood pressure, or elevated fasting plasma glucose [7].

### 1.2. Hyperglycemic Complications

Evidence of an increased risk of hyperglycemic emergency complications—notably, diabetic ketoacidosis (DKA) and hyperglycemic hyperosmolar state (HHS)—associated with treatment with atypical APDs has accrued in recent years. To date, this association has not been widely reported with typical antipsychotics. DKA and HHS share a number of common risk factors, including newly diagnosed type 1 diabetes mellitus (T1DM) or type 2 diabetes mellitus (T2DM), inconsistent insulin treatment, inadequate insulin therapy in hospital settings and infections (such as skin and urinary tract infections). Other triggers include acute coronary/vascular events; trauma; treatment with glucocorticoids, beta-blockers, thiazide diuretics, certain atypical APDs [8,9,10,11,12] and some chemotherapeutic agents [11]; and other precipitating factors [9,13,14,15]. Both conditions are characterized by insulinopenia and severe hyperglycemia. Newly diagnosed DM (present in 17.2–23.8% of cases), infection (14.0–16.0%), poor treatment adherence (41.0–59.6%), other causes (9.7–18.0%), and unidentified causes (3.0–4.2%) are some of the triggering factors for DKA in the US [16]. Patients with a history of DM are more likely to report HHS. The primary triggering factors include infection (30–60%), omission of insulin or other antidiabetic medications, and comorbidities such as myocardial infarction [11,16].

Around 30% of patients with DKA have features of both DKA and HHS [17,18]. Clinically, DKA is characterized by hyperglycemia, hyperketonemia and metabolic acidosis, and can be classified from mild to severe with plasma glucose levels in excess of 250 mg/dL. HSS is characterized by plasma glucose higher than 600 mg/dL and serum osmolarity over 320 mmol/kg [19,20]. The latter condition, which is less common, occurs without ketoacidosis and ketonemia [21]. Table 1 summarizes the diagnostic criteria for these two relatively rare conditions.

### 1.3. Hospitalization Rates and Epidemiology for Diabetic Ketoacidosis and Hyperglycemic Hyperosmolar State

Despite well-defined diagnostic criteria and treatment regimens, DKA and HHS are significant contributors to morbidity and mortality in diabetes patients [21]. The incidence of DKA declined slightly between 2000 and 2009, and then increased, accounting for approximately 141,704 hospitalizations in 2009 and 188,950 in 2014 [22]. Similar trends were also observed in other countries such as England [23] and Finland [24]. While all age groups experienced an increase in DKA hospitalization rates of ≥6.0% annually, the increase was more pronounced among those who were less than 45 years of age. In contrast, in-hospital case fatalities declined across all age groups in both males and females during the same period [22]. DKA is more common in patients with T1DM; however, it can also occur in those with T2DM. This is supported by findings from the Swedish population, where DKA was observed in Caucasian T2DM patients, despite its higher prevalence in T1DM [11,25]. The incidence of DKA ranges from 0.32 to 2.0 per 1000 patient-years in patients with T2DM [25], and from 4.6 to 8.0 per 1000 patient-years in those with T1DM [20]. In Germany, the mean admission rate for DKA in T1DM patients was 4.8 per 100 patient-years (95% CI, 4.5–5.1) [11,26]. Risk factors for DKA include high levels of HbA1c, having diabetes for an extended period, being an adolescent, and being female. As noted by the T1D Exchange Clinic Network, young adults aged 18 to 25 years have the highest incidence (5%) of DKA [11,16,27]. HHS is a less common event than DKA, accounting for fewer than 1% of all diabetes-related hospitalizations [28,29,30]. However, the mortality rate from the condition ranges from 10% to 50%, compared to a significantly lower DKA mortality rate of 1.2% to 9% [21,31,32,33,34,35,36,37,38,39]. Older persons with T2DM are more likely to develop HHS [28].

### 1.4. Pathophysiology of Diabetic Ketoacidosis and Hyperglycemic Hyperosmolar State

DKA and HHS are two distinct conditions that share some common pathways leading to pathogenesis and clinical diagnosis, including elevated glucagon [15,18,29,40], but with some differences. Both complications are characterized by insulin deficiency (absolute insulin deficiency in the case of DKA and relative insulin deficiency in the case of HHS), hyperglycemia, urinary loss of water and electrolytes (sodium, potassium, chloride) and severe extracellular fluid volume (ECVF) depletion. ECFV depletion and hyperosmolarity are characteristics of HHS, whereas ketoacidosis is a hallmark of DKA [15]. Both conditions are also characterized by the presence of high levels of counter-regulated hormones [18,40], such as cortisol, catecholamines, glucagon and growth hormone.

In people with T1DM and insulin insufficiency, hyperglycemia reduces ECFV and leads to urinary losses of water and electrolytes such as salt, potassium, and chloride. As cellular depletion occurs, ketoacidosis, a hallmark of DKA, will develop due to the absence of insulin and high glucagon levels. High levels of catecholamine that suppress insulin release can also develop in T2DM patients, and can contribute to the development of HHS, which is characterized by ECFV depletion and hyperosmolarity [18,20].

### 1.5. Pathogenesis of Diabetic Ketoacidosis and Hyperglycemic Hyperosmolar State

The pathogenesis of DKA and HHS can be summarized as follows: (1) Islet cells in the pancreas are unable to produce sufficient insulin, leading to insulin deficiency. (2) This results in the liver producing more glucose via glycogenolysis and gluconeogenesis. (3) Glucose is filtered by the kidney and excreted in the urine. (4) The loss of water and electrolytes from the resultant osmotic diuresis causes extracellular fluid volume depletion and a resultant decrease in the glomerular filtration rate. (5) In addition, as an alternative source of energy, the body breaks down fat, leading to ketoacids accumulating in the bloodstream. The development of HHS is less acute than DKA, generally progressing slowly over a period of days or weeks. In patients with HHS, there is sufficient insulin to suppress lipolysis and the production of ketone bodies; however, the amounts of insulin are insufficient to prevent severe hyperglycemia, profound dehydration, and a more extreme mental health status than DKA [41]. As with DKA, steps 1–4 also occur in HHS, but this leads to hyperosmolality with minimal or no ketogenesis.

### 1.6. Management of Diabetic Ketoacidosis and Hyperglycemic Hyperosmolar State

The management of DKA or HHS events requires clinical assessment, laboratory evaluation, correction of volume depletion and electrolyte imbalance to restore normal ECFV and tissue perfusion, administration of insulin to lower glucose and resolve ketoacidosis (in the case of DKA) and treatment of coexistent illnesses [15].

### 1.7. Treatment with Antipsychotic Drugs and Diabetic Adverse Events

In recent years, case reports (CRs) have supported the potential association between the use of SGAs and DKA [42,43], while less information is available on HHS, with fewer documented cases on the topic. There have also been a handful of observational studies, both analytical and descriptive, that support this association [44].

Patients with serious mental illness, such as schizophrenia, that are treated with antipsychotic drugs are at an increased risk of hyperglycemic emergencies such as DKA [42]. Classic signs and symptoms of DKA and HHS, such as polyuria, polydipsia, nausea, vomiting, weakness, dehydration and altered mental status, have been described in clinical reviews of these two hyperglycemic crises [11,42]. These observations emphasize the importance of monitoring for indicators of DKA and HHS in psychiatric practice: more specifically, Whicher et al. (2019) [45] recommended routine monitoring of blood glucose and HbA1c levels for patients on antipsychotic treatment prior to treatment, after the initiation of treatment and following a change in treatment regimen (Table 2) as a preventative measure for serious complications. Access to appropriate healthcare services for people with schizophrenia, as well as the education of mental health practitioners, patients and their families about the signs and symptoms of diabetes, is essential to reduce the potential risks associated with APD use.

Although diabetic complications associated with antipsychotic medications have received growing attention, the literature on such complications remains scarce. Only a small number of observational studies—some of which are case series presenting aggregate data, rather than individual cases—have examined serious outcomes such as diabetic ketoacidosis (DKA) and hyperosmolar hyperglycemic state (HHS). Furthermore, while several systematic reviews have explored this association, none have comprehensively addressed HHS as a distinct clinical entity. The present review aims to fill this gap by systematically synthesizing published case reports and case series involving both first- and second-generation antipsychotics (FGAs and SGAs, respectively).

In this systematic review, we focus on individual case-level data in case reports and case series with epidemiological and clinical evidence for both DKA and HHS. The specific objective is to provide an in-depth review of these adverse events associated with APD use, both typical and atypical, and to identify clinical presentation patterns, observe temporal dynamics, and better understand the etiology of these two conditions.

## 2. Methods

### 2.1. Search Strategy and Databases

The search strategy was developed in consultation with a research librarian at the University of Ottawa’s Health Sciences Library to ensure the comprehensive and systematic coverage of the relevant literature. Although the review protocol was not preregistered, the methodology adhered to established standards for systematic reviews and followed PRISMA guidelines [46] (Supplemental Material I). A search of the four pertinent bibliographic databases (MEDLINE, EMBASE, the Cochrane Central Register of Controlled Trials (CENTRAL) and PsycINFO) was conducted to identify potentially relevant studies by using the relevant subject headings and keywords detailed in Supplemental Material II. The database searches identified records published up to 17 October 2025.

### 2.2. Data Screening and Abstraction

Duplicate publications were identified using Endnote (Clarivate Analyticis, Philadelphia, PA, USA) [47]. Screening of unique records was performed using a two-stage process: Level 1 (title and abstract) screening followed by Level 2 (full-text screening). Screening forms were created in Distiller SR (Evidence Partners, Ottawa, ON, Canada) [48] (Supplemental Material III). Relevant quantitative and qualitative data from all CRs included in the review were abstracted into an Excel spreadsheet, including demographic characteristics (gender, age, ethnicity), clinical information (diagnosis, treatment, history of DM, comorbidities, co-medications and prognosis) and quantitative and qualitative data on relevant biomarkers such as levels of glucose or hemoglobin A1C (HbA1c), body weight change, presence or absence of ketones and osmolality.

Two reviewers (NH and NF) independently carried out Level 1 and Level 2 screening in duplicate. In the case of conflicts, these were discussed between the two reviewers and resolved by a consensus. In cases where a consensus was not immediately reached, the first author made the final decision. Data abstraction for Level 2 screening (full-text) was primarily conducted by the first author (NH), with the second author (NF) independently reviewing 10% of full texts in duplicate as a quality control measure. The two reviewers maintained a high level of agreement (>90%) during the screening and data abstraction phases.

Observational studies that were eligible for analysis will be included in a separate review and were, therefore, excluded from the present review.

### 2.3. Eligibility Criteria

#### 2.3.1. Exposure Assessment of Antipsychotic Drugs

CRs with any APD monotherapy or concomitant use of APDs were included. FDA-FGAs or typical APDs considered included the following: Chlorpromazine, Droperidol, Fluphenazine, Haloperidol, Loxapine, Molindone, Perphenazine, Pimozide, Trifluoperazine, Prochlorperazine, Thioridazine and Thiothixene.

FDA-SGAs or atypical APDs included: Aripiprazole, Asenapine Maleate, Cariprazine, Clozapine, Iloperidone, Lurasidone, Olanzapine, Paliperidone, Pimavanserin, Quetiapine, Risperidone and Ziprasidone.

Although some authors classify certain agents (e.g., Aripiprazole, Cariprazine) as third-generation antipsychotics due to their partial D2 receptor agonist activity [1,2], we refer to them here within the broader second-generation category, which is consistent with traditional classifications. In addition, while the antipsychotic medications examined in the review are FDA-approved, the analysis encompassed case reports and case series from both US and non-US sources.

#### 2.3.2. Outcomes

CRs were included if one of the following outcomes—DKA, HHS, or DKA and HHS as co-conditions—were diagnosed.

#### 2.3.3. Study Design

Both CRs and case series (CSs) were included in the present review, with observational studies included in a companion review [44].

### 2.4. Inclusion and Exclusion Criteria

#### 2.4.1. Inclusion Criteria

CRs and CSs were included if they stated the use of at least one FDA-approved APD type and the evaluation of at least one of the outcomes of interest (DKA, HHS or DKA/HHS). CRs were only included if the article clearly indicated the diagnosis of DKA or HHS: reports describing information only on biomarkers (e.g., levels of glucose, HbA1c) without clinical diagnosis of one (or both) of these two conditions were excluded. Abstracts in both English and French were evaluated without restriction on geographic location. Conference abstracts that presented a comprehensive case report from which data could be extracted were considered for inclusion. This review covered patients of any age who had taken antipsychotic medications (typical and atypical); there were no restrictions on the indication of use of APDs or the age of the cases.

#### 2.4.2. Exclusion Criteria

CRs were excluded if the outcome of interest was not DKA or HHS, if the APD was never approved or no longer approved for use or if APDs were not the exposure of interest, i.e., suspected of causing the complication.

### 2.5. Biomarkers

Information on specific biomarkers such as plasma/serum or blood glucose (BG) levels, HbA1c levels, presence of ketones and description of osmolality was extracted when available. Glucose values reported in the included case reports were variably reported as blood glucose, serum glucose, plasma glucose or glucose. When the matrix for glucose measurement was reported as “blood”, we assumed this referred to whole blood glucose, which was consistent with point-of-care testing conventions. Since plasma or serum glucose measurements are considered the gold standard for diagnosing DKA and HHS, whole blood glucose measurements were converted using the standard conversion factor, i.e., plasma/serum glucose ≈ whole blood glucose × 1.12, expressed in mg/dL [49,50,51,52]. Values can be converted to the internationally recognized SI unit (mmol/L) by dividing by 18 [53]. HbA1c was measured using methods that were standardized to the International Federation of Clinical Chemistry and Laboratory Medicine (IFCC) reference system [54]. As such, results are reported in percentages (%) on the National Glycohemoglobin Standardization Program/Diabetes Control and Complications Trial (NGSP/DCCT) scale, which is derived from the IFCC reference method expressed in mmol/mol. Data permitting, the average levels of plasma/serum or BG and HbA1c were calculated for three distinct periods: (1) prior to the complication; (2) at the presentation of the complication; and (3) after the resolution of the complication. The final test results served as the foundation for all analyses, under the assumption that the most recent clinical values best reflect patient follow-up.

### 2.6. Critical Appraisal

The Joanna Briggs Critical Appraisal tool was used to evaluate the quality of information in each CR against eight criteria (Supplemental Materials IV and V). CRs were evaluated for completion on patient demographics, medical history, clinical conditions at presentation, diagnostic tests and the results, interventions or treatment post-intervention of the medical condition, identification of adverse or unanticipated events and lessons learned [55,56]. Critical appraisal for all CRs was performed by one reviewer (NH) and for a 10% sample of the included studies by a second reviewer (NF).

## 3. Results

### 3.1. Selection of Studies

**Figure 1:**
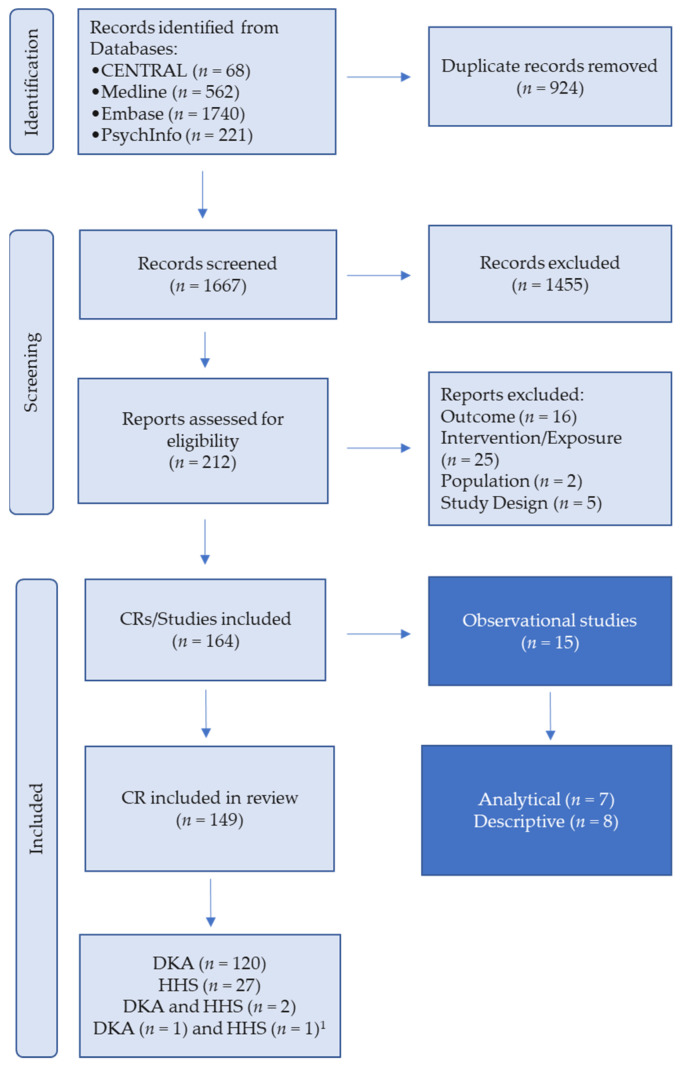
Flow chart for the selection of case reports included in the systematic review. Abbreviations: CR, case report; CENTRAL, Cochrane Central Register of Controlled Trials; DKA, diabetic ketoacidosis; HHS, hyperglycemic hyperosmolar state. ^1^ Two additional case reports (one DKA and one HHS) were identified through supplemental searching outside the predefined search strategy.

A total of 2591 potentially relevant reports and studies were identified by our database searches, although some of the case reports were embedded within larger case series. Of these, 151 CRs were included in the systematic review following a full-text review (Figure 1).

### 3.2. Demographic Characteristics

A total of 151 CRs summarized information on patients admitted to a hospital with a diagnosis of DKA or HHS who were treated with an APD marketed in the United States. Demographic characteristics (age, sex and ethnicity), diagnosis, treatment with APDs, history of diabetes, test results for relevant metabolic markers and treatment and recovery for each DKA or HHS patient are shown in Table 3 and Table 4, respectively, with further details provided in Supplemental Material V, Tables S2 and S3.

Among these CRs, 71.5% (*n* = 108) involved males and 27.2% (*n* = 41) were females. Of the 151 CRs, people from African descent and Caucasians accounted for 24.5% (*n* = 37) and 17.2% (*n* = 26) of CRs, respectively (Table 5). The highest proportion of complications attributed to an APD occurred in the 18–49-year age group: the 30–39-year age group accounted for 32.5% (*n* = 49) of all CRs, followed by the 18–29-year age group at 23.8% (*n* = 36) and the 40–49-year age group at 22.5% (*n* = 34). DKA was notably more common than HHS, accounting for about 81.5% (*n* = 123) of all CRs, while HHS accounted for 19.9% (*n* = 30). Two CRs described the simultaneous occurrence of DKA and HHS. One case involved a 28-year-old obese Caucasian male (body mass index (BMI) of 33.2 kg/m^2^) with a strong family history of T2DM and a high-sugar diet who was receiving Risperidone (Hui Fang et al. 2018) [94]. Similarly, Lim et al. (2025) [112] reported a co-occurrence in a 30-year-old male treated with Olanzapine. Both case reports were therefore included in subsequent analyses of DKA and HHS cases. While the case by Adhoni et al. (2021) [57] described HHS features alongside DKA, the primary clinical diagnosis was DKA and was therefore only included in the DKA analysis.

As mentioned previously (Table 5), single use of antipsychotic medicine was assumed unless APD polypharmacy was clearly stated at the time of presentation. The use of a single APD accounted for the majority of CRs at 72.8% (*n* = 110), while the concurrent use of multiple (2+) APDs accounted for 27.2% (*n* = 41) of CRs. APDs were prescribed to treat schizophrenia in 51.0% (*n* = 77) of reported cases, followed by bipolar disorder (17%, *n* = 26) and depressive disorders as a single diagnosis (7.3%, *n* = 11). Patients with two or more diagnoses accounted for 11.3% (*n* = 17) of reported CRs. These include a combination of conditions listed in Table 5, in addition to other conditions such as Alzheimer’s disease, borderline personality anxiety and mood disturbances. SGAs were the primary suspect drugs thought to result in the occurrence of DKA or HHS: Olanzapine was listed as the primary suspect drug in 39.1% (*n* = 59) of all CRs included in the analysis, followed by Clozapine (16.6%, *n* = 25), Quetiapine (14.6%, *n* = 22) and Risperidone (11.9%, *n* = 18).

Discontinuation of the APD suspected of causing the diabetic complication occurred in 70.2% (*n* = 106) of patients as a preventative measure. For some patients, treatment continued with a newly prescribed APD, resulting in switching of APDs in 30.5% of patients (*n* = 46). The most common comorbidities reported were other mental disorders, mood/behavioral disorders, asthma, myocardial infarction, hypertension and hyperlipidemia cited in 31.3% (*n* = 47) of CRs (Supplemental Tables S2 and S3). While considered a comorbidity, obesity is presented separately (Table 6). Overall, 13.2% (*n* = 20) of patients had a history of or were active alcohol and/or substance users at the time of the complication. Nine case reports indicated the use of tobacco with or without alcohol use or substance abuse (*n* = 9, 5.9%).

### 3.3. Specific Outcomes

#### Diabetic Ketoacidosis

DKA accounted for the great majority of CRs (*n* = 123, 81.5%). Patient demographics, mental health diagnosis, APD treatment and comorbidities and overall patient characteristics are shown in Table 5. The majority of patients were male (73.2%, *n* = 90). The 30–39-year age group accounted for the highest proportion of patients with DKA (32.5%, *n* = 40). People from African descent and Caucasians accounted for 26.8% (*n* = 33) and 17.1% (*n* = 21) of CRs, respectively. Schizophrenia was reported as being the main reason for APD use at 58.5% (*n* = 72), followed by bipolar disorder at 14.6% (*n* = 18). Olanzapine was the primary APD suspected to cause a DKA occurrence in 42.3% (*n* = 52) of CRs. Single APD use accounted for 72.4% (*n* = 89) of cases. Discontinuation of the suspected agent occurred in 69.9% of the cases (*n* = 86), while switching to another APD was reported in 33.3% (*n* = 41) of cases. About 27.6 percent of CRs (*n* = 34) noted concurrent comorbidities at the time of admission, and 19.5% (*n* = 24) of the affected individuals had a history of or were actively using alcohol, substances and/or tobacco.

### 3.4. Hyperglycemic Hyperosmolar State

HHS accounted for 19.9% of all cases (*n* = 30). Similarly to DKA, most cases occurred in the 30–39-year age group (33.3%, *n* = 10) and were male (66.7%, *n* = 20). Most CRs did not specify ethnicity. Patients had diagnoses of bipolar disorder (26.7%, *n* = 8), schizophrenia (20%, *n* = 6), depressive disorders (*n* = 3, 10.0%) and other psychotic conditions (*n* = 2, 6.7%). HHS occurrence was linked to treatment with Olanzapine in eight of the 30 CRs (26.7%) and to treatment with Quetiapine in eight further CRs. (Table 5).

### 3.5. History of Diabetes

CRs included information on patient and family history of diabetes and on relevant biomarkers, including levels of serum, plasma and BG; HbA1c concentration; presence/absence of ketones and serum osmolality. While the level of detail varied among CRs, the data were sufficient to support quantitative analysis on biomarkers such as plasma/serum glucose and HbA1c levels in three distinct periods: (1) prior to presentation with the condition, (2) at presentation with DKA or HHS, and (3) after treatment of either condition, or subsequent follow-up, with the latest test result being included. Of the 151 CRs, approximately one-fourth reported a history of family diabetes (*n* = 35, 23.2%), regardless of the patient’s own history. About 9.3% (*n* = 14) of CRs had a known patient history of DM, while 27.8% (*n* = 42) reported no patient history. Fifty-three (35.1%) CRs explicitly reported no patient or family history. Overall, 44.4% (*n* = 67) patients were diagnosed with T1DM, T2DM or new-onset DM, either prior to or after DKA or HHS. As discussed below, these results provide important insights into temporal changes in these key biomarkers (Table 6).

### 3.6. Relevant Biomarkers

#### Glucose

Plasma/serum glucose levels at presentation were available for most DKA cases (*n* = 114) and almost all HHS cases (*n* = 29). Over a third of CRs reporting qualitative information on serum glucose levels were “normalized” or “controlled” or “remained high” after the treatment and resolution of the complication (45.7%, *n* = 69). In CRs where DKA was the outcome, the average glucose level was 114.2 mg/dL among the subset of patients with available data prior to presentation (*n* = 29). At presentation or shortly after admission, the average glucose level was notably elevated at 842.8 mg/dL (*n* = 114). Following treatment for DKA, average plasma/serum glucose levels were 156.4 mg/dL (*n* = 47). In CRs where the patient suffered from HHS with the reported use of an APD, the plasma/serum glucose levels were 115.7 (*n* = 11), 1252.8 (*n* = 30) and 128.3 (*n* = 8) mg/dL before presentation, at presentation and after the resolution of HHS, respectively. These values represent different subsets of cases and should be interpreted as such (Table 6).

### 3.7. Hemoglobin A1c (HbA1c)

HbA1c levels reflect the average blood sugar levels over the past two to three months. For patients presenting with DKA, HbA1c levels were 6.5% (*n* = 12), 11.5% (*n* = 62) and 6.6% (*n* = 27) before presentation, at presentation or shortly thereafter, and after the resolution of the case, respectively. Similarly, HbA1c levels in patients presenting with HHS were 5.7% (*n* = 8), 12.0% (*n* = 17) and 6.2% (*n* = 11) during these same periods. These results should be interpreted with caution due to the low number of CRs reporting on this biomarker. However, the averages provide an indication of temporal changes in HbA1c over these three periods (Table 6).

### 3.8. Presence of Ketones

The presence of serum and urine ketones—a key indicator of DKA—was confirmed in 80.5% (*n* = 99) of DKA cases; no CR confirmed the absence of ketones; and 19.5% (*n* = 24) of DKA CRs did not specify whether ketones were present. Some CRs categorized the presence of ketones as mild, moderate, or severe, while others simply described them as positive or present. The occurrence of HHS may be accompanied by trace levels of ketones, as reflected in the qualitative description of some of the CRs: twelve HHS CRs had reported the presence of ketones, while another eleven CRs noted their absence (Supplemental Tables S2 and S3).

### 3.9. Treatment with Insulin and Recovery

Insulin was administered to 90.7% of patients (*n* = 137) to treat DKA or HHS (Supplemental Tables S2 and S3). Overall, about a quarter of all cases recovered fully (*n* = 37, 24.5%), and insulin and/or antihyperglycemic medications were discontinued within a few months of the complication episode. Partial recovery—defined as the patient requiring treatment with insulin or an antidiabetic due to a prior or new diagnosis of T1DM, T2DM or other types of DM, or the patient’s diabetic condition was controlled solely by diet—was noted in 58.3% of CRs (*n* = 88). Death was reported for twelve CRs (7.9%) (Table 6 and Table 7).

### 3.10. Olanzapine and Clozapine and Diabetic Ketoacidosis

Olanzapine and Clozapine are SGAs that were suspected of causing the highest proportion of DKA cases at 43.1% (*n* = 53) and 19.5% (*n* = 24), respectively. Schizophrenia accounted for the highest proportion of mental health diagnosis for Olanzapine at 51% (*n* = 27) and Clozapine at 87.5% (*n* = 21) of CRs. The time to complication from initiation of antipsychotic treatment, when reported, was estimated to be 6 months for Olanzapine and 2 months for Clozapine. Treatment with Quetiapine, Risperidone and Aripiprazole contributed to DKA within the period from 1 day to 36 months of initiation of therapy. The suspected APD was discontinued in 73.6% (*n* = 39) of Olanzapine-related and 66.7% (*n* = 16) of Clozapine-related DKA cases. Antipsychotic treatment was switched to another APD after DKA in 37.7% (*n* = 20) of Olanzapine cases and 37.5% (*n* = 9) Clozapine cases. Recovery from hyperglycemia was considered to be partial in most patients, in that almost 52.8 (*n* = 28) of patients receiving Olanzapine and 70.8% (*n* = 17) of patients who were treated with Clozapine were required to manage their existing DM condition or new-onset DM with oral hypoglycemics or diet management alone (Table 8).

### 3.11. Critical Appraisal of Case Reports

**Figure 2:**
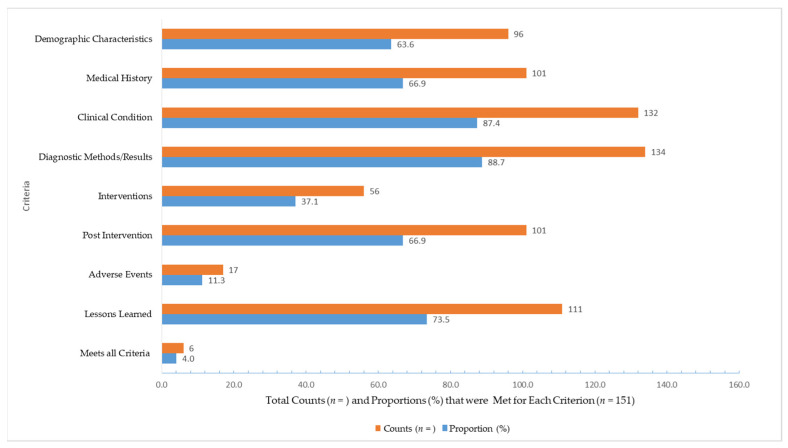
Overall results for the critical appraisal of case reports (*n* = 151) against eight criteria detailed in the Joanna Briggs Tool [55,56]. Cases where fatalities occurred could not be assessed for every criterion.

The Joanna Briggs Institute critical appraisal tools were used to assess the amount of information reported in all CRs (*n* = 151) on demographic details, medical history, clinical condition at presentation, methods and results for diagnosis, the intervention, post-intervention information, adverse treatment reactions and lessons learned. Six case studies (4%) satisfied all eight requirements. Most reports described the clinical condition (87.4%, *n* = 132) and diagnostic methods and results (88.7%, *n* = 134), while 37.1% (*n* = 56) of CRs provided details on the intervention used to resolve the complication. Only 11.3% (*n* = 17) of cases experienced an adverse event such as hypoglycemia after receiving treatment for DKA or HHS. The majority of CRs (73.5%, *n* = 111) suggested a path forward for lessons learned, including the routine monitoring of relevant biomarkers for patients who have been prescribed APDs (Figure 2, Supplemental Table S4).

## 4. Discussion

This article provides a comprehensive synthesis of the case reports and case series describing the risk of DKA or HHS attributed to treatment with an antipsychotic medication. Our analysis offers several novel contributions. First, we include data on hyperosmolar hyperglycemic state (HHS), which has not been systematically addressed in earlier syntheses. Second, we examine biomarker dynamics across three distinct time points—prior to presentation, at presentation and after resolution—providing a temporal perspective that enhances clinical insights. Third, we report on time to complication, offering a more granular understanding of disease progression. These elements, drawn from individual case reports and series, extend beyond the scope of prior reviews and contribute new perspectives on the association between use and these diabetic complications.

Our findings indicate that the development of antipsychotic-induced DKA appears to be primarily reported with the use of Olanzapine and Clozapine as a single medication or in combination with other APDs within the initial 12 months of therapy. Jin et al. (2004) [198] conducted a previous systematic review on the use of atypical APDs and glucose dysregulation and made the same observation about these two APDs, but within six months of initiation of therapy.

Our findings indicate that DKA and HHS events occurred predominantly in males and in the 30–39-year age group. These risk factors have been discussed by others in relation to DKA events, with variable patterns reported across studies [16,20,43,199]. Our findings indicate that African descent ethnicity was predominant among DKA cases, followed by Caucasian ethnicity. Recognizing that case reports do not represent population-level distributions, ethnic differences in risk should be investigated through large cohort studies that are designed to assess such associations. However, a recent population-based cross-sectional study in Croatia supports our results, and it estimated the age- and gender-adjusted incidence rate of DKA to be 17.0 per 100,000 person-years (95% CI: 14.9–19.4) in a well-defined, predominantly Caucasian population [200]. The rate was twice as high in males than in females (24.8 vs. 10.9) and primarily associated with T2DM. It is important to note that we cannot preclude biases that may affect the publication of case reports, including racial biases [201,202]. Prescription patterns for antipsychotic medications may also differ between countries and healthcare settings and may shift over time due to market influences [203].

Our results indicated that most patients who developed DKA had been diagnosed with schizophrenia and were being treated with olanzapine, an atypical APD. These findings were supported by a recent pharmacovigilance study by Sugawara et al. (2023) [204], using the Japanese Adverse Drug Event Report database. In their analysis of 55 reported cases of DKA in patients with schizophrenia, treatment with Olanzapine was associated with a significantly elevated reporting odds ratio (ROR) for DKA (adjusted ROR: 3.26; 95% CI: 1.87–5.66), and male sex was identified as a risk factor among Olanzapine-treated patients (adjusted ROR: 2.72, 95% CI: 1.07–6.90). People suffering from this severe mental illness have an elevated risk for diabetes [205,206,207]. One observational study showed that the incidence of DM presenting as DKA in patients with schizophrenia is 10-fold higher than in the general population [208]. It is also well established that metabolic syndrome in patients treated with APDs is due to insulin resistance and weight gain [209,210]. Insulin resistance can inhibit the response to APD treatment and may be an indicator of schizophrenia, independent of demographic factors (sex, age and gender) and lifestyle and clinical factors in first-episode antipsychotic-naive patients [211]. Our findings show that half of the cases reported involved patients who were previously diagnosed with schizophrenia (51%, *n* = 77). However, only 7.3% of DKA and 23.3% of HHS patients were reported to be diagnosed with DM prior to the complication. It remains possible that undiagnosed glucose abnormalities existed among the patients presented in this review. Yet, historical evidence, preceding the antipsychotic pharmacologic era, demonstrated that an abnormal glucose metabolism is greater in patients with schizophrenia [212,213,214]. Freyberg et al. (2017) [215] aimed to answer the fundamental question: “Is an intrinsic risk inherent to schizophrenia or is the metabolic phenotype primarily driven by the impact of APDs?” Based on their findings, the authors proposed that disease processes underlying neuropsychiatric symptoms such as cognition, executive function and sensory perception, which characterize schizophrenia, present a significant intrinsic risk for the development of metabolic abnormalities, including type 2 diabetes (T2D) and insulin resistance (IR). It was also suggested that these processes may also affect metabolically relevant peripheral areas such as the pancreas, liver and adipose tissue. The complexity of these issues is compounded by genetics and lifestyle [216,217,218], and there is now a consensus that APDs can exacerbate metabolic disturbances to different degrees. In our review, Olanzapine and Clozapine accounted for the majority of DKA cases. Clozapine and Quetiapine were reported in an equal number of HHS cases. Notably, in a retrospective cohort study, both Olanzapine and Clozapine have been associated with an increased risk of insulin resistance and major cardiovascular events of up to 2.8-fold [219]. The notion that metabolic disturbances may be intrinsically associated with schizophrenia is supported by a meta-analysis involving antipsychotic drug-naive patients, in which the prevalence of metabolic syndrome was 10.2%, compared to 19.4% and 47.2% for patients treated with Aripiprazole and Clozapine, respectively [220].

There is evidence that both Olanzapine and Clozapine are associated with prominent weight gain and metabolic adverse effects [209,221,222,223,224,225,226,227,228]. Previous reports suggested that both these atypical APDs may increase the risk of DKA [229,230]. In the present review, most cases presenting with DKA were attributed to treatment with Olanzapine, followed by Clozapine, while most HHS cases were either attributed to treatment with Olanzapine or Quetiapine. These findings are supported by our recent comprehensive analysis of spontaneous adverse reactions reports of DKA with APD medications using the US Food and Drug Administration’s Adverse Event Reporting System (FAERS) [231]. In this analysis, Olanzapine and Quetiapine were the most frequently reported atypical APDs, with disproportionality analysis demonstrating strong signals when compared to all other drugs (Olanzapine and Quetiapine demonstrated proportionate risk ratios (PRRs) with PRR = 13.2 (95% CI: 12.4–14.2) and PRR = 11.8 (95% CI: 11.1–12.5), respectively). A comparative review and meta-analysis conducted by Yu et al. (2019) [232] compared individual SGAs, i.e., Aripiprazole, Ziprasidone and Risperidone, to Olanzapine in Chinese patients with schizophrenia and found that Olanzapine was more likely to induce insulin resistance in these patients. In addition to a significantly higher insulin resistance index, patients treated with Olanzapine had significantly higher fasting blood glucose and fasting insulin levels than patients treated with the other SGAs. In contrast, Onda et al. (2025) [233] conducted a systematic review and meta-analysis of observational studies published between 2000 and 2025 that reported diabetes-related outcomes in patients treated with Olanzapine and Clozapine. Their analysis showed a significantly lower risk of new-onset diabetes associated with Olanzapine compared with Clozapine (seven studies; pooled HR = 0.73, 95% confidence interval [CI]: 0.63–0.85). However, results showed no significant difference between the two agents (nine studies; pooled OR = 0.85, 95% CI: 0.66–1.09). Taken together, these findings highlight the ongoing uncertainty regarding the relative diabetogenic risk of Olanzapine and Clozapine and underscore the need for further well-designed studies to clarify differences across metabolic endpoints, populations at risk and study designs. A recent systematic review by Alonso-Pedrero et al. (2019) [234] examined the association between the use of antidepressant and antipsychotic medications and weight gain in 27 cohort studies, which included children and adults. Results showed that both atypical antipsychotics (Quetiapine, Olanzapine, Clozapine, Risperidone and Aripiprazole) and typical antipsychotics (Haloperidol and Trifluoperazine) increased body weight by more than 7% from the baseline: a clinically significant result. Patients treated with antidepressants demonstrated a 5% weight gain. Furthermore, switching between antipsychotic medications can result in either weight gain or weight loss, although weight gain is more common [229], with antipsychotic-naive patients more likely to gain weight [209,235,236]. It is difficult to establish an accurate timeline for weight changes that are relative to APDs in the present review, since some patients had an elevated BMI prior to the occurrence of the complication, and others had an unknown BMI. However, given their psychiatric conditions, it can be inferred that most patients were not antipsychotic-naive.

In the CRs included in this review, the occurrence of DKA or HHS preceded the diagnosis of a diabetic condition in most reports. There could be several reasons for this. First, in the absence of an absolute or relative insulin deficiency in the patient, diabetic biomarkers were not previously assessed. Second, the potential for an increased risk of a hyperglycemic emergency due to treatment with an APD was not of concern at the time of treatment; therefore, glucose levels were not evaluated further. This is supported by the average glucose levels of 114.2 mg/dL and 115.7 mg/dL prior to presentation with DKA or HHS, respectively, in the CRs that provided this glycemic information. Third, as patient compliance to clinical follow-up may have been limited, there may have been reduced availability of diagnostic tests.

The diabetogenic properties of antipsychotic drugs are not fully understood. As such, the mechanisms of action by which Olanzapine and Clozapine may cause a hyperglycemic crisis remains unclear. However, it is hypothesized that these drugs may act through multiple pathways, including the strong antagonism of multiple receptors and the increased insulin resistance through the inhibition of pancreatic insulin secretion. Vuk et al. (2017) [43] even suggested that inpatients may already be experiencing insulin resistance prior to antipsychotic treatment, and the addition of antipsychotics may produce a state of functional insulinopenia, leading to the development of DKA. This is supported by the resolution of DKA and HHS following the discontinuation of treatment with APDs, dietary changes or administration of a hypoglycemic drug once insulin is withdrawn [59,76,108]. Our findings and previous reports [42] indicate that antipsychotic-induced DKA can occur after initiation of treatment, in the presence or absence of weight gain. There could be several explanations for new-onset DKA. First, patients may have a pre-existing T1DM condition that will result in the development of DKA. Second, T2DM may develop because of the higher BMI and insulin resistance induced by the drug. Third, sudden and severe new onset of DKA can imitate acute antipsychotic insulin resistance, unrelated to increased BMI [43].

This critical appraisal of case reports emphasizes the importance of educating APD users and clinicians on the potential risks associated with these agents. This view aligns with the American Diabetes Association’s (ADA) consensus [237] on the importance of monitoring metabolic risk factors for diabetes, as well as the importance of being cognizant of personal and family history of diabetes. Clinicians should remain vigilant about the clinical manifestations of hyperglycemic emergencies in high-risk patients. As summarized by Balhara (2011) [238], the management of diabetes in individuals with schizophrenia—and by extension, other severe mental illnesses—may be guided by recommendations from the Schizophrenia and Diabetes 2003 Expert Consensus Meeting [239]. These recommendations include the following: (1) the regular monitoring of baseline random or fasting plasma glucose and HbA1c in antipsychotic drug-naive patients starting on the medication; (2) routine medical evaluation for signs and symptoms of diabetes in non-diabetic patients, with the continued monitoring of glucose and HbA1c levels; and (3) a coordinated, multidisciplinary monitoring involving both endocrinologists and psychiatrists for the management of these two comorbidities in diabetic patients. Patient education coupled with clinical awareness by healthcare professionals and subsequent monitoring of the patient—regardless of diabetic status—could help to mitigate the precipitating factors leading to DKA or HHS occurrence and possible reoccurrence.

## 5. Strengths

Our systematic review provides a comprehensive analysis of CRs with DKA or HHS as the outcomes of interest, which were attributed to the use of at least one APD. The findings of the review are consistent with earlier studies of the etiology of DKA. To our knowledge, our review represents the first comprehensive analysis of CRs on HHS. Additionally, where data were available, we assessed pertinent biomarkers at three time points for each case: prior to presentation with the complication, during presentation and after resolution of the hyperglycemic crisis. These evaluations supported earlier data on changes in glucose and HbA1c values in patients hospitalized with these hyperglycemic emergencies. To shed light on diagnostic methods and the etiology of both conditions, we critically appraised each CR against eight specific criteria developed by the Joanna Briggs Institute. Our review provides insights into the diagnostic criteria for and management of DKA and HHS. Finally, case reports are valuable for informing and sensitizing the medical community to new and emerging issues [240]. They are also useful in the development of new hypotheses in drug safety surveillance [241] and may shed light on associations between intervention and outcome, which are relevant in the field of psychiatry [242]. From a clinical perspective, it would be beneficial to leverage the existing CR templates to standardize the relevance of their content. One such template is provided by the *British Medical Journal* [243], which includes the title of the case, a background summary, case presentation, investigations, differential diagnosis, treatment, outcome and follow-up, and a brief discussion with guidance for each section. Their template emphasizes the importance of obtaining patient consent and maintaining patient anonymity. This is significant for rare and life-threatening conditions such as DKA and HHS where experimental research with real-world patient populations is limited due to ethical constraints and limited study power.

## 6. Limitations

This review has several limitations. First, in interpreting glucose measurements, we assumed that CRs reporting blood glucose referred to whole blood glucose, resulting in an average glucose level of 842.8 mg/dL after standardizing to plasma/serum equivalents; since the average without standardization of 804.8 mg/dL is virtually unchanged and of little clinical significance, this assumption does not have a material impact on our findings. Second, the reliance on case reports introduces variability in the level of clinical detail and reporting quality. CRs have many limitations that should be noted [240], including the following: Population and generalization: CRs cannot generate quantitative epidemiological information and lack population-level representation; however, case reports may evolve into case series, prompting more informative analyses of aggregate information [240,244] and hypothesis testing through future experimental and non-experimental research and surveillance [198]. Retrospective nature: Information from each case report is consolidated after the occurrence of the outcome of interest: in this case, DKA or HHS. Undeniably, there are gaps in patient medical histories pertaining to relevant biomarkers, family history, or other information that would be useful in analyses of the type conducted here [240]. Biases: Due to their retrospective nature, CRs are more susceptible to recall and information biases [240]. Furthermore, publication bias remains a concern, as journals may favor positive outcomes over unfavorable or inconclusive ones [240]. Because they do not allow for causal inference, they should be complemented by non-experimental research—such as preclinical studies in animals or in vitro investigations—to support an accurate interpretation of the findings. Accrued publication of CRs can prompt safety communications from the FDA, if warranted, or consensus statements from the American Diabetes Association [237] regarding the use of atypical APDs and the metabolic syndrome (MetS), as well as recommendations for the clinical monitoring of relevant biomarkers for hyperglycemic emergencies [15]. Stronger measures can result in a discontinuation of the APD or a change in the treatment regimen, as reported in this review.

Third, outcome terminology such as “normalized” or “controlled” was used inconsistently across reports and often lacked objective criteria. These terms were reported verbatim in our synthesis but may reflect differing clinical thresholds, and the data should be interpreted with caution.

A limitation of the current review is the lack of a direct comparison with large cohort studies. Although outside the scope of the present review, these sources can provide valuable insights into the real-world risks of DKA and HHS associated with antipsychotic use. Future work integrating these diverse evidence streams is essential to fully elucidate the metabolic risks of antipsychotic agents. Each CR is written uniquely and with a varying level of information provided. Even though we exercised due diligence in extracting all pertinent information, it was challenging to create a precise timeline of events, including historical information regarding each case. We acknowledge the presence of data gaps related to patient history, quantitative measures, and condition management. There are also limitations in interpreting HHS data, considering the small number of CRs and the lack of previous reviews on this outcome. Nonetheless, CRs provide valuable information on the etiology of DKA and HHS and are valuable in the development of research hypotheses that can be examined in future studies.

## 7. Conclusions

This systematic review provides additional evidence of an association between APDs and two hyperglycemic complications: specifically, DKA and HHS. Further research is needed to elucidate the mechanisms of action of these drugs and ensure effective and safe use in patients who are susceptible to developing conditions associated with metabolic syndrome. The review findings offer important clinical insights into the etiology of these conditions in relation to APD use and underscore the need for a preventative approach to reducing hyperglycemic emergencies.

## Figures and Tables

**Table 1:** Diagnostic criteria for diabetic ketoacidosis and hyperglycemic hyperosmolar state in the United States ^1^.

Diagnostic Criteria	DKA			HHS
	Mild	Moderate	Severe	
Plasma glucose, mg/dL	>250	>250	>250	>600
(mmol/L)	(>13.9)	(>13.9)	(>13.9)	(>33.3)
Presence of ketones in urine or blood	Positive	Positive	Positive	Small traces
Confirmation of acidosis:				
Serum bicarbonate, mmol/L	15–18	10–<15	<10	>18
pH	7.25–7.30	7.00–<7.24	<7.00	>7.30
Anion gap	>10	>12	>12	<12
Mental health changes	Alert	Alert/drowsy	Stupor/coma	Stupor/coma

Abbreviations: DKA, diabetic ketoacidosis; HHS, hyperglycemic hyperosmolar sate. ^1^ Adapted from Kitabchi et al. (2006; 2009) [19,20].

**Table 2:** Recommended glucose and HbA1c screening intervals for patients on antipsychotic medication ^1^.

Antipsychotic Treatment	Glucose Monitoring
Prior to treatment with an APD	Fasting or random blood glucose and HbA1c before initiation
First year of treatment with an APD	1. Fasting or random blood glucose at 3 months 2. HbA1c at 6 months and 12 months
Established on an APD	HbA1c annually if previous result ≈ 6.0%
Change in APD treatment	HbA1c at 3, 6 and 12 months after change in treatment, then as per established

Abbreviations: APD, antipsychotic drug. HbA1c, hemoglobin A1c. ^1^ Adapted from Whicher et al. (2019) [45].

**Table 3:** Summary of the characteristics of patients diagnosed with diabetic ketoacidosis associated with antipsychotic drug use (*n* = 123) ^1–4^.

Patient No.	Citation	Age, Sex, Race	Diagnosis	Primary APD (Total Dose if Specified, mg/day)	Plasma/Serum Glucose ^3^ at Presentation (mg/dL)	HbA1c at Presentation (%)
1	Adhoni et al. (2021) [57]	21, M, African	PS	Olanz. (10)	575	12.5
2	Agrawal et al. (2016) [58]	45, M, African American	BD	Olanz.	896 (st)	NS
3	Ai et al. (1998) [59]	30, M, Afro-Caribbean	SCZ	Cloz. (300)	448.2	11
4	Akunjee et al. (2018) [60]	36, NS, African American	SCZ	Olanz.	NS	13.7
5	Al-Amri (2009) [61]	27, F, Arab	BD	Olanz. (10)	800	NS
6	Alex et al. (2018) [62]	38, M, Indian	SCZ, BD	Cloz. (250)	378	14
7	Almahmood et al. (2025) ^2^ [63]	69, M, NS	AD	Olanz. (10)	599.4	8.2
8	Ananth et al. (2004) [64]	46, M, NS	BD	Risp. (3)	1201	NS
9	Aruna and Paulose (1995) [65]	26, M, NS	BD	Chlorp. (1000)	NS	4.2
10	Atabay and Rodopman Arman (2019) [66]	17, M, NS	BD	Olanz. (15)	486.1 (st)	NS
11	Avella et al. (2004) (Case 1) [67]	37, F, African American	SCZ, BD, BPD	Olanz. (15)	N/A (vitreous postmortem)	NS
12	Avella et al. (2004) (Case 2) [67]	27, M, Caucasian	BD	Olanz. (7.5)	N/A (vitreous postmortem)	14.2 (postmortem)
13	Avella et al. (2004) (Case 3) [67]	34, M, Caucasian	SCZ	Olanz. (7.5)	N/A (vitreous postmortem)	14.7 (postmortem)
14	Avram et al. (2001) [68]	33, M, Caucasian	SCZ	Cloz. (100)	626	14
15	Bae et al. (2024) [69]	35, M, NS	SCZ	Palip. (234 mg/month) Queti. (400)	1086.4 (st)	14.4
16	Buch et al. (2003) [70]	42, M, Caucasian	SCZ	Olanz. (15)	720	NS
17	Cabrera et al. (2021) [71]	24, M, NS	SCZ	Fluph. (25 mg injection/2 weeks)	1700	NS
18	Cardinale et al. (2019) [72]	46, F, NS	DEP	Arip.	720	NS
19	Chellamuthu et al. (2010) [73]	42, M, Asian	SCZ	Risp.	1679.4	13.8
20	Cho and Lindenmayer (2009) [74]	45, F, Black	SCZ	Cloz.	448 (st)	NS
21	Church et al. (2005) [75]	34, F, African American	SCZ	Arip. (30)	577.8	NS
22	Colli et al. (1999) [76]	31, M, Caucasian	SCZ, BD	Cloz. (200)	756	NS
23	Courvoisie et al. (2004) [77]	7, M, Caucasian	BD	Olanz. (2.5)	302	NS
24	Croarkin et al. (2000) ^4^ [78]	42, M, Caucasian	MDD w/PS	Risp. (4)	565	11.4
25	Crown et al. (2007) [79]	NS, NS, African Canadian	DEP, ANX, PS	Olanz. (10)	806.4	14.9
26	Dahri and Brown (2002) [80]	22, M, Caucasian	SCZ	Olanz (10)/Risp. (8)	496.8	NS
27	Das et al. (2018) [81]	40, F, NS	BD	Quet. (400)	443	9.2
28	de Boer and Gaete (1992) [82]	44, M, NS	SCZ	Chlorp. (800)	1262 (st)	NS
29	Dhamija and Verma (2008) [83]	12, M, Caucasian	MD	Arip.	535.4 (st)	NS
30	Dibben et al. (2005) (Case 1) [84]	51, F, Caucasian	SCZ	Quet. (400)	1068.5 (st)	7.2
31	Dibben et al. (2005) (Case2) [84]	33, M, Asian	SCZ	Risp. (1.8)	1332.6 (st)	13.4
32	Doodnauth et al. (2021) [85]	29, M, African American	ASD	Risp.	1080	9.7
33	Fulbright and Breedlove, (2006) [86]	42, M, African American	SCZ	Olanz. (40)	1686	NS
34	Gandhi and Ganesh (2019) [87]	34, M, NS	BD, SCZ	Quet.	684.6 (st)	NS
35	Gatta et al. (1999) [88]	31, M, Caucasian	TRD	Olanz. (10)	648	14.7
36	Goldstein et al. (1999) (Case 1) [89]	42, F, Caucasian	SCZ	Olanz. (10)	882	11.6
37	Goldstein et al. (1999) (Case 2) [89]	40, F, Caucasian	SCZ	Olanz. (10)	1160	NS
38	Greenfield et al. (2002) [90]	38, M, NS	SCZ	Thior.	993.9 (st)	13.6
39	Hepburn and Brzozowska (2016) [91]	44, M, NS	SCZ	Cloz.	469.7 (st)	14.6
40	Hörber et al. (2018) [92]	52, F, NS	SCZ	Risp.	1889.4 (st)	12.2
41	Howes and Rifkin (2004) [93]	41, F, NS	SCZ	Olanz. (20)	1236.6	NS
42	Hui Fang et al. (2018) ^1^ [94]	28, M, NS	OCD	Risp. (1)	826.6 (st)	10.8
43	Hussain et al. (2024) [95]	35, M, NS	SCZ, BD	Arip.	591	16
44	Itoh et al. (2019) [96]	66, M, NS	DEP	Quet. (25)	1709	13.1
45	Iwaku et al. (2017) [97]	32, M, NS	SCZ	Olanz. (5)	490	15.5
46	Jain et al. (2024) [98]	73, F, African American	DEP w/PS	Olanz. (5)	748	11.8
47	Jalota et al. (2015) [99]	21, M, Asian	SCZ	Quet.	378	13.3
48	Johnson et al. (2002) [100]	49, M, Caucasian	SCZ	Olanz. (20)	368	NS
49	Juneja et al. (2021) [101]	31, M, NS	SCZ	Olanz.	952 (st)	14
50	Kahn and Bourgeois, (2007) [102]	29, M, African American	SCZ	Olanz. (30)	1652	15.2
51	Kanagaretnam et al. (2022) [103]	31, F, NS	SCZ	Cloz. (300)	154.6 (st)	6.2
52	Kasmi (2013) [104]	29, M, Arab	NS	Cloz. (275)	NS	NS
53	Kibbey et al. (2010) [105]	29, M, Asian	SCZ	Arip. (40)	874.9 (st)	15.9
54	Kinoshita et al. (2014) [106]	29, M, Asian	DEP	Olanz. (2.5)	925.2 (st)	9.6
55	Kostakoğlu et al. (1996) [107]	42, M, NS	SCZ	Cloz. (350)	447	NS
56	Koval et al. (1994) [108]	34, F, African American	SCZ	Cloz. (250)	1224	NS
57	Kyriazis et al. (2006) [109]	33, M, Caucasian	PS	Olanz. (20)	478	NS
58	Lafayette et al. (2003) [110]	22, F, Hispanic/ Italian	SCZ	Cloz. (150)	512	12.2
59	Laghate and Gupta (2004) [111]	31, M, NS	BD	Chlorp. (200)/Halop. (10)	580	NS
60	Lim et al. (2025) ^1^ [112]	30, M, NS	SCZ	Olanz.	1926.4 (st)	13
61	Lindenmayer and Patel (1999) [113]	50, M, African American	SCZ, ASPD	Olanz. (30)	1344 (st)	NS
62	Lu and Yan (2009) [114]	27, M, NS	SCZ	Risp.	1297	13.7
63	Macfarlane and Fisher (2006) [115]	33, M, NS	SCZ	Olanz. (10)	1422	NS
64	Madsen (2014) [116]	27, M, NS	ANX	Quet. (400)	564.5 (st)	NS
65	Mahmoud et al. (2023) [117]	28, M, NS	BD	Olanz.	735	NS
66	Makhzoumi et al. (2008) [118]	44, M, African American	SCZ	Arip. (30)	813	14.9
67	Maksimoviæ and Pavliæ-Renar (2006) [119] (Case 2)	29, M, Caucasian	SCZ	NS	610.9 (st)	12.9
68	Marlowe et al. (2007) [120]	45, M, NS	SCZ	Quet. (800)	1492	NS
69	Maule et al. (1999) [121]	50, F, Caucasian	SCZ	Cloz. (400)	1000	NS
70	McCalmon and Weide (2021) [122]	23, M, African Amercian	SCZ	Cloz.	547.7 (st)	12.3
71	Miller (2008) [123]	56, M, African American	SCZ	Loxap. (100)	722	NS
72	Mithat et al. (2005) [124]	37, M, NS	BD	Risp. (4)	647	NS
73	Mohan (1999) [125]	30, M, Black	SCZ	Cloz. (325)	383 (st)	NS
74	Muench and Carey (2001) [126]	38, M, Caucasian	SCZ	Olanz. (20)	765	13.4
75	Murakami et al. (2025) [127]	58, F, NS	SCZ	Olanz. (12.5)	919	17.6
76	Nagamine (2021) [128]	31, M, Asian	SCZ	Olanz. (20)	545.4	7.6
77	Nahas et al. (2010) [129]	45, F, NS	BD	Olanz.	715	10.3
78	Nakanishi et al. (2025) [130]	49, M, NS	BD	Quet. (300)	284	12.8
79	Ng and Broussard (2024) [131] (Abstract)	66, F, NS	BD	Olanz. (5)	718	14
80	Niazy et al. (2007) [132]	28, M, Arab	SCZ	Olanz.	794.3 (st)	NS
81	Nicolai et al. (2001) [133]	33, M, Indian	SCZ	Cloz. (450)	1717.2	NS
82	Ogunnaya et al. (2024) [134]	58, M, NS	Dementia	Risp.	576.8 (st)	NS
83	Patel et al. (2011) [135] (Case 1)	32, M, NS	SCZ	Arip.	1249.9 (st)	NS
84	Pathmanathan and Somasekharan (2013) [136]	53, M, NS	SCZ	Quet. (600)	452	14.2
85	Peterson and Byrd (1996) [137]	46, M, African American	SCZ	Cloz. (500)	762	NS
86	Pierides (1997) [138]	50, M, NS	SCZ	Cloz. (300)	423	NS
87	Pillai et al. (2006) [139]	45, M, NS	SCZ	Cloz.	465.9 (st)	8.9
88	Popli et al. (1997) [140]	32, M, Black	SCZ	Cloz. (425)	930	NS
89	Ragucci and Wells (2001) [141]	46, F, African American	BD, DEP	Olanz. (15)	1071.8 (st)	11.7
90	Rahat et al. (2005) [142]	28, M, NS	SCZ	Olanz.	616 (st)	NS
91	Rashid et al. (2009) [143]	30, F, Asian	SCZ	Quet. (200)	1210.7 (st)	NS
92	Ratnakaran (2015) [144]	68, M, NS	DEP	Quet. (25)	363	NS
93	Reddymasu et al. (2006) [145]	33, M, African American	SCZ	Arip.	1981.3 (st)	NS
94	Sa et al. (2013) [146]	29, M, NS	PRD, HYP, PS	Olanz. (10)	1216	13.8
95	Sato et al. (2008) [147]	46, F, Asian	SCZ	Risp. (3)	926	12.2
96	Seaburg et al. (2001) [148]	27, M, African American	SCZ	Olanz. (10)	1388.8 (st)	NS
97	Selva and Scott (2001) [149]	16, F, Hispanic	MDD, AVH	Olanz. (15)	669	17.7
98	Shin et al. 2025 [150]	23, M, NS	SCZ	Cloz. (100)	500	11.5
99	Singh et al. (2013) [151]	35, M, NS	SCZ	Olanz. (10)	625	NS
100	Sirois (2008) [152]	41, F, Black	DEP w/PS	Quet. (400)	1472.4	NS
101	Skwiersky et al. (2020) [153]	20, M, NS	SCZ	Olanz. (15)	519.7 (st)	20.1
102	Smith et al. (1999) [154]	40, M, Afro-Caribbean	SCZ	Cloz.	1108.8 (st)	NS
103	Straker et al. (2002) [155]	44, F, African American	SCZ	Olanz. (20)	560 (st)	5.9
104	Strassnig et al. (2013) (Case 1) [156]	25, F, Canadian Aboriginal	SCZ	Cloz.	660.6	NS
105	Takahashi et al. (2005) [157]	72, M, Asian	Dementia	Quet. (50)	973	NS
106	Taslipinar et al. (2008) [158]	26, M, NS	SCZ	Risp. (6)	338	11.2
107	Tavakoli and Arguisola (2003) [159]	35, M, Caucasian	BD	Olanz. (5)	NS	NS
108	Thanikonda et al. (2020) [160]	32, F, African American	BD	Zipra.	1247.7 (st)	NS
109	Torrey and Swalwell (2003) [161]	45, M, NS	BD	Olanz. (30)	N/A (vitreous postmortem)	NS
110	Tsuchiyama et al. (2004) [162]	28, M, Asian	SCZ	Olanz. (10)	1209.6 (st)	13.7
111	Tugwell et al. (2020) [163]	28, F, African	PM, PS	Olanz. (40)	896.4	15.8
112	Varma et al. (2007) [164]	35, F, NS	BD	Olanz. (10)	1844.6 (st)	13.2
113	Vincent et al. (2017) [165]	39, M, NS	BD, SCZ	Risp. (3)	457	15.3
114	Vuk et al. (2017) [43]	33, M, Caucasian	SCZ	Cloz. (200)	1224.1 (st)	8.5
115	Waldman and Yaren (2002) [166]	33, M, Aboriginal	SCZ	Olanz. (30)	756 (st)	NS
116	Watkins et al. (2011) [167]	55, M, African American	DEP	Arip. (10)	799.7 (st)	13.5
117	Whicher et al. (2019) [45]	28, M, NS	SCZ	Olanz. (10)	483.8 (st)	NS
118	Wilson et al. (2002) (Case 1) [168]	48, M, African American	PS	Olanz. (30)	404.3 (st)	NS
119	Wilson et al. (2002) (Case 2) [168]	38, F, African American	SCZ	Olanz. (15)	647.4 (st)	NS
120	Wilson et al. (2002) (Case 3) [168]	64, M, Caucasian	SCZ	Quet. (40)	NS	NS
121	Wilson et al. (2002) (Case 4) [168]	26, F, African American	SCZ	Risp. (3)	489.4 (st)	NS
122	Wilson et al. (2002) (Case 5) [168]	33, M, African American	SCZ	Cloz. (550)	355 (st)	NS
123	Wong et al. (2007) [169]	22, M, Asian	SCZ, DPD	Olanz. (10)	721.8	11.9

Abbreviations: APD, antipsychotic drug; HbA1c, hemoglobin A1c; M, male; F, female; NS, not specified; w, with; AD, Alzheimer’s disease; ANX, anxiety; ASD, autism spectrum disorder; ASPD, antisocial personality disorder; AVH, auditory and visual hallucinations; BD, bipolar disorder; BPD, borderline personality disorder; DEP, depression; DPD, dissocial personality disorder; HYP, hypochondriasis; MD, mood disturbances; MDD, major depressive disorder; OCD, obsessive–compulsive disorder; PM, postpartum mania; PRD, personality disorder; PS, psychotic disorder or psychotic features; SCZ, schizophrenia or schizoaffective disorder; TRD, treatment refractory disorder; Arip, Aripiprazole; Chlorp, Chlorpromazine; Cloz, Clozapine; Fluph, Fluphenazine; Halop, Haloperidol; Olanz, Olanzapine; Loxap, Loxapine; Palip, Paliperidone; Quet, Quetiapine; Risp, Risperidone; Thior, Thioridazine; Zipra, Ziprasidone. ^1^ Two cases were diagnosed as demonstrating both DKA and HHS and are included [94,112]. ^2^ One DKA case was identified through supplemental searching outside the predefined search strategy. ^3^ Standardized blood glucose values are shown (st). ^4^ Added after cross-referencing with Vuk et al. (2017) [43].

**Table 4:** Summary of the characteristics of patients diagnosed with hyperglycemic hyperosmolar state associated with antipsychotic drug use (*n* = 30) ^1−3^.

Patient No.	Citation	Age, Sex, Race	Diagnosis	Primary APD (Total Dose if Specified, mg/day)	Plasma/Serum Glucose ^3^ at Presentation (mg/dL)	HbA1c at Presentation (%)
1	Ahuja et al. (2010) [170]	35, F, Caucasian	BD	Olanz. (10)	1647	13.2
2	Balzan and Cacciottolo (1992) [171]	50, M, Caucasian	DEP w/PS	Halop. (20)	907.2 (st)	NS
3	Campanella et al. (2009) [172]	48, M, NS	SCZ, DEP	Arip. (30)	2845	NS
4	Cerimele (2008) [173]	39, M, NS	SLPE	Risp. (4)	1179	14.6
5	Chen et al. (2003) [174]	33, M, Asian	BD	Olanz. (5)	1121	13.6
6	Chen et al. (2011) [175]	39, M, NS	BD	Quet. (100)	815	13.3
7	Cheslock et al. (2022) ^2^ [176]	96, F, NS	AD	Quet. (12.5)	864.6 (st)	NS
8	Endoh et al. (2012) [177]	36, M, Asian	NA	Olanz. (7.5)	1797.6 (st)	12.3
9	Franco et al. (2012) [178]	66, F, Hispanic	AD	Quet. (100)	957	NS
10	Franco et al. (2015) [179]	50, M, Caucasian	BD	Quet.	597 (st)	10.4
11	Hanyu et al. 2022 [180]	81, M, NS	BD	Luras. (20)	698	NS
12	Hui Fang et al. (2018) ^1^ [94]	28, M, NS	OCD	Risp. (1)	826.6 (st)	10.8
13	Kaino et al. (2017) [181]	27, F, NS	PD	Olanz. (20)	762	13.4
14	Kaya et al. (2014) [182]	67, M, NS	DEP w/PS	Quet. (200)	776	12
15	Khan et al. (2011) [183]	36, M, NS	MDD	Quet. (400)	2198	NS
16	Khanal et al. (2020) [184]	67, M, African American	PS	Risp.	1447 (st)	10
17	Létourneau et al. (2011) [185]	26, M, Caucasian	SCZ	Zipras. (160)	1371 (st)	13.5
18	Lim et al. (2025) ^1^ [112]	30, M, NS	SCZ	Olanz.	1720	13
19	Maust et al. (2015) [186]	19, M, African American	SCZ	Perph. (8)	995	9.7
20	McCall and Bourgeois (2004) [187]	35, F, African American	BD	Olanz. (10)	2023.5 (st)	NS
21	Meatherall et al. (2002) [188]	31, M, NS	SCZ	Olanz. (20)	284	12.3
22	Milano et al. (2016) [189]	41, F, NS	BD	NS	1403	NS
23	Raza S. (2007) [190]	37, F, NS	PS	Quet.	2278.1 (st)	NS
24	Rock et al. (2009) [191]	25, M, NS	SCZ, M.S.	Halop.	880	NS
25	Roefaro and Mukherjee (2001) [192]	51, M, Caucasian	PTSD, BD	Olanz. (25)	1596	13.3
26	Short and Nolan (1995) [193]	61, F, NS	SCZ	Cloz. (250)	1541	NS
27	Takanobu et al. (2015) [194]	47, M, NS	SCZ	Risp. (6)	449.1 (st)	7
28	Tollefson et al. (1983) [195]	49, F, NS	BD	Loxap. (150)	1055 (st)	NS
29	Vakharia et al. 2022 [196]	17, F, Hispanic	Mood disorder/ aggression	Risp.	844	11.5
30	Yeung and Lee. (2015) [197]	45, M, African American	NS	Quet. (400)	1568 (st)	NS

Abbreviations: APD, antipsychotic drug; HbA1c, hemoglobin A1c; M, male; F, female; NS, not specified; w, with; AD, Alzheimer’s disease; BD, bipolar disorder; DEP, depression; MDD, major depressive disorder; M.S., Munchausen syndrome; NA, narcolepsy; OCD, obsessive–compulsive disorder; PD, panic disorder; PTDS, post-traumatic stress disorder; PS, psychotic disorder or psychotic features; SCZ, schizophrenia or schizoaffective disorder; SLPE, schizophrenia-like psychosis of epilepsy; Arip, Aripiprazole; Cloz, Clozapine; Halop, Haloperidol; Loxap, Loxapine; Luras, Lurasidone; Olanz, Olanzapine; Quet, Quetiapine; Perph, Perphenazine; Risp, Risperidone; Zipra, Ziprasidone; ^1^ Two cases were diagnosed as demonstrating both DKA and HHS and are included [94,112]. ^2^ One HHS case was identified through supplemental searching outside the predefined search strategy. ^3^ Standardized blood glucose values are shown (st).

**Table 5:** Descriptive statistics of demographic characteristics, diagnosis, antipsychotic drug use, comorbidities and other issues in patients diagnosed with diabetic ketoacidosis (*n* = 123) or hyperglycemic hyperosmolar state (*n* = 30) case reports ^1–7^.

Characteristic	DKA (*n* = 123) ^1,2^	HHS (*n* = 30) ^1,2^	Overall (*n* = 151) ^2^
Biological Sex (*n*, %)			
Male	90 (73.2)	20 (66.7)	108 (71.5)
Female	31 (25.2)	10 (33.3)	41 (27.2)
NS	2 (1.6)	0 (0.0)	2 (1.3)
Age, y (*n*, %)			
<18	4 (3.3)	1 (3.3)	5 (3.3)
18–29	32 (26.0)	5 (16.7)	36 (23.8)
30–39	40 (32.5)	10 (33.3)	49 (32.5)
40–49	29 (23.6)	5 (16.7)	34 (22.5)
50–59	10 (8.1)	3 (10.0)	13 (8.6)
60–69	5 (4.1)	4 (13.3)	9 (6.0)
>69	2 (1.6)	2 (6.7)	4 (2.6)
NS	1 (0.8)	0 (0.0)	1 (0.7)
Mean Age [95% CI]	37 ± 12.3 [95% CI: 34.8–39.2]	43.7 ± 18.1 [95% CI: 37–50.2]	
Race ^3^ (*n*, %)			
African Descent ^4^	33 (26.8)	4 (13.3)	37 (24.5)
Caucasian	21 (17.1)	5 (16.7)	26 (17.2)
Asian	11 (8.9)	2 (6.7)	13 (8.6)
Other	9 (7.3)	2 (6.7)	11 (7.3)
NS	49 (39.8)	17 (56.7)	64 (42.4)
Mental Health Diagnoses (*n*, %)			
Schizophrenia	72 (58.5)	6 (20)	77 (51.0)
Bipolar Disorder	18 (14.6)	8 (26.7)	26 (17.2)
Depressive Disorders	8 (6.5)	3 (10.0)	11 (7.3)
Psychosis Disorders	3 (2.4)	2 (6.7)	5 (3.3)
Other Diagnoses ^5^	8 (6.5)	6 (20.0)	13 (8.6)
Two or More Diagnoses ^6^	13 (10.6)	4 (13.3)	17 (11.3)
NS	1 (0.8)	1 (3.3)	2 (1.3)
Primary APD Causing Complication (*n*, %)			
Atypical Antipsychotics			
Olanzapine	52 (42.3)	8 (26.7)	59 (39.1)
Clozapine	24 (19.5)	1 (3.3)	25 (16.6)
Quetiapine	14 (11.4)	8 (26.7)	22 (14.6)
Risperidone	14 (11.4)	5 (16.7	18 (11.9)
Aripiprazole	9 (7.3)	1 (3.3)	10 (6.6)
Ziprasidone	1 (0.8)	1 (3.3)	2 (1.3)
Lurasidone	0 (0.0)	1 (3.3)	1 (0.7)
Typical Antipsychotics	5 (4.1)	4 (13.3)	9 (6.0)
Combination of Two APDs	3 (2.4)	0 (0.0)	3 (2.0)
NS	1 (0.8)	1 (3.3)	2 (1.3)
Type of APD Therapy ^7^ (*n*, %)			
Monotherapy	89 (72.4)	22 (73.3)	110 (72.8)
Polypharmacy	34 (27.6)	8 (26.7)	41 (27.2)
AAP Discontinued (*n*, %)			
Yes	86 (69.9)	22 (73.3)	106 (70.2)
No	17 (13.8)	0 (0.0)	17 (11.3)
NS/N/A	20 (16.3)	8 (26.7)	28 (18.5)
Antipsychotic Switching (*n*, %)			
After DKA/HHS	41 (33.3)	6 (20.0)	46 (30.5)
No	37 (30.1)	9 (30.0)	45 (29.8)
Other (NS, U, N/A)	45 (36.6)	15 (50.0)	60 (39.7)
Comorbidities Prior to Event (*n*, %)			
Yes	34 (27.6)	14 (46.7)	47 (31.1)
No	29 (23.6)	6 (20.0)	35 (23.2)
NS	60 (48.8)	10 (33.3)	69 (45.7)
Other Issues (*n*, %)			
Substance/Alcohol Abuse	15 (12.2)	5 (16.7)	20 (13.2)
Tobacco Use	7 (5.7)	0 (0.0)	7 (4.6)
Tobacco Use and Alcohol Abuse/Tobacco Use and Substance Abuse	2 (1.6)	0 (0.0)	2 (1.3)
No	38 (30.9)	10 (33.3)	48 (31.8)
NS	61 (49.6)	15 (50.0)	74 (49.0)

Abbreviations: APD, antipsychotic drug; CI, confidence interval; DKA, diabetic ketoacidosis; HHS, hyperglycemic hyperosmolar state; N/A, not applicable; NS, not specified; U, unsure; y, years. ^1^ Two cases were diagnosed as demonstrating both DKA and HHS and are included in both columns [94,112]. ^2^ Proportions are calculated based on total DKA reports (*n* = 123), HHS reports (*n* = 30), or the total count of unique cases in the overall column (*n* = 151). ^3^ Other races include Aboriginal, Arab, Hispanic, Indian, Italian and mixed race. ^4^ African Descent includes African, African American, African Canadian, Afro-Caribbean and Black. ^5^ Other diagnoses include aggression, Alzheimer’s disease, anxiety, mood disorders, obsessive–compulsive disorder, panic disorder, schizophrenia-like psychosis of epilepsy, and treatment refractory disorder (see Table 3 and Table 4). ^6^ Two or more diagnoses may include a combination of those listed in the table in addition to antisocial personality disorder, auditory and visual hallucinations, borderline personality disorder, dissocial personality disorder, hypochondriasis, post-partum mania, and personality disorder. ^7^ APD monotherapy was assumed unless polypharmacy at the time of presentation was clearly stated in the report.

**Table 6:** Descriptive statistics of history of diabetes and relevant biomarkers for diabetic ketoacidosis (*n* = 123) and hyperglycemic hyperosmolar state (*n* = 30) in case reports ^1–6^.

Characteristic	DKA (*n* = 123) ^1,2^	HHS (*n* = 30) ^1,2^
	**Counts (%)**	
History of Diabetes as Detailed in the Case Report ^3^ (*n*, %)		
Family history	30 (24.4)	6 (20.0)
Patient history	9 (7.3)	5 (16.6)
Patient and family history	2 (1.6)	0 (0.0)
No family history	2 (1.6)	2 (6.6)
No patient history	37 (30.1)	5 (16.6)
No patient or family history	46 (37.4)	7 (23.3)
NS	11 (8.9)	6 (20.0)
Diagnosis with DM (*n*, %)		
Prior to complication	10 (8.1)	7 (23.3)
After complication	41 (33.3)	9 (30)
Total diagnosis with DM (prior and after complication)	51 (41.5)	16 (53.3)
Case Reports with Reported Plasma/Serum Glucose Levels in mg/dL		
Before complication	114.2 ±25 [95% CI: 104.5–123.9]	115.7 ± 39.4 [95% CI: 86.9–146.3]
Avg. plasma/serum glucose ± SD	(*n* = 29)	(*n* = 11)
At presentation	842.8 ± 413.3 [95% CI: 776.2–919.5]	1252.8 ± 593.3 [95% CI: 1027.1–1478.5]
Avg. plasma/serum glucose ± SD	(*n* = 114)	(*n* = 30)
After resolution	156.4 ± 67.7 [95% CI: 136.3–176.5]	128.3 ± 33.9 [95% CI: 100–156.6]
Avg. plasma/serum glucose ± SD	(*n* = 47)	(*n* = 8)
HbA1c test		
Before complication	6.5 ± 1.87 [CI: 5.2–7.7]	5.7 ± 0.80 [CI: 5.0–6.4]
Avg. HbA1c ± SD	(*n* = 12)	(*n* = 8)
At presentation	11.5 ± 3.02 [CI: 10.8–12.3]	12.0 ± 1.91 [CI: 11.1–12.9]
Avg. HbA1c ± SD	(*n* = 62)	(*n* = 17)
After resolution	6.6 + 1.77 [CI: 5.9–7.3]	6.2 ± 0.74 [CI: 5.9–6.5]
Avg. HbA1c ± SD	(*n* = 27)	(*n* = 11)
Presence of Blood or Urine Ketones (*n*, %)		
Ketone positive	99 (80.5)	12 (40.0)
Ketone negative	0 (0.0)	11 (36.7)
NS/NA	24 (19.5)	7 (23.3)
Weight ^4,5^ (*n*, %)		
Overweight/Obese	61 (49.6)	12 (40)
Weight ↑	33 (26.8)	5 (16.7)
Weight ↓	19 (15.4)	2 (6.7)
No change	13 (10.6)	3 (10.0)
Change NS	58 (47.2)	20 (66.7)
Type of Recovery ^6^ (*n*, %)		
Full	33 (26.8)	4 (13.3)
Partial	69 (56.1)	20 (66.7)
Fatal	8 (6.5)	4 (13.3)
NS/U	13 (10.6)	2 (6.7)

Abbreviations and symbols: Avg, average; CI, confidence interval; DKA, diabetic ketoacidosis; DM, Diabetes Mellitus; HHS, hyperglycemic hyperosmolar state; NS, not specified; SD, standard deviation; U, unsure; ↑, increase; and ↓, decrease. ^1^ Two cases were diagnosed as demonstrating both DKA and HHS and are included in both columns [94,112]. ^2^ Proportions are calculated based on total DKA reports (*n* = 123) and HHS reports (*n* = 30). ^3^ For all CRs, history of diabetes was extracted as explicitly reported. Categories are not mutually exclusive; for example, a CR may report no patient history while reporting a positive family history. Therefore, counts do not sum to the total number of DKA or HHS cases. ^4^ Based on the qualitative description in the CR, or the BMI when available. ^5^ Weight categories are not mutually exclusive; for example, a CR may report an increase in weight and that the patient is obese. Therefore, counts do not sum to the total number of DKA or HHS cases. ^6^ Full recovery: the patient would not require antidiabetic or insulin therapy in the long term, and treatment to manage any diabetes condition would be reduced and eventually stopped fully in the months after the resolution of DKA or HHS. Partial recovery: the patient was on antidiabetics to treat any diabetes condition prior to, or after the occurrence of, DKA or HHS. In addition, this includes patients where DM was managed by diet or exercise.

**Table 7:** List of case reports where fatalities occurred (*n* = 12) ^1^.

Citation	Primary APD/Outcome
Avella et al. (2004) [67] (Case 1)	Olanzapine/DKA
Avella et al. (2004) [67] (Case 2)	Olanzapine/DKA
Avella et al. (2004) [67] (Case 3)	Olanzapine/DKA
de Boer and Gaete (1992) [82]	Chlorpromazine/DKA
Khan et al. (2011) [183]	Quetiapine/HHS
Lu and Yan (2009) [114]	Risperidone/DKA
Madsen (2014) [116]	Quetiapine/DKA
Meatherall et al. (2002) [188]	Olanzapine/HHS
Milano et al. (2016) [189] ^1^	NS/HHS
Pillai et al. (2006) [139]	Clozapine/DKA
Rock et al. (2009) [191]	Haloperidol/HHS
Torrey and Swalwell (2003) [161]	Olanzapine/DKA

Abbreviations: APD, antipsychotic drug; DKA, diabetic ketoacidosis; HHS, hyperglycemic hyperosmolar state; NS, not specified. ^1^ Death occurred 5 months after HHS occurrence.

**Table 8:** Descriptive statistics for Olanzapine or Clozapine-induced diabetic ketoacidosis case reports ^1–6^.

	DKA	
Characteristic	Olanzapine (*n* = 53) ^1,2^	Clozapine (*n* = 24) ^2^
Gender (*n*, %)		
Female	15 (28.3)	6 (25)
Male	36 (67.9)	18 (75)
Unknown	2 (3.8)	0 (0.0)
Mean Age, y [95% CI]	35.5 ± 12.8 [95% CI: 32.0–39.0]	35.1 ± 8.4 [95% CI: 31.6–38.6]
Race (*n*, %) ^3,4^		
Caucasian	12 (22.6)	4 (16.7)
African Descent	15 (28.3)	9 (37.5)
Other	8 (15.0)	5 (20.8)
NS	18 (34.0)	6 (25.0)
Diagnosis (*n*, %)		
Schizophrenia	27 (50.9)	21 (87.5)
Bipolar Disorder	11 (20.8)	0 (0.0)
Other ^5^	15 (28.3)	2 (8.3)
NS	0 (0.0)	1 (4.2)
Median Time to Complication (in Months)	6 [IQR: 3–18] (*n* = 50)	2 [IQR: 1–4] (*n* = 23)
Dose (mg/day); [mean ± SD] ^6^	15.1 ± 9.1 [95% CI: 12.3–17.9] (*n* = 44)	310.3 ± 126.9 [95% CI: 247.2–373.4] (*n* = 18)
APD Discontinued (*n*, %)	39 (73.6)	16 (66.7)
Switching After DKA or HHS (*n*, %)	20 (37.7)	9 (37.5)
Recovery ^4^		
Full	16 (30.2)	4 (16.7)
Partial	28 (52.8)	17 (70.8)
Fatal	4 (7.5)	1 (4.2)
NS/U	5 (9.4)	2 (8.3)

Abbreviations: APD, antipsychotic drug; DKA, diabetic ketoacidosis; IQR, interquartile range; NS, not specified; SD, standard deviation; U, unsure; y, years. ^1^ Includes one CR where Risperidone was also administered. ^2^ Proportions are based on the total count for each SGA. ^3^ Other races include Aboriginal, Arab, Asian, Hispanic, Italian and mixed race. ^4^ African Descent includes African, African American, African Canadian, Afro-Caribbean and Black. ^5^ Other diagnoses include psychosis, depression, mood disorders, other diagnoses, and two or more diagnoses. ^6^ Mean dose is based on the highest dose received prior to complication if the dosage was titrated.

## Data Availability

No new data were generated or analyzed in this study. All data used were obtained from publicly available case reports.

## Supplementary Materials

The following supporting information can be downloaded at https://www.mdpi.com/article/10.3390/jcm15083107/s1. Supplemental Material I—PRISMA checklist (Table S1); Supplemental Material II: Search Strategies—Updated up to 17 October 2025; Supplemental Material III: Level 1 Title and Abstract Screening; Supplemental Material IV: Critical Appraisal Checklist and Explanations for Case Reports; Supplemental Material V—Additional detailed information for each case report (Tables S2 and S3) and critical appraisal for each case report (Table S4).
