# Supplementary material for: Antipsychotic Drugs and the Risk of Diabetic Complications: A Systematic Review of Clinical Evidence
Source: J Clin Med. 2026 Apr 18;15(8):3107. doi: 10.3390/jcm15083107 (PMC13116593; doi:10.3390/jcm15083107)
Supplement: Supplementary file 1 [file jcm-15-03107-s001.zip › jcm-4255350-supplementary.pdf]

Antipsychotic Drugs and the Risk of Diabetic Complications:

A Systematic Review of Clinical Evidence

Nisrine Haddad[1], Nawal Farhat[1,2], Christopher A. Gravel[1,3,4], Yue chen[1], Franco Momoli[1,5], Donald Mattison[1,5,6], Jeannette Goguen[7,8], Daniel Krewski[1,2,5]

[1]School of Epidemiology and Public Health, University of Ottawa, Ottawa, ON, Canada
[2]School of Mathematics and Statistics, Carleton University, Ottawa, Canada
[3]Department of Mathematics and Statistics, University of Ottawa, Ottawa, ON, Canada
[4]Data Literacy Research Institute, University of Ottawa, Ottawa, ON, Canada
[5]Risk Sciences International, Ottawa Canada
[6]Arnold School of Public Health, University of South Carolina, Columbia, South Carolina, United States
[7]Department of Medicine, University of Toronto
[8]Division of Endocrinology, St. Michael's Hospital

SUPPLEMENTAL MATERIAL

The following supplemental material provides the PRISMA checklist (Supplemental Material I-Table S1), the search strategy using four bibliographic databases (Supplemental Material II), Distiller SR form for Level 1 screening (Supplemental Material III), the Joanna Briggs Criteria for Assessment of Case Reports (Supplemental Material IV). Supplemental Material V presents additional detailed information for each case report (CR) is presented in (Tables S2 and S3) and critical appraisal for each CR (Table S4).

Supplemental Material I: PRISMA Checklist (Table S1)

Table S1. Preferred Reporting Items for Systematic reviews and Meta-Analyses extension for Scoping Reviews (PRISMA-ScR) Checklist.

SECTION	ITEM	PRISMA-ScR CHECKLIST ITEM	REPORTED ON PAGE #
**TITLE**			
Title	1	Antipsychotic Drugs and the Risk of Diabetic Complications: A Systematic Review of Clinical Evidence	1
**ABSTRACT**			
Structured summary	2	Provide a structured summary that includes (as applicable): background, objectives, eligibility criteria, sources of evidence, charting methods, results, and conclusions that relate to the review questions and objectives.	1
**INTRODUCTION**			

SECTION	ITEM	PRISMA-ScR CHECKLIST ITEM	REPORTED ON PAGE #
Rationale	3	Describe the rationale for the review in the context of what is already known. Explain why the review questions/objectives lend themselves to a scoping review approach.<br><br>Recent epidemiological and clinical evidence has suggested an association between the use of second generation antipsychotics (SGAs), also known as atypical antipsychotic drugs (APDs) and diabetic complications, namely diabetic ketoacidosis (DKA) and hyperglycemic hyperosmolar state (HHS). This systematic review analyses information abstracted from case reports and case series to provide an in-depth review of these adverse events associated with APD use, both first generation and second generation (typical and atypical), and to synthesize currently available evidence on the etiology of the two conditions.	2-5
Objectives	4	Provide an explicit statement of the questions and objectives being addressed with reference to their key elements (e.g., population or participants, concepts, and context) or other relevant key elements used to conceptualize the review questions and/or objectives.<br><br>To conduct a systematic review of evidence established in case reports (CRs) on adverse drug reactions, specifically diabetic ketoacidosis (DKA) and hyperglycemic hyperosmolar state (HHS), associated with the use of first and second generation antipsychotics (FGAs and SGAs).	2-5
**METHODS**			
Protocol and registration	5	Indicate whether a review protocol exists; state if and where it can be accessed (e.g., a Web address); and if available, provide registration information, including the registration number.<br><br>N/A	
Eligibility criteria	6	Specify characteristics of the sources of evidence used as eligibility criteria (e.g., years considered, language, and publication status), and provide a rationale.<br><br>Case reports/case series spanned all years up to 2025.<br>Both English and French abstracts were considered.<br>There was no restriction to geographic location.<br>Case reports with antipsychotic drug monotherapy or concomitant use of FDA approved antipsychotic drugs were included.<br>Outcomes of interest: diabetic ketoacidosis (DKA) and hyperglycemic hyperosmolar state (HHS) or both.<br>Additional information can be found in the Methods section.	6
Information sources*	7	Describe all information sources in the search (e.g., databases with dates of coverage and contact with authors to identify additional sources), as well as the date the most recent search was executed.	5

SECTION	ITEM	PRISMA-ScR CHECKLIST ITEM	REPORTED ON PAGE #
		Bibliographic databases (MEDLINE, EMBASE, PsycInfo, and the Cochrane Central Register of Controlled Trials (CENTRAL)) were searched using index phrases and key words through to October 17th, 2025.	
Search	8	Present the full electronic search strategy for at least 1 database, including any limits used, such that it could be repeated.<br><br>Please refer to Supplemental material II – Search strategies are shown for each database.	7-14
Selection of sources of evidence†	9	State the process for selecting sources of evidence (i.e., screening and eligibility) included in the scoping review.<br><br>Qualitative and quantitative data was abstracted from each case report. Relevant quantitative and qualitative data from all CRs included in the review were abstracted, including demographic characteristics (e.g., gender, age, ethnicity), clinical information (e.g., diagnosis, treatment, history of diabetes, comorbidities, co-medications, and prognosis, signs and symptoms), and quantitative and qualitative data on relevant biomarkers such as levels of glucose or HbA1c, body weight change, presence or absence of ketones.	5-7
Data charting process‡	10	Describe the methods of charting data from the included sources of evidence (e.g., calibrated forms or forms that have been tested by the team before their use, and whether data charting was done independently or in duplicate) and any processes for obtaining and confirming data from investigators.<br><br>First-level screening was conducted independently by two reviewers using DistillerSR to manage and track records. Second-level screening was performed using Excel spreadsheets. Data extraction was completed by one of the two reviewers, with duplicate data abstraction conducted on 10% of the included studies by the second reviewer as a quality control measure. Summary tables were developed to summarize key findings. No calibrated forms were used, and data were not formally confirmed with original investigators.<br><br>Please refer to L1 screening form in Supplemental Material.	5-7<br><br>Supplemental Page 15
Data items	11	List and define all variables for which data were sought and any assumptions and simplifications made.<br><br>**Variables and Assumptions:**<br>Data were collected on variables including:<br><br>• Demographics (e.g., age, sex, race)<br>• Clinical presentation (e.g., symptoms, diagnostic findings)<br>• Laboratory values (e.g., blood glucose, HbA1C, presence ketones)<br>• Diagnosis (e.g., schizophrenia, bipolar disorder, etc.)<br>• Treatment approach (e.g., insulin therapy, fluid replacement)	5,6

SECTION	ITEM	PRISMA-ScR CHECKLIST ITEM	REPORTED ON PAGE #
		• Medication use (e.g., antipsychotic agents)<br>• Outcome: diabetic ketoacidosis (DKA), hyperosmolar hyperglycemic state (HHS)<br>• Type of recovery (e.g., Full, partial, fatal)<br>**Assumptions and Simplifications:**<br>• Antipsychotic medication use was assumed to be single-agent unless antipsychotic polypharmacy was explicitly stated at the time of presentation.<br>• For quantitative laboratory data, the final test results reported were used for analysis, under the assumption that the most recent clinical values best represent the patient's status at follow-up.<br>• In cases of missing data, no imputation was performed; only available reported values were used.	
Critical appraisal of individual sources of evidence§	12	If done, provide a rationale for conducting a critical appraisal of included sources of evidence; describe the methods used and how this information was used in any data synthesis (if appropriate).<br><br>The Joanna Briggs Critical Appraisal tool for case reports was used.<br><br>Please refer to Supplemental Material.	7<br><br>Supplemental Material Pages 16-18
Synthesis of results	13	Describe the methods of handling and summarizing the data that were charted.<br><br>Data were synthesized using descriptive methods. Data from individual sources were organized and summarized in tables, with information on key variables abstracted from each case report. Key variables (e.g., diagnosis, history of diabetes, antipsychotic used, lab values, and outcomes). Quantitative data (e.g., glucose levels, HbA1C, presence of ketones) were summarized using simple averages.<br><br>Each included case report underwent a critical appraisal using the *Joanna Briggs Institute (JBI) Critical Appraisal Checklist for Case Reports* to assess methodological quality and identify potential sources of bias.<br><br>Appraisal results were considered during data synthesis but did not lead to exclusion of any studies.	5-7
**RESULTS**			
Selection of sources of evidence	14	Give numbers of sources of evidence screened, assessed for eligibility, and included in the review, with reasons for exclusions at each stage, ideally using a flow diagram.<br><br>Please refer to Figure 1 - Flow Chart for the Selection of Case Reports Included in the Systematic Review	8

SECTION	ITEM	PRISMA-ScR CHECKLIST ITEM	REPORTED ON PAGE #
Characteristics of sources of evidence	15	For each source of evidence, present characteristics for which data were charted and provide the citations.<br><br>Key characteristics for each included source of evidence were presented in summary tables. These included the citation, patient demographics (age, sex), clinical diagnosis (DKA, HHS, or both), antipsychotic medication used, laboratory findings, treatment, and patient outcomes. All relevant data were extracted directly from the reports and are fully cited in the tables.<br><br>Please refer to Tables 3 to 8 of the Results section / Supplementary Material V, Tables S2, S3 and S4.	9-16; 19-20<br><br>Supplemental Material: 20-37
Critical appraisal within sources of evidence	16	If done, present data on critical appraisal of included sources of evidence (see item 12).<br><br>Please refer to:<br><br>• Figure 2<br>• Supplemental Material V-Table S4	21<br><br>Supplemental Material: 38-46
Results of individual sources of evidence	17	For each included source of evidence, present the relevant data that were charted that relate to the review questions and objectives.<br><br>For each included source, relevant data that directly address the objectives of this review were abstracted and presented in detailed summary tables. These data include the type of antipsychotic medication used, associated outcomes (DKA, HHS, or both), laboratory values, clinical presentation, management strategies, and patient outcomes.<br><br>Data for each individual case report are available in Tables 3 to 8 of the Results section<br><br>Supplementary Material V, Tables S2, S3, and S4 with corresponding citations.	9-16; 19-21<br><br>Supplemental Material: 20-46
Synthesis of results	18	Summarize and/or present the charting results as they relate to the review questions and objectives.<br><br>Data for each individual case report are available in Tables 3 to 8/ the Results section<br><br>Supplementary Material V, Tables S2, S3, and S4 with corresponding citations.	7-21<br><br>Supplemental Material: 20-46
**DISCUSSION**			
Summary of evidence	19	Summarize the main results (including an overview of concepts, themes, and types of evidence available), link to the review questions and objectives, and consider the relevance to key groups.<br><br>Please refer to the Discussion and Conclusion	21-26

SECTION	ITEM	PRISMA-ScR CHECKLIST ITEM	REPORTED ON PAGE #
Limitations	20	Discuss the limitations of the scoping review process.<br><br>The case reports (CRs) varied in detail and completeness, and sometimes this made it challenging to construct precise timelines and gather consistent historical information. Data gaps existed in patient history, quantitative measures, and condition management. Despite these limitations, the CRs offer valuable insights into the etiology of diabetic ketoacidosis (DKA) and HHS, and a better understanding of the association with antipsychotic drug use and these diabetic complications.	25
Conclusions	21	Provide a general interpretation of the results with respect to the review questions and objectives, as well as potential implications and/or next steps.<br><br>Analysis provides additional evidence of an association between the use of atypical antipsychotic drugs, and diabetic ketoacidosis and hyperglycemic hyperosmolar state.<br><br>Additional research would be beneficial to elucidate the mechanisms of action of antipsychotic drugs, as well as to ensure effective and safe use of these medicines in patients who are susceptible to developing conditions associated with the metabolic syndrome.	25
**FUNDING**			
Funding	22	Describe sources of funding for the included sources of evidence, as well as sources of funding for the scoping review. Describe the role of the funders of the scoping review.<br><br>N/A	

Supplemental Material II: Search Strategies-Updated up to 17 October 2025

MEDLINE

1 Aripiprazole/ 3154

2 (abilify or Aripiprazole or asenapine maleate or saphris or clozaril or clozapine or iloperidone or fanapt or lurasidone or latuda or olanzapine* or zyprexa or symbyax or paliperidone or invega or quetiapine or seroquel or risperidone or risperdal or ziprasidone or geodon or cariprazine or Vraylar or Pimavanserin or Nuplazid).ti,ab,kw. 37361

3 clozapine/ 9937

4 Lurasidone Hydrochloride/ 435

5 Paliperidone Palmitate/ 1139

6 Quetiapine Fumarate/ 3354

7 Risperidone/ 7056

8 Pimavanserin/ 0

9 cariprazine/ 0

10 1 or 2 or 3 or 4 or 5 or 6 or 7 or 8 or 9 39801

11 diabetes complications/ or diabetic coma/ or hyperglycemic hyperosmolar nonketotic coma/ or diabetic ketoacidosis/ 55044

12 (diabet* adj3 complication*).ti,ab,kw. 43885

13 hyperglycemi*.ti,ab,kw. 70426

14 (diabet* adj3 ketoacidosis).ti,ab,kw. 9401

15 (diabet* adj3 (coma* or hyperglycemi*)).ti,ab,kw.10002

16 11 or 12 or 13 or 14 or 15158292

17 10 and 16 398

18 HALOPERIDOL/ 16274

19 (Haloperidol or Haldol or Loxapine or Loxitane or Molindone or Moban or Thiothixene or Navane or Primozide or Orap or Trifluoperazine or Stelazine or Chlorpromazine or Thorazine or Fluphenazine or Perphenazine or "Perphenazine amitriptyline" or Prochlorperazine or Thioridazine or Droperidol or Inapsine).ti,ab,kw. 43221

20	CHLORPROMAZINE/	17477
21	FLUPHENAZINE/	2441
22	Loxapine/	327
23	PERPHENAZINE/	1599
24	Prochlorperazine/	1101
25	PROCHLORPERAZINE/	1101
26	THIORIDAZINE/	2406
27	TRIFLUOPERAZINE/	3636
28	THIOTHIXENE/	335
29	Thioridazine/	2406
30	Droperidol/	2036
31	LOXAPINE/	327
32	MOLINDONE/	145
33	PIMOZIDE/	1757
34	Fluphenazine/	2441
35	18 or 19 or 20 or 21 or 22 or 23 or 24 or 25 or 26 or 27 or 28 or 29 or 30 or 31 or 32 or 33 or 34	58621
36	16 and 35	159
37	17 or 36	515
38	10 or 35	91913
39	16 and 38	515
40	limit 39 to ed=20200901-20251015	62
41	limit 39 to dt=20200901-20251015	83
42	40 or 41	91

CENTRAL

1 Aripiprazole/ 743

2 (abilify or Aripiprazole or asenapine maleate or saphris or clozaril or clozapine or iloperidone or fanapt or lurasidone or latuda or olanzapine* or zyprexa or symbyax or paliperidone or invega or quetiapine or seroquel or risperidone or risperdal or ziprasidone or geodon or cariprazine or Vraylar or Pimavanserin or Nuplazid).ti,ab,kw. 10907

3 clozapine/ 606

4 Lurasidone Hydrochloride/ 153

5 Paliperidone Palmitate/ 316

6 Quetiapine Fumarate/ 849

7 Risperidone/ 1614

8 Pimavanserin/ 9

9 cariprazine/ 6

10 1 or 2 or 3 or 4 or 5 or 6 or 7 or 8 or 9 11121

11 diabetes complications/ or diabetic coma/ or hyperglycemic hyperosmolar nonketotic coma/ or diabetic ketoacidosis/ 1802

12 (diabet* adj3 complication*).ti,ab,kw. 5739

13 hyperglycemi*.ti,ab,kw. 8890

14 (diabet* adj3 ketoacidosis).ti,ab,kw. 992

15 (diabet* adj3 (coma* or hyperglycemi*)).ti,ab,kw.663

16 11 or 12 or 13 or 14 or 1516261

17 10 and 16 68

18 HALOPERIDOL/ 1576

19 (Haloperidol or Haldol or Loxapine or Loxitane or Molindone or Moban or Thiothixene or Navane or Primozide or Orap or Trifluoperazine or Stelazine or Chlorpromazine or Thorazine or Fluphenazine or Perphenazine or "Perphenazine amitriptyline" or Prochlorperazine or Thioridazine or Droperidol or Inapsine).ti,ab,kw. 6001

20 CHLORPROMAZINE/ 653

21	FLUPHENAZINE/	302
22	Loxapine/	80
23	PERPHENAZINE/	215
24	Prochlorperazine/	216
25	PROCHLORPERAZINE/	216
26	THIORIDAZINE/	200
27	TRIFLUOPERAZINE/	122
28	THIOTHIXENE/	74
29	Thioridazine/	200
30	Droperidol/	522
31	LOXAPINE/	80
32	MOLINDONE/	23
33	PIMOZIDE/	123
34	Fluphenazine/	302
35	18 or 19 or 20 or 21 or 22 or 23 or 24 or 25 or 26 or 27 or 28 or 29 or 30 or 31 or 32 or 33 or 34	6681
36	16 and 35	15
37	17 or 36	76
38	10 or 35	16110
39	16 and 38	76
40	limit 39 to yr="2020-current"	18

EMBASE

1 Aripiprazole/ 23479

2 (abilify or Aripiprazole or asenapine maleate or saphris or clozaril or clozapine or iloperidone or fanapt or lurasidone or latuda or olanzapine* or zyprexa or symbyax or paliperidone or invega or quetiapine or seroquel or risperidone or risperdal or ziprasidone or geodon or cariprazine or Vraylar or Pimavanserin or Nuplazid).ti,ab,kw. 61127

3 clozapine/ 40526

4 Lurasidone Hydrochloride/ 3131

5 Paliperidone Palmitate/ 7448

6 Quetiapine Fumarate/ 32505

7 Risperidone/ 47067

8 Pimavanserin/ 1077

9 cariprazine/ 1478

10 1 or 2 or 3 or 4 or 5 or 6 or 7 or 8 or 9 107324

11 diabetes complications/ or diabetic coma/ or hyperglycemic hyperosmolar nonketotic coma/ or diabetic ketoacidosis/ 34756

12 (diabet* adj3 complication*).ti,ab,kw. 65822

13 hyperglycemi*.ti,ab,kw. 107838

14 (diabet* adj3 ketoacidosis).ti,ab,kw. 16083

15 (diabet* adj3 (coma* or hyperglycemi*)).ti,ab,kw.14086

16 11 or 12 or 13 or 14 or 15189539

17 10 and 16 1074

18 HALOPERIDOL/ 64903

19 (Haloperidol or Haldol or Loxapine or Loxitane or Molindone or Moban or Thiothixene or Navane or Primozide or Orap or Trifluoperazine or Stelazine or Chlorpromazine or Thorazine or Fluphenazine or Perphenazine or "Perphenazine amitriptyline" or Prochlorperazine or Thioridazine or Droperidol or Inapsine).ti,ab,kw. 47553

20 CHLORPROMAZINE/ 43669

21	FLUPHENAZINE/	10115
22	Loxapine/	2864
23	PERPHENAZINE/	7577
24	Prochlorperazine/	6720
25	PROCHLORPERAZINE/	6720
26	THIORIDAZINE/	12383
27	TRIFLUOPERAZINE/	10351
28	THIOTHIXENE/	2663
29	Thioridazine/	12383
30	Droperidol/	10025
31	LOXAPINE/	2864
32	MOLINDONE/	1300
33	PIMOZIDE/	8699
34	Fluphenazine/	10115
35	18 or 19 or 20 or 21 or 22 or 23 or 24 or 25 or 26 or 27 or 28 or 29 or 30 or 31 or 32 or 33 or 34	129700
36	16 and 35	719
37	17 or 36	1447
38	10 or 35	204295
39	16 and 38	1447
40	limit 39 to yr="2020 -Current"	354
41	limit 39 to dc=20200901-20251015	380
42	limit 39 to dd=20200901-20251015	381
43	41 or 42	383

PsycINFO

1 Aripiprazole/ 2079

2 (abilify or Aripiprazole or asenapine maleate or saphris or clozaril or clozapine or iloperidone or fanapt or lurasidone or latuda or olanzapine* or zyprexa or symbyax or paliperidone or invega or quetiapine or seroquel or risperidone or risperdal or ziprasidone or geodon or cariprazine or Vraylar or Pimavanserin or Nuplazid).ti,ab,mp. 24625

3 clozapine/ 5847

4 Lurasidone Hydrochloride/ 0

5 Paliperidone Palmitate/ 0

6 Quetiapine Fumarate/ 0

7 Risperidone/ 4102

8 Pimavanserin/ 0

9 cariprazine/ 0

10 1 or 2 or 3 or 4 or 5 or 6 or 7 or 8 or 9 24625

11 diabetes complications/ or diabetic coma/ or hyperglycemic hyperosmolar nonketotic coma/ or diabetic ketoacidosis/ 0

12 (diabet* adj3 complication*).ti,ab,mp. 2219

13 hyperglycemi*.ti,ab,mp. 2132

14 (diabet* adj3 ketoacidosis).ti,ab,mp. 210

15 (diabet* adj3 (coma* or hyperglycemi*)).ti,ab,mp. 208

16 11 or 12 or 13 or 14 or 154359

17 10 and 16 231

18 HALOPERIDOL/ 4868

19 (Haloperidol or Haldol or Loxapine or Loxitane or Molindone or Moban or Thiothixene or Navane or Primozide or Orap or Trifluoperazine or Stelazine or Chlorpromazine or Thorazine or Fluphenazine or Perphenazine or "Perphenazine amitriptyline" or Prochlorperazine or Thioridazine or Droperidol or Inapsine).ti,ab,mp. 16702

20 CHLORPROMAZINE/ 1763

21 FLUPHENAZINE/ 550

22	Loxapine/	93
23	PERPHENAZINE/	199
24	Prochlorperazine/	27
25	PROCHLORPERAZINE/	27
26	THIORIDAZINE/	373
27	TRIFLUOPERAZINE/	146
28	THIOTHIXENE/	104
29	Thioridazine/	373
30	Droperidol/	0
31	LOXAPINE/	93
32	MOLINDONE/	48
33	PIMOZIDE/	491
34	Fluphenazine/	550
35	18 or 19 or 20 or 21 or 22 or 23 or 24 or 25 or 26 or 27 or 28 or 29 or 30 or 31 or 32 or 33 or 34	17065
36	16 and 35	53
37	17 or 36	250
38	10 or 35	37280
39	16 and 38	250
40	limit 39 to up=20200901-20251015	19

Supplemental Material III: Level 1 Title and Abstract Screening.

1. Should this study be included in Stage 2 screening?

- ◯ Yes
- ◯ Unsure
- ◯ No

2. Reason for exclusion:

- ☐ Population
- ☐ Interventions(s)/Exposure(s)
- ☐ Comparison
- ☐ Outcome(s)
- ☐ Study Design

Supplemental Material IV: Critical Appraisal Checklist and Explanations for Case Reports.

The Joanna Briggs Institute

Introduction

The Joanna Briggs Institute (JBI) is an international, membership-based research and development organization within the Faculty of Health Sciences at the University of Adelaide. The Institute specializes in promoting and supporting evidence-based healthcare by providing access to resources for professionals in nursing, midwifery, medicine, and allied health. With over 80 collaborating centres and entities, servicing over 90 countries, the Institute is a recognized global leader in evidence-based healthcare.

JBI Systematic Reviews

The core of evidence synthesis is the systematic review of literature of a particular intervention, condition or issue. The systematic review is essentially an analysis of the available literature (that is, evidence) and a judgment of the effectiveness or otherwise of a practice, involving a series of complex steps. The JBI takes a particular view on what counts as evidence and the methods utilized to synthesize those different types of evidence. In line with this broader view of evidence, the Institute has developed theories, methodologies and rigorous processes for the critical appraisal and synthesis of these diverse forms of evidence in order to aid in clinical decision-making in health care. There now exists JBI guidance for conducting reviews of effectiveness research, qualitative research, prevalence/incidence, etiology/risk, economic evaluations, text/opinion, diagnostic test accuracy, mixed-methods, umbrella reviews and scoping reviews. Further information regarding JBI systematic reviews can be found in the JBI Reviewer's Manual on our website.

JBI Critical Appraisal Tools

All systematic reviews incorporate a process of critique or appraisal of the research evidence. The purpose of this appraisal is to assess the methodological quality of a study and to determine the extent to which a study has addressed the possibility of bias in its design, conduct and analysis. All papers selected for inclusion in the systematic review (that is – those that meet the inclusion criteria described in the protocol) need to be subjected to rigorous appraisal by two critical appraisers. The results of this appraisal can then be used to inform synthesis and interpretation of the results of the study. JBI Critical appraisal tools have been developed by the JBI and collaborators and approved by the JBI Scientific Committee following extensive peer review. Although designed for use in systematic reviews, JBI critical appraisal tools can also be used when creating Critically Appraised Topics (CAT), in journal clubs and as an educational tool.

JBI Critical Appraisal Checklist for Case Reports

Reviewer _____________________________________ Date ___________________________

Author _____________________________________ Year _________ Record Number

	Yes	No	Unclear	Not applicable
1. Were patient's demographic characteristics clearly described?	□	□	□	□
2. Was the patient's history clearly described and presented as a timeline?	□	□	□	□
3. Was the current clinical condition of the patient on presentation clearly described?	□	□	□	□
4. Were diagnostic tests or assessment methods and the results clearly described?	□	□	□	□
5. Was the intervention(s) or treatment procedure(s) clearly described?	□	□	□	□
6. Was the post-intervention clinical condition clearly described?	□	□	□	□
7. Were adverse events (harms) or unanticipated events identified and described?	□	□	□	□
8. Does the case report provide takeaway lessons?	□	□	□	□

Overall appraisal: Include □ Exclude □ Seek further info □

Comments (Including reason for exclusion)

____

____

____

Explanation of case reports critical appraisal

Moola S, Munn Z, Tufanaru C, Aromataris E, Sears K, Sfetcu R, Currie M, Lisy K, Qureshi R, Mattis P, Mu P. Chapter 7: Systematic reviews of etiology and risk. In: Aromataris E, Munn Z (Editors). *JBI Manual for Evidence Synthesis.* JBI, 2020. Available from https://synthesismanual.jbi.global. https://doi.org/10.46658/JBIMES-20-08

Case Reports Critical Appraisal Tool

Answers: Yes, No, Unclear or Not/Applicable

1. **Were patient's demographic characteristics clearly described?**

Does the case report clearly describe patient's age, sex, race, medical history, diagnosis, prognosis, previous treatments, past and current diagnostic test results, and medications? The setting and context may also be described.

2. **Was the patient's history clearly described and presented as a timeline?**

A good case report will clearly describe the history of the patient, their medical, family and psychosocial history including relevant genetic information, as well as relevant past interventions and their outcomes. (CARE Checklist 2013)

3. **Was the current clinical condition of the patient on presentation clearly described?**

The current clinical condition of the patient should be described in detail including the uniqueness of the condition/disease, symptoms, frequency, and severity. The case report should also be able to present whether differential diagnoses was considered.

4. **Were diagnostic tests or methods and the results clearly described?**

A reader of the case report should be provided sufficient information to understand how the patient was assessed. It is important that all appropriate tests are ordered to confirm a diagnosis and therefore the case report should provide a clear description of various diagnostic tests used (whether a gold standard or alternative diagnostic tests). Photographs or illustrations of diagnostic procedures, radiographs, or treatment procedures are usually presented when appropriate to convey a clear message to readers.

5. **Was the intervention(s) or treatment procedure(s) clearly described?**

It is important to clearly describe treatment or intervention procedures as other clinicians will be reading the paper and therefore may enable clear understanding of the treatment protocol. The report should describe the treatment/intervention protocol in detail; for e.g. in pharmacological management of dental anxiety - the type of drug, route of administration, drug dosage and frequency, and any side effects.

6. **Was the post-intervention clinical condition clearly described?**

A good case report should clearly describe the clinical condition post-intervention in terms of the presence or lack thereof symptoms. The outcomes of management/treatment when presented as images or figures would help in conveying the information to the reader/clinician.

7. Were adverse events (harms) or unanticipated events identified and described?

With any treatment/intervention/drug, there are bound to be some adverse events and in some cases, they may be severe. It is important that adverse events are clearly documented and described, particularly when a new or unique condition is being treated or when a new drug or treatment is used. In addition, unanticipated events, if any that may yield new or useful information should be identified and clearly described.

8. Does the case report provide takeaway lessons?

Case reports should summarize key lessons learned from a case in terms of the background of the condition/disease and clinical practice guidance for clinicians when presented with similar cases.

References:

Gagnier, J. J., Kienle, G., Altman, D. G., Moher, D., Sox, H., Riley, D., & CARE Group (2013). The CARE guidelines: consensus-based clinical case reporting guideline development. *Headache*, *53*(10), 1541–1547. https://doi.org/10.1111/head.12246

Supplemental Material V Additional detailed information for each case report (Tables S2 and S3) and critical appraisal for each case report (Table S4)

Table S2. Summary of the characteristics of patients diagnosed with diabetic ketoacidosis associated with antipsychotic drug use (n = 123)[1-7].

Pt No	Citation	Clinical signs and symptoms	Type of APD Therapy	APD switch after DKA	DM Diagnosis before/after complication	History of DM	Antidiabetic before / after DKA event [1]	Time to Complication (estimates when not stated)	APD Discontinued[5]	Overall Weight Changes (BMI kg/m², Qualitative)	BG (Qualitative):<br>•Before complication<br>•At presentation<br>•After resolution	Plasma/Serum Glucose (Quantitative, mg/dl)[6]<br>•Before complication<br>•At presentation<br>•After resolution	HbA1c (%)<br>•Before complication<br>•At presentation<br>•After resolution[1]	Ketones +	Treatment of DKA with insulin	Insulin treatment (Last recorded insulin dosage if specified)	Treatment with insulin or antidiabetic required after resolution	Was patient followed up?	Recovery after resolution	Co-morbidities[7]	Concomitant Medication (other than APDs)[2]	Other known issues
1	Adhoni et al. (2021) [57]	Abdominal pain, vomiting, loss of appetite, tachy cardic, acanthosis nigricans of the neck, blood pressure	MT	Y, arip.	N/ Y,T2DM	Family history of DM	After (temp.)	21 months	Y	I (39.6/(OB)	NS<br>Elevated<br>Controlled	NS<br>575<br>NS	NS<br>12.5<br>5.7	Y	Y	•Insulin glargine, 10 units	Y, insulin and metformin temp.	Y	Partial (diet. mgt/exercise)	N	N	NS
2	Agrawal et al. (2016) [58]	Generliazed weakness, abdominal discomfort, polyuria, polydipsia, weight loss	MT	Y, risp.	N / New onset DKA	No patient history	N	3 months	Y	D	NS<br>NS<br>Euglycemic	NS<br>896 (st)<br>NS	NS<br>NS<br>NS	Y	Y	•Insulin and fluid resuscitation (70 units, twice daily insulin glargine, 10 units 3 times a day, regular insulin)	N, off insulin within 6 months	Y	Full	NS	NS	NS
3	Ai et al. (1998) [59]	Severe vomiting	MT	Y, olanz	N/ Y, T2DM	No patient<br>No family history	After	5 months	Y	NS	NS<br>NS<br>Controlled	NS<br>448.2<br>221.9 (st)	NS<br>11<br>NS	Y	Y	•Insulin and fluid<br>•Sliding scale insulin, IV rehydration, and potassium supplements	Y, hypoglycaemic agent (gliclazide)	Y	Partial	•Mild Asthma<br>•Hidradenitis suppurativa	Minocycline	N
4	Akunjee et al. (2018) [60]	Fatigue, polyuria, polydipsia, confusion	PP (Risp.)	N	Y / Y (prediabetic)	Patient history (pre-diabetes)	N	9 years	Y, olanz.	I	Prediabetic<br>Improved<br>Prediabetic	NS<br>NS<br>NS	6<br>13.7<br>5.8	NS	Y	•IV insulin, dialysis and mechanical ventilation.<br>•Discharged on glargine insulin	N	Y	Full	NS	N	NS
5	Al-Amri (2009) [61]	Progressive somnolence, polyuria, polydipsia	MT	Y, halop.	N / New onset DM	No patient<br>No family history	After	2 months	Y	I (33.3/(OB)	Normal<br>NS<br>Norm.	NS<br>800<br>NS	NS<br>NS<br>NS	Y	Y	•Insulin, metformin and and IV fluids	Y, insulin	Y	Partial	NS	Clonazepam valproate	NS
6	Alex et al. (2018) [62]	Vomiting, headache, fever	MT	Y, arip.	N / Y,T2DM	Family history	After	3 months	Y	SM	NS<br>NS<br>NS	111.9<br>378<br>69.7	NS<br>14<br>NS	Y	Y	•Insulin and IV potassium infusions	Y, insulin, metformin	Y	Partial, (diet mgt.)	NS	NS	NS
7	Almahmood et al. (2025) [63][1]	Unresponsive to sound or touch, decreased level of consciousness, tachypnea, tachycardia, febrile, rigidity in th limbs, best response on the Glasgow Coma Scale (GCS) was 6/15	MT	N	N / NS	No patient history	N	3 months	Y	NS (22.1)	NS<br>NS<br>NS	91.8<br>599.4<br>288	5.2<br>8.2<br>NS	Y	Y	•IV fluids, insulin	N	Y	Full	NS	•Mitrazapine,<br>•Memantine<br>•Lorazepam	N

8	Ananth et al. (2004) [64]	Seizure, coma, somnolence, high fever	MT	N	N/ N	No patient history<br>No family history	NS	2 years	Y	NS	NS<br>NS<br>Norm.	NS<br>1201<br>NS	NS<br>NS<br>NS	NS	Y	•Insulin<br>NS<br>•Fluids, soluble insulin (20 units/day, then stopped due to hypogl.)	N	Y	Full	NS	Lithium carbonate	NS
9	Aruna and Paulose (1995) [65]	Thirst, dryness of mouth, weight loss, fatigue	PP (Halop.)	N	N /<br>Y, T1DM	Family history	N	1 month	Y	NS	NS<br>U(+)<br>Normal	NS<br>NS<br>119	NS<br>4.2<br>NS	Y	Y	Insulin	N	Y	Full	N	Carbamazepine	NS
10	Atabay and Rodopman Arman (2019) [66]	Nausea, vomiting, abdominal pain and blurred consciousness	PP (Arip.)	Y, arip.	N / Y	Family history	N	2 months	Y	NS	NS<br>3+ (Urine)<br>NS	NS<br>486.1 (st)<br>NS	NS<br>NS<br>NS	Y	Y	Insulin	Insulin	Y	Partial	NS	NS	N
11	Avella et al. (2004) - Case 1 [67]	Tutti-frutti odor, olanzapine in the liver	MT	N/A	N / N/A	No patient history	N	3 years	N/A	NS (OB)	NS<br>NS<br>NA	NS<br>N/A<br>N/A	NS<br>NS<br>NA	Y	N/A	N/A	N/A	N/A	Fatal	•Bronchitis<br>•Herniated lumbar disc<br>•Right lateral strabismus	•Albuterol<br>•Clonazepam<br>•Inderal<br>•Neurontin<br>•Celebrex<br>•Topirimate	Tobacco use
12	Avella et al. (2004) - Case 2 [67]	N/A	MT	N/A	N / N/A	No patient history	N	2.5 years	N/A	NS (OB)	NS<br>NS<br>NA	NS<br>N/A<br>NA	NS<br>14.2 (post-mortem)<br>N/A	Y	N/A	N/A	N/A	N/A	Fatal	NS	•Fluoxetine	Tobacco use
13	Avella et al. (2004) - Case 3 [67]	N/A	MT	N/A	N / N/A	No patient history	N	4-5 months	N/A	NS	NS<br>NS<br>NA	NS<br>N/A<br>N/A	NS<br>14.7 (post-mortem)<br>N/A	Y	N/A	N/A	N/A	N/A	Fatal	N	N	NS
14	Avram et al. (2001) [68]	Nausea, vomiting, dehydration, abdominal pain	MT	N	N / N	No patient history<br>No family history	N	8 months	Y	D (32.5/OW)	NS<br>Present<br>Normal.	NS<br>626<br>83	NS<br>14<br>NS	Y	Y	•IV insulin and hydration, electrolyte replacement (40 U morning; 25 units evening)	N	Y	Full	N	•Sertraline,<br>•Ranitidine,<br>•Trihexyphenidyl	NS
15	Bae et al. (2024) [69]	Dyspnea, auditory hallucinations, persecutory delusions, and depressive feelings	PP	Y, arip (temp.)	N/ N	No patient history	After (temp.)	NS	Y (both)	D (39.6/OB)	NS<br>NS<br>Improved	194.9 (st)<br>1086.4 (st)<br>145.6 (st)	5.7<br>14.4<br>6.1	Y	Y	•Fluid therapy, intravenous insulin administration, and electrolyte correction	Y, insulin, oral hypoglycemics: Metformin, vildaqliptin, limepiride, temp	Y	Full	N	•Lithium,<br>•Lorazepam	Drug overdose
16	Buch et al. (2003) [70]	Nausea, vomiting, polyuria, polydipsia, weight loss,, dehydration, lethargy and apyrexial	MT	Y, flup.	N / N	No patient history<br>No family history	N	3 months	Y	D	NS<br>NS<br>Norm.	NS<br>720<br>NS	NS<br>NS<br>NS	Y	Y	•Insulin and IV fluids, (6 U/H)<br>•Human Mixtard 30/70, 20 units morning and 20 units evening	N	Y	Full	N	NS	NS
17	Cabrera et al. (2021) [71]	Altered mental status, obtunded, unable to follow commands, lack of response to painful stimuli, tachycardia, tachypnea, blood pressure of hypoxemia	PP, (Cloz.)	NS	N / DM	NS	NS	NS	Y, (both)	NS, OB	NS<br>NS<br>NS	NS<br>1700<br>NS	NS<br>NS<br>NS	Y	NS	NS	NS	NS	NS	NS	•Lithium<br>•Carbonate<br>•Benztropine<br>•Trazodone	NS

18	Cardinale et al. (2019) [72]	Acute respiratory failure	MT	NS	NS/<br>NS	NS	NS	24 months	NS	NS	NS<br>NS<br>NS	NS<br>720<br>NS	NS<br>NS<br>NS	NS	NS	•Lipid lowering rosuvastatin, insulin therapy, continuous renal replacement therapy	NS	U	NS	NS	NS	N
19	Chellamuthu et al. (2010) [73]	Reduced conscious level, Diarrhoea, vomiting	MT	Y, halop.	N / T2DM	No patient history<br>No family history	After	NS	Y	SM (27/OB)	NS<br>NS<br>NS	NS<br>1679.4<br>NS	NS<br>13.8<br>7.2	NS	Y	•Fluid replacement and insulin (84 units)	Y,insulin, metformin	Y	Partial	NS	NS	NS
20	Cho & Lindenmayer (2009) [74]	Epigastric pain, emesis, dizziness, lethargy.	PP<br>(Halop.)	Y, arip (discontinued); molindone, halop.	N / New onset DM	No Patient history	N	16 months	Y<br>(Cloz.)	NS (OB)	Normal<br>NS<br>NS	NS<br>448 (st)<br>NS	NS<br>NS<br>NS	Y	Y	NS	NS	Y	Partial	•Asthma<br>•Dyslipidemia	•Valproic acid•Nortriptyline<br>•Albuterol inhaler<br>•Ibuprofen	•Substance abuse<br>•Cocaine<br>•Alcoholism
21	Church et al. (2005) [75]	Nausea, vomiting, malaise	PP<br>(Olanz.)	N	Y, T2DM	Patient history	Before	4 days	Y	NS	NS<br>NS<br>NS	NS,<br>577.8<br>241.07	NS<br>NS<br>NS	Y	Y	•IV insulin, fluid replacements (NPH: 70/30 insulin 36 U morning; 20 U evening)	Y	Y	Partial	NS	•Metformin,<br>•Rosiglitazone<br>•Diazepam	NS
22	Colli et al. (1999) [76]	Obtundation, dehydration	MT	N	N / N	No patient history<br>No family history	N	3 months	Y	I<br>(29/OW)	Evidence<br>NS<br>Stabilized	NS<br>756<br>92.9	NS<br>NS<br>5.9	Y	Y	•IV insulin, fluids.<br>(40 U)	N	Y	Partial (diet mgt.)	NS	N	N
23	Courvoisie et al. (2004) [77]	Polyuria, polydipsia, nocturia, decreased appetite, lethargy, abdominal pain	MT	N	N / New onset DM	Family history	NS	9 months (Check)	Y	I<br>(OB)	NS<br>U(+)<br>Stabilized	NS<br>302<br>140	NS<br>NS<br>8.3	Y	Y	•Insulin<br>(6 units NPH and 2 units regular morning, 3 units NPH, and 4 units regular evening)	N	Y	Partial	•Attention Deficit hyperactivity disorder; oppositional defiant disorder	•Stimulants,<br>•Guanfacine,<br>•Valproic acid<br>•Guanfacine<br>•Amphetamine	N
24	Croarkin et al. (2000)[2] [78]	Polyuria, polydipsia, daily emesis, abdominal pain. Delirious, reduced consciousness, disorientation	MT	Y, quet.	N / Insulin dependent T1DM	No patient history<br>No family history	NS	6 months	Y	NS	NS,<br>U(+)<br>NS	NS<br>565<br>NS	NS<br>11.4<br>NS	Y	Y	•IV insulin, hydration and electrolyte management	Y, insulin	NS	Partial	HIV	•Fluoxetine<br>•Trazodone	N
25	Crown et al. (2007) [79]	Polyuria, polydipsia	MT	N	N / New onset T2DM	No patient history<br>No family history	After	18 months	Y	SM (31/OB)	Normal<br>Elevated<br>Normal	NS<br>806.4<br>201.6 (st)	NS<br>14.9<br>NS	Y	Y	•IV insulin, fluids<br>(30/70 28 units, morning; 12 units evening)	Y, insulin, metformin	N	Partial	•Hypertension	•Paroxetine<br>•Clonazepam<br>•Atenolol<br>•Hyrochlorothiazide	N
26	Dahri and Brown (2002) [80]	Thirst, polyuria, polydipsia, nocturia, epigastric pain	PP	Y<br>(NS)	N / New-onset DM	No Patient History<br>Yes family history	N	1 year	Y (both)	NS<br>(40/OW)	NS<br>NS<br>NS	NS<br>496.8<br>NS	NS<br>NS<br>NS	Y	Y	•Insulin	Y, insulin	U	Partial	NS	•Clonazepam<br>•Benztropine	NS
27	Das et al. (2018) [81]	Excessive tiredness, afebrile, normal vitals. Glasgow Coma Scale of 15	MT	Y, amisul.	N / New-onset DM	No patient history<br>No family history	After	10 months	Y	NS<br>(40.9/OB)	NS<br>NS<br>NS	NS<br>443<br>90	NS<br>9.2<br>NS	Y	Y	•Insulin, fluid, saline	Y,<br>metformin	NS	Partial	NS	Valproate	NS
28	de Boer & Gaete (1992) [82]	Stiffness, high fever, difficulty rousing, comatose	PP<br>(Zuclop.)	N/A	N / New onset DM	No patient history	N/A	3 weeks	Y	NS<br>(OW)	NS<br>NS<br>Normal.	155.2 (st)<br>1262 (st)<br>N/A	NS<br>NS<br>N/A	NS	NS	•NS<br>•IV Dantrolene sodium, 100 mg	N/A	N/A	Fatal	NS	NS	Tobacco use

29	Dhamija and Verma (2008) [83]	Polyuria, polydipsia, irritability, changes to behavior and decreased school performance	MT	NS	N / N	No patient history	NS	6 months	Y	I	NS<br>NS<br>Normal	NS<br>535 (st)<br>NS	NS<br>NS<br>NS	NS	Y	•IV insulin, fluids	NS	NS	Full	•Intractable epilepsy<br>•Mental retardation<br>•Mood disturbance	NS	NS
30	Dibben et al. (2005) (Case 1) [84]	Confusion, comatose, eventual paralysis	MT	NS	N / Diabetes insipidus	No patient history<br>No family history	NS	2 years	Y	SM (31/OB)	NS<br>NS<br>Euglycemic	109.8<br>1068.48 (st)<br>126	NS<br>7.2<br>5.5	Y	Y	•IV insulin (15-20 U/H) , ventilatory support	N	Y	Full (diet mgt.)	•Hypothyroidism<br>•Hyperlipidemia<br>•Hypertension	•Lithium,<br>•Thyroxine<br>•Simvastatin,<br>•Aspirin,<br>•Atenolol	N
31	Dibben et al. (2005) (Case2) [84]	Drowsiness, polyuria	MT	NS	N / New onset DM	No patient history	After	8 months	Y (before DKA)	SM, (28/OW)	U(-)<br>NS<br>Normalized	NS<br>1332.6 (st)<br>108	NS<br>13.4<br>6.2	Y	Y	•Insulin, (Mixtard, 30 10U + 14U)	Y, metformin	Y	Full	NS	NS	NS
32	Doodnauth et al. (2021) [85]	Nausea, diffuse abdominal pain, kussmaul breathing and acanthosis nigricans of the neck.	MT	NS	N , Ketone prone type 2 DM	No patient history<br>No family history	N	NS	NS	NS (OB)	NS<br>positive<br>NS	NS<br>1080<br>NS	NS<br>9.7<br>NS	Y	Y	•40U long acting insulin	Insulin	Y	Partial	N	N	N
33	Fulbright and Breedlove (2006) [86]	Lethargy, slurred speech, unsteady gait, weakness	MT	Y, zipras.	N / N	No patient history	NS	7 months	Y	D, (33/OB)	NS<br>NS (U+)<br>NS	100<br>1386<br>99	NS<br>NS<br>3.6	Y	Y	•IV Insulin, fluids ,<br>•Day 3: Subc. insulin	N	Y	Full	•Sleep apnea<br>• Obesity<br>•Hyperlipidemia<br>•Gastroesophageal reflux disease<br>•Hypertension	Divalproex	N
34	Gandhi and Ganesh (2019) [87]	Epigastric pain, nausea, and vomiting, hypertensive, tachycardic, and febrile with significant abdominal pain	PP (Halop.)	N	N / N	No patient history	N	3 months	Y (Quet.)	NS	NS<br>NS<br>NS	NS<br>584.6 (st)<br>NS	5.7<br>NS<br>5.7	NS	NS	•IV hydration and IV insulin	Insulin, temp.	Y	Full	NS	•Divalproex<br>•Benztropine	•Tobacco use<br>•No alcohol/substance use
35	Gatta et al. (1999) [88]	Asthenia, Polyuria, dehydration	PP (Traditional APDs , NS)	N	N / N	No patient history<br>No family history	N	3 months	Y	D (40/OB)	NS<br>NS<br>Normal	NS<br>648<br>NS	NS<br>14.7<br>NS	Y	Y	•IV Insulin, fluids ,(Three insulin injections, 64 U: 3 regular injections and 1 of NPH insulin ,Stopped after 15 days)	N	Y	Partial, (diet mgt.)	N	N	N
36	Goldstein et al. (1999) (Case 1) [89]	Polydipsia, polyuria, dizziness, decreased appetite, diffused abdominal pain, malaise.	MT	Y , quet.	N / NS	No patient history<br>Yes family history	N	6 months	Y	I (36/OB)	Normal<br>U(4+)<br>Normal	NS<br>882<br>NS	NS<br>11.6<br>NS	Y	Y	•IV insulin, fluids ,(NPH insulin 30 U q A.M. and 10 U q P.M., and regular insulin ,10 U q A.M.)	Y	Y	Partial	N	Valproic Acid	N
37	Goldstein et al. (1999) (Case 2) [89]	Flu symptoms, sore throat, cough, malaise, anorexia, nausea, abdominal discomfort ,somnolence, minimally arousable	MT	Y, risp.	N / NS	No patient history<br>No family history	N	17 months	Y	I (27.2/OB)	Normal<br>NS<br>Normal.	NS<br>1160<br>NS	NS<br>NS<br>NS	Y	Y	•IV insulin + hydration ,(NPH, 15 U sc bid, cefotetan (500 mg bid)	N	NS	Full	NS	Cold elixir	N

38	Greenfield et al. (2002) [90]	Gastrointestinal bleeding, pleomorphic vasculitis eruption	MT	Y, risp.	Y, T2DM	Patient history	After	NS	Y	NS	NS<br>NS<br>Controlled	NS<br>993.9 (st)<br>NS	NS<br>13.6<br>NS	Y	Y	•Insulin, hydration	Y, metformin	Y	Partial	Deep venous thrombosis	N	N
39	Hepburn and Brzozowska (2016) [91]	Abdominal pain, vomiting, polyuria, polydipsia	MT	Y, amisul. (briefly); cloz. reinstated to 150 mg/day)	Y, T2DM	Patient & family history	After	13 years	Y, briefly. Reduced dose (150 mg/day)	SM (22.4)	NS<br>NS<br>Controlled	NS<br>469.7 (st)<br>NS	12<br>14.6<br>7.6	Y	Y	•IV iinsulin infusion; insulin injection (glargine (52 units/day and insulin aspart 18 units AM and 22 units lunch and PM,), IV hydration, ,electrolyte repl., deep venous thrombosis prophylaxis	Y, insulin, saxagliptin, metformin	Y	Partial	NS	N	N
40	Hörber et al. (2018) [92]	Abdominal pain, nausea, vomiting, severely altered mental status, restlesness, somnonlence	MT	N	N / T1DM	No patient history<br>No family history	N	Many years	NS	NS (21.7)	NS<br>U(+)<br>NS	NS<br>1889.4 (st)<br>370.7 (st)	NS<br>12.2<br>NS	Y	Y	•IV insulin infusion, IV fluids, sodium bicarbonate	Y, Insulin	NS	Partial	N	Benzodiazepines	N
41	Howes and Rifkin (2004) [93	Thirst, polydipsia, Glasgow Coma Scale: 6	MT	N	N / NS	No patient history<br>Yes family history	N	3 months	N	I (29.3/OB)	Euglycmeic<br>NS<br>Normal.	99<br>1236.6<br>NS	NS<br>NS<br>NS	Y	Y	•Insulin ,(32 units twice daily)	Y	Y	Partial	N	Sodium Valproate	N
42	Hui Fang et al. 2018 [94][3]	Vomitting, abdominal pain, thirst, polyuria, polydipsia, tachycardic, tachypnoeic and drowsy with a Glasgow Coma Scale (GCS) of 10/15.	PP (Quet.)	N	N / Ketone prone DM (temp.)	Family history	Y (temp.)	2 weeks	Y (both)	NS (33.2/OB)	NS<br>NS<br>Normalized	NS<br>826.6 (st)<br>100.8 (st)	NS<br>10.8<br>5.35	Y	Y	•Intravenous insulin, fluids and potassium	Y, insulin, metformin temp.	Y	Partial (diet mgt.)	Depression	•Sertraline,<br>•Pregabalin<br>•Zolpidem	NS
43	Hussain et al. (2024) [95]	Polydipsia, polyuria, generalized weakness, lethargy, abdominal pain	MT	NS	N/NS	NS	NS	NS	NS (temp. before event)	NS	NS<br>NS<br>NS	NS<br>591<br>NS	NS<br>16<br>NS	Y	Y	Insulin drip	NS	NS	NS	NS	NS	NS
44	Itoh et al. (2019) [96]	Thirst, polyuria, drowsiness. ,No acetone breath. ,Mild pain in the left lower quadrant.	MT	Y	N / T2DM	No patient history<br>Yes family history	N	6 months	Y	SM, (22.8/History of OB)	NS<br>(U+)<br>NS	83<br>1709<br>285	6.3<br>13.1<br>5	Y	Y	•IV infusion of insulin/subcutaneous insulin , saline	Y, for 18 days stopped after 18 days of surgery	Y	Partial	•Internal hemorrhoids	•Paroxetine<br>•Mianserin<br>•Tiapride<br>•Suvorexant<br>•Ramelteon	N
45	Iwaku et al. (2017) [97]	Polyuria, polydipsia, oral dryness, general fatigue, loss of appetite, nausea.	PP (Quet.)	Before DKA, quet.	N / New-onset T1DM	No patient history<br>No family history	N	5 months	Y	D (13.6)	Normal<br>U(4+)<br>Improved	NS<br>490<br>NS	NS<br>15.5<br>7	Y	Y	•IV insulin infusion ,• <0.5 U/Kg/day 40 months after discharge	Y, insulin	Y	Partial	NS	NS	NS

46	Jain et al. (2024) [98]	Confusion, polyuria, polydipsia, blood pressure	MT	N	Before, T2DM	Patient history<br>Unknown family history	N	3 months	Y	NS (23)	NS<br>4+<br>NS	NS<br>748<br>201.6 (st)	6.7<br>11.8<br>6.7	Y	Y	•IV fluid resuscitation with 0.9% normal saline<br>10 units of glargine nightly	Y, insulin and metformin temp	Y	Full	Hyperlipidemia	•Duloxetine<br>•Rosuvastatin	NS
47	Jalota et al. (2015) [99]	Abdominal pain, vomiting, polydipsia and polyuria.	MT	Y, amisul.	N / ketone prone T2DM	No patient history<br>Yes family history	After	1 year	Y	I (OB)	NS<br>NS<br>NS	97.2<br>378<br>138.6	NS<br>13.3<br>NS	Y	Y	•IV Insulin, fluid resuscitation<br>•Subc. Insulin	Y, metformin, oral hypoglycemics	Y	Partial	N	N	•Tobacco use<br>•History alcohol abuse
48	Johnson et al. (2002) [100]	Nausea, vomiting, polyuria, polydipsia,	MT	N	N / None	No patient history<br>No family history	N	11 months	N	I (37.8/OB)	U(normal)<br>U(4+)<br>NS	89<br>368<br>164	NS<br>NS<br>6.5	Y	Y	•Insulin, fluids (after discharge: 78 U)	N	Y	Full	NS	NS	NS
49	Juneja et al. (2021) [101]	Nausea, vomiting, epigastric abdominal pain and fevers.	MT	NS	N/ T2DM	No patient history	NS	3 months	Y	NS	NS<br>NS<br>Stabilized	NS<br>952 (st)<br>NS	NS<br>14<br>NS	Y	Y	•IV fluids, insulin drip and potassium repletion	NS	N	Partial	NS	NS	NS
50	Kahn and Bourgeois (2007) [102]	Decreased consciousness, minimal responsiveness, distress. Dried blood in/around the mouth, mild tenderness to palpation in the epigastrium, brown heme-positive stool in the rectum. Glasgow Coma Scale score was 10.	MT	Y, arip	N / N	No patient history<br>Yes family history	After	NS	Y	NS	NS ,<br>U (Large)<br>Normalised	127<br>1,652<br>NS	NS<br>15.2<br>NS	Y	Y	•Insulin, fluids, intubation for airway protection	N, temp. treated with oral hypoglycemic until day 30 of hospital stay	N	Full	NS	•Alprazolam (not prescribed, from a friend)	•Occasional alcohol use<br>•Cocaine abuse
51	Kanagaretnam et al. (2022) [103]	Vomiting, dehydration, drecreased oral intake	MT	N	Before, T2DM (deduced)	Patient history	Before	NS	N	NS	NS<br>NS<br>NS	NS<br>154.6 (st)<br>NS	NS<br>6.2<br>NS	Y	Y	•IV fluids, insulin dextrose infusion	NS	NS	Partial	NS	Metformin	NS
52	Kasmi (2013) [104]	Nausea, vomiting, breathing issues, incontinence ,Glasgow Coma Scale Score: 5	MT	NS	N / NS	No patient history<br>No family history	After	4 weeks	Y	NS (OB)	Normal<br>Elevated<br>NS	NS<br>NS<br>NS	NS<br>NS<br>NS	NS	NS	•Glycemic agents	NS	NS	NS	NS	NS	•Drug overdose<br>•Alcoholism
53	Kibbey et al. (2010) [105]	Lethargy, dry cough, polyuria, polydipsia, disorientation, volume depletion, afebrile, tachypneic, weight loss.	MT	N/A	N / NS	No family history	N/A	12 months	N (half dose)	I (40/OB)	NS<br>NS<br>NS	NS<br>874.9 (st)<br>145.8	NS<br>15.9<br>NS	Y	Y	•IV insulin infusion, fluids<br>•At discharge: 330 U/day)	Y, insulin	NS	Partial	N	N	N

54	Kinoshita et al. (2014) [106]	Nausea, vomiting, thirst	MT	NS	N / NS	No patient history<br>No family history	N	3 months	Y	NS	Normal,<br>NS(U+)<br>Controlled	NS<br>925.2 (st)<br>112 (st)	5.3<br>9.6<br>6	Y	Y	•Insulin, hydration ,(37-44 units,insulin injections, 4 X / day,Day 27: Insulin Glulisine and Insulin Glargine)	Y, insulin until day 128	Y	Full	N	Duloxetine	History of tobacco use
55	Kostakoğlu et al. (1996) [107]	Weakness, sweating, anorexia, blurred vision, diplopia, state of stupor	MT	Y, fluph.	N / NS	No Patient History<br>Yes Family history	N	4 weeks	Y	NS (OB)	NS<br>U (4+)<br>NS	121<br>447<br>93	NS<br>NS<br>NS	Y	Y	•IV insulin and hydration,•Day 4: regular insulin injections - 40 units of isophane, 20 units of regular insulin (morning); 20 units of isphone, 10 U of regular insulin (evening).	N	Y	Partial (diet mgt)	NS	NS	NS
56	Koval et al. (1994) [108]	Comatose	MT	Y, halop.	N / N	No patient history<br>Yes family history	After	6 weeks	Y	NS	Normal<br>U(+)<br>NS	NS,<br>1124<br>120	NS<br>NS<br>NS	Y	Y	•IV insulin and hydration, ventilation support ,(At discharge: 13 U morning)	N	Y	Full	N	•Lithium carbonate<br>•Benztropine,	N
57	Kyriazis et al. (2006) [109]	Intense epigastric pain, food vomiting, tachypnea	MT	Y, risp.	N / NS	No patient history<br>Yes Family history	N	4 months	Y	NS (37/OB)	Normal<br>NS<br>Low	NS<br>478<br>300	NS<br>NS<br>NS	Y	Y	•IV insulin, fluids, NPH insulin inejction subc: 30-20 U for 5 days)	N	Y	Partial (diet mgt.)	N	N	N
58	Lafayette et al. (2003) [110]	Lethargy, menstrual spotting, cramping, breast tenderness, nausea and vomiting. ,Hyperventilation, shortness of breath, chest discomfort	PP (Risp.)	N	N / NS	No Patient history<br>Yes Family history	N	10 weeks	Y, (cloz)	D (33/OB)	Normal<br>NS<br>NS	123<br>447<br>93	NS<br>12.2<br>NS	Y	Y	•IV insulin,(Subc. 38 units of isophane insulin b.i.d. + 20 units of regular insulin b.i.d)	N	Y	Partial (diet mgt.)	N	•Lorazepam,<br>•Risperidone,<br>•Benztropine,<br>•Multivitamin /calcium supplements<br>•medroxyprogesterone	Substance dependence
59	Laghate and Gupta (2004) [111]	Vomiting, abdominal pain and heavy breathing	PP	N	N / NS	Family history	N	NS	Y, both	NS	NS<br>U(+)<br>Norm.	NS<br>580<br>NS	NS<br>NS<br>NS	Y	Y	•IV insulin, fluids, ceftazidime, metronidazole, potassium chloride, and calcium gluconate	N	Y	Full	NS	Sodium Valproate	NS
60	Lim et al. (2025) [112][3]	Nausea, vomiting	MT	Y, zipra.	NS / NS	NS	NS	2 years	Y	I	NS<br>U (+)<br>NS	NS<br>1926.4 (st)<br>NS	NS<br>13<br>NS	Y	Y	•Fluids, subc. insulin glargine (Lantus) 20 units nocte and insulin aspart (NovoRapid) 5 units three times daily with meals.	NS	NS	NS	NS	NS	NS
61	Lindenmayer and Patel (1999) [113]	Drowsiness, lethargy, unresponsiveness to verbal commands.	PP (Fluph.)	N	N / NS	No patient history<br>No family history	N	6	Y	D (OB)	Normal<br>NS<br>Norml.	NS<br>1344 (st)<br>NS	NS<br>NS<br>NS	NS	Y	•IV insulin, NPH, 40-70 units for 15 days<br>5% dextrose fluid	N	NS	Full	•Hypertension<br>•Myocardial infarction	Divalproex sodium	Substance dependence
62	Lu and Yan (2009) [114]	Fever	MT	N/A	N / NA	No patient history	N	2 months	Y	NS	Norm.<br>NS<br>N/A	NS<br>1297<br>NS	NS<br>13.7<br>N/A	Y	Y	•IV Insulin, fluids	N/A	N/A	Fatal	NS	NS	NS

63	Macfarlane and Fisher (2006) [115]	Dyspnea, drowsiness, confusion, polyuria, polydipsia,agitation, tachypnoea, tachycardia, cold and clammy peripheries	PP (Quet.)	N	N / NS	No patient history<br>No family history	N	2 years	Y	D (29/OB)	NS<br>NS<br>NS	NS,<br>1422<br>NS	NS<br>NS<br>5.4	Y	Y	•IV insulin, fluids,(biphasic insulin regimen	Y, insulin	Y	Partial	NS	Folic Acid	•History of drug abuse,<br>•History of alcohol abuse
64	Madsen (2014) [116]	Thirst, vomiting, abdominal pain, delirum, pallor, diaphoresis, cool extremities, fever	MT	N/A	N / N/A	No patient history<br>No family history	N	1 year	N/A	NS (34/OB)	Normal<br>U(4/4)<br>N/A	NS<br>564.5 (st)<br>N/A	NS<br>NS<br>NS	Y	N/A	•Saline, insulin infusion	N/A	N/A	Fatal	N	None	N
65	Mahmoud et al. (2023) [117]	Nausea, vomiting, severe back pain, fevers, hypertension, high blood pressure, tachycardia, tachypnea	MT	NS	N / NS	No patient history	NS	NS	NS	NS	NS<br>NS<br>NS	NS<br>735<br>NS	NS<br>NS<br>NS	NS	Y	•IV fluids, insulin infusion	NS	NS	NS	NS	NS	NS
66	Makhzoumi et al. (2008) [118]	Urinary incontinence, somnolence, drowsiness, difficulty arousing, lethargy, difficulty moving upper extremities and walking, refusal if nutrition by the mouth, tachycardia	PP (Fluph.)	Y, halop (as needed); fluph. (reinstated)	N / NS	No patient history<br>No family history	After	16 days	Y	D (41.1/OB)	NS<br>NS<br>NS	122<br>813<br>215	NS<br>14.9<br>NS	Y	Y	•IV insulin, fluids.<br>Subc. long acting	Y, insulin	Y	Partial	•Hyperlipidemia	•Valproic acid<br>•Benztropine<br>•Atorvastatin	N
67	Maksimoviæ and Pavliæ-Renar (2006) [119] (Case 2)	Malaise, abdominal pain, nausea, vomiting, polydipsia, polyuria	PP, (Risp./Prom.)	N	N / NS	No patient history<br>No family history	N	NS	N	NS (37.81/OB)	NS<br>U(+)<br>Norm.	NS<br>610.9 (st)<br>NS	NS<br>12.9<br>NS	Y	Y	•IV insulin, fluids	Y	N	Full	N	N	Tobacco use
68	Marlowe et al. (2007) [120]	Decreased consciousness, Somnolence,Arousable	MT	NS	N / N	No patient history	After	6 months	Y	SM (27.6/OB)	Normal,<br>(U+)<br>Normalized	99<br>1492<br>90	NS<br>NS<br>7	Y	Y	•IV insulin, fluids	N	Y	Full	NS	Valproic acid	NS
69	Maule et al. (1999) [121]	Vomiting, obtusion dehydration, comatose	MT	N	N / N/A	No patient history<br>Yes family history	N	30 days	N, (Reduced dose)	NS	NS<br>U(4+)<br>NS	NS<br>1000<br>108	NS<br>NS<br>NS	Y	Y	•Insulin and fluids,(Subc: 32 U)	Y, insulin but withdrawn due to hypoglycemia	NS	Partial (diet mgt)	N	Valproic acid	N
70	McCalmon and Weide (2021) [122]	Altered mental staus	MT	N	N / T1DM	NS	N	1 month	N	NS	Normal<br>NS<br>NS	100.8 (st)<br>547.7 (st)<br>NS	NS<br>12.3<br>NS	Y	Y	•IV fluid, potassium replacement, insulin drip	Insulin	Y	Partial	NS	NS	NS
71	Miller et al. (2008) [123]	Vomiting, lethargy, fatigue, thirst, increased urination	PP (Zipra.)	N	N / New onset Type 2 DM	No patient history<br>No family history	NS	3 months	N	D (37/OB)	NS<br>NS<br>NS	92<br>722<br>NS	NS<br>NS<br>7.5	Y	Y	•Insulin (NPH insulin: 35 U, twice a day)	Y, insulin	NS	Partial	•Hypertension<br>•Gastroesophageal,reflux disease	•Aspirin<br>•Lactulose,<br>•Lamotrigine<br>•Loratadine	Tobacco use

																				•Diverticulosis<br>•Chronic cough	•Nifedipine<br>•Omeprazole	
72	Mithat et al. (2005) [124]	NS	MT	NS	N / N	No patient history<br>No family history	N	6 months	Y	I (32.7/OB)	Normal<br>NS<br>Normal	NS<br>647<br>102	NS<br>NS<br>NS	Y	Y	•IV insulin, fluids, potassium ,(1.15 u/kg as IV bolus + 0.1 units.kg-1.h-1 IV insulin infusion)	N	Y	Full	N	•Valproate<br>•Lithium	N
73	Mohan (1999) [125]	Sore throat, lethargy, slurred speech, thirst	MT	Y, risp. (discontinued); typical APDs	N / Y	No patient history<br>No family history	After	3 months	Y	NS	NS<br>NS<br>Controlled	NS<br>383.4 (st)<br>NS	NS<br>NS<br>NS	NS	NS	NS	Y, glibenclamide	Y	Partial	NS	NS	NS
74	Muench and Carey (2001) [126]	Polyuria, polydipsia, nausea, vomiting, dizziness	MT	NS	N / New onset T2DM	Yes patient history<br>Unknown family history	Before	12 months	N	I (31/OB)	NS<br>High<br>NS	97<br>765<br>224 (st)	NS<br>13.4<br>NS	Y	Y	•IV insulin, fluids,•Subc. insulin (80 U-morning; 35 U-evening),•half dose after 2 months	Y, insulin	Y	Partial	History of Hepatitis A	•Venlafaxine<br>•Valproic acid<br>•Atorvastatin<br>•Propranolol	•Tobacco use<br>•History of substance abuse (meth.)
75	Murakami et al. (2025) [127]	Impaired consciousness, abdominal distension, urinary retention, difficulty walking, repeatedly fell, numbness in both lower limbs, Achilles tendon reflex and sense of vibration were reduced in both lower limb, hypertension	PP, (Levom.)	NS	Before, DM	Patient history	Y	NS	Y, (Olanz.)	NS	NS<br>4+<br>Stabilized	NS<br>919<br>150	NS<br>17.6<br>NS	Y	Y	•Fluids Insulin infusion (8 units per day)	Y, metformin (1000 mg/day)	Y	Partial	Neuropathy	Nitrazepam	NS
76	Nagamine (2021) [128]	Drowsiness	MT	NS	N / N	No Patient history<br>No family history	N	8 weeks	Y	SM (23)	NS<br>NS<br>NS	NS<br>545.4<br>102.6	5.3<br>7.6<br>NS	Y	Y	Insulin	N	Y	Full	NS	NS	NS
77	Nahas et al. (2010) [129]	Polyuria, polydipsia, blurred vision, nausea, emesis	MT	Y, arip.	N / NS	No patient history<br>Yes family history	N	3 years	Y	NS	NS<br>NS<br>NS	NS<br>715<br>NS	NS<br>10.3<br>NS	Y	Y	•IV insulin, fluids, saline	Y, insulin	NS	Partial	NS	NS	NS
78	Nakanishi et al. (2025) [130]	Depression	MT	NS	N / Ketone-prone T2DM	No family history	After	9 years	Y	D (32.8/OB)	NS<br>U+<br>Controlled	NS<br>284<br>95	NS<br>12.8<br>6.7	Y	Y	•Subcutaneous insulin, 57 units/day	Y, metformin and linagliptin	NS	Partial	Depression	NS	NS
79	Ng and Broussard (2024) [131]	Polyuria, polydipsia, abdominal discomfort, nausea, fatigue	MT	NS	N / NS	No patient history<br>No family history	NS	2 weeks	NS	NS	NS<br>NS<br>NS	NS<br>718<br>NS	NS<br>14<br>NS	NS	Y	•Fluid resuscitation, insulin drip	NS	NS	NS	Hypertension	NS	NS

80	Niazy et al. (2007) [132]	Stupor, consfusion, dehydration, polyuria and polydipsia, drowsiness, arousable	MT	Y, risp.	N / NS	No patient history<br>No family history	N	18 months	Y	I (37.7/OB)	NS<br>U(3+)<br>Normal.	NS<br>794.4 (st)<br>NS	NS<br>NS<br>NS	Y	Y	•Insulin, 2 doses of insulin Mixtard, IV fluids, potassium supplements	N	Y	Full	•Hyperlipidemia<br>•Hypertriglyceridemia	NS	NS
81	Nicolai et al. (2001) [133]	Unconsciousness, roving eye movement	MT	Y, halop.	NS / NS	NS	N	5 years	Y	NS	NS<br>NS<br>Normal.	NS,<br>1717.2<br>NS	NS<br>NS<br>NS	Y	Y	•Insulin, fluid and potassium supplementation,(NS)	N	NS	Full	N	Valproate	N
82	Ogunnaya et al. (2024) [134]	Febrile (100.8F), hypertensive, tachypneic, and tachycardic, mild distress.	MT	NS	Before, DM	Patient history	NS	NS	NS	NS	NS<br>NS<br>NS	NS<br>576.8 (st)<br>NS	NS<br>NS<br>NS	Y	Y	•Insulin, fluids	NS	NS	Partial	•Seizures,<br>•Stroke<br>•Systemic hypertension	N	NS
83	Patel et al. (2011) [135] (Case 1)	NS	MT	NS	N / NS	NS	After	3	Y	NS (OB)	NS<br>NS<br>Normal.	NS<br>1249.9 (st)<br>NS	6.5<br>NS<br>NS	NS	Y	•IV insulin,,(60 U/day)	Y, insulin, metformin	NS	Partial	NS	NS	NS
84	Pathmanathan & Somasekharan (2013) [136]	Nausea, vomiting, polyuria, polydipsia	MT	N/A	Before T2DM	Patient history	Before	NS	N, (Reduced dose)	NS	Controlled<br>NS (U+)<br>High	NS<br>452<br>NS	7.4<br>14.2<br>NS	Y	Y	•IV insulin, fluids ,(48 U)	Y, insulin	NS	Partial	Hypertension	Metformin	NS
85	Peterson and Byrd (1996) [137]	Stupor	MT	NS	N / T2DM	Family history	N	5 weeks	Y	SM	Normal<br>U(+)<br>NS	NS<br>762<br>NS	NS<br>NS<br>NS	Y	Y	•IV insulin and rehydration	Y	Y	Partial	Hypertension	•Lithium carbonate<br>•Bethanechol<br>•Verapamil	•History of substance use<br>•History of alcohol use
86	Pierides (1997) [138]	Lethargy, thirst, chest pain, dyspnoea	MT	NS	N / NS	NS	N	10 days	Y	NS	Normal<br>NS<br>Improved	NS<br>423<br>NS	NS<br>NS<br>NS	NS	Y	•Subc. insulin	NS	NS	NS	NS	N	NS
87	Pillai et al. (2006) [139]	Thirst, exhaustion, frequent urination, drowsiness, unresponsiveness, unrecordable blood pressure, emancipation, unconsciousness	PP (Risp.)	N/A	N / N/A	Family history	N	3 months	N/A	D	Normal<br>U(+)<br>NS	NS<br>465.9 (st)<br>224 (st)	NS<br>8.9<br>N/A	Y	Y	•IV insulin, fluids, ventilation, antibiotics, PCV.	N/A	N/A	Fatal	N	N	N
88	Popli et al. (1997) [140]	Hypotension, obtusion, dehydration	MT	N/A	N / NS	Family history	After	8 weeks	Y (Briefly)	I (OW)	NS<br>NS<br>NS	NS<br>930<br>143	NS<br>NS<br>NS	Y	Y	•Insulin and IV fluids	Y, glyburide	Y	Partial	NS	Ephedrine	•History of substance abuse
89	Ragucci and Wells (2001) [141]	Abdominal pain, dizziness, polyuria, dyspnea	MT	Y, risp.	N / NS	No patient history<br>Yes family history	After	14 months	Y	SM (39/OB)	NS<br>U(+)<br>Normal.	83<br>1071.8 (st)<br>NS	NS<br>11.7<br>5.8	Y	Y	•IV insulin, fluids 33/17 units (morning) and 16/9 units (evening)	Y. metformin. insulin slowly discontinued	Y	Partial	•Hypertension	•Valproic acid<br>•Carbamazepine<br>•Hydrochlorothiazide/triamterene<br>•Conjugated estrogens	NS
90	Rahat et al. (2005) [142]	Polyuria, polydipsia, unconsciousness, agitation, dehydration, hyperpyrexia, stiffness	MT	N	NS / NS	NS	N	1 year	N	NS	NS<br>NS<br>NS	NS<br>616 (st)<br>NS	NS<br>NS<br>NS	Y	N	•Life support management, physostigmine and centrally acting cholinergic	N/A	NS	Full	Depression	N	NS

91	Rashid et al. (2009) [143]	Abdominal pain, polyuria, polydipsia, vomiting.	PP (Zipras.)	Y, halop.	N / NS	No patient history<br>No family history	NS	7 months	Y (both)	SM (OB)	NS<br>NS<br>NS	NS<br>1210.7 (st)<br>NS	NS<br>NS<br>NS	Y	NS	NS	NS	Y	NS	PCOS	NS	N
92	Ratnakaran (2015) [144]	Drowsiness, slurred speech, disorientation, decreased response to questions, increased psychomotor activity. Afebrile. No signs of dehydration, no odor in breath.	MT	N	N / NS	Family history	After	1 day	Y	NS (23)	NS<br>Elevated U(+)<br>NS	NS<br>363<br>173.6 (st)	NS<br>NS<br>NS	Y	Y	•IV insulin and saline (subcutaneous: 6 U (morning/pm); 4 U (evening))	Y, glibenclamide, metformin	NS	Partial	N	•Escitalopram<br>•Clonazepam	N
93	Reddymasu et al. (2006) [145]	Fatigue, dyspepsia, epigastric pain	MT	Y, halop.	N / New onset DM	No patient history<br>No family history	N/A	18 months	Y	I	NS<br>NS<br>NS	NS<br>1981.3 (st)<br>NS	NS<br>NS<br>NS	NS	Y	•IV insulin, fluids	Y, Insulin	Y	Partial	NS	NS	N
94	Sa et al. (2013) [146]	Hyperthermia, nausea, vomiting, mental deterioration	MT	NS	N / NS	No patient history<br>No family history	After	32 months	Y	I (31.7/OB)	Normal<br>NS<br>NS	NS<br>1216<br>200	NS<br>13.8<br>6.2	Y	Y	•IV insulin, fluids •≥ 4 L/day	N	Y	Partial (lifestyle changes)	N	•Valproic acid<br>•Clonazepam<br>•Lorazepam	N
95	Sato et al. (2008) [147]	Loss of consciousness	MT	U	N / New onset T1DM	Family history	N	4 months	NS	SM (21.9)	NS<br>NS<br>Improved	138<br>926<br>NS	NS<br>12.2<br>NS	Y	Y	•Insulin, IV fluid	Y	Y	Partial	Acute Polyendocrine Syndrome	NS	NS
96	Seaburg et al. (2001) [148]	Agitation, incontinence, polydipsia, polyphagia, nausea, vomiting	MT	N	N / New onset	No patient history	After	29 months	N	D (27/OB)	NS<br>NS<br>NS	NS<br>1388.8 (st)<br>122	NS<br>NS<br>7.5	Y	Y	•Insulin and rehydration (Subcutaneous 70/30 insulin 40 U (morning) and 20 U (evening)	Y, Pioglitazone, metformin	Y	Partial	•Seizure disorder<br>•Developmental disability	Valproic Acid	N
97	Selva & Scott (2001) [149]	Lethargy, vomiting, inability to walk	PP (Risp.)	N	N / T2DM	Family history	N	6 months	Y	I (OW)	NS<br>Significant (U+)<br>Increasing	NS<br>669<br>NS	NS<br>17.7<br>10.5	Y	Y	•IV insulin (0.05 U/kg/ hour)	NS	Y	Partial	DiGeore syndrome	Venlafaxine	NS
98	Shin et al. 2025 [150]	Weakness, shortness of breath, thirst, frequent urination, dysphagia	PP (Quet./Amisul.)	N	NS / NS	No patient history<br>No family history	After	2 months	Y (Cloz.)	D (30.1/OB)	NS<br>NS<br>Stabilized	NS<br>500<br>NS	NS<br>11.5<br>NS	Y	Y	•Aggressive hydration (>300 mL/h) basal insulin	Y, basal insulin and oral diabetes medication	Y	Partial	N	•Ropinirole<br>•Benztropine	N
99	Singh et al. (2013) [151]	Nausea, vomiting and abdominal pain	PP (Halop.)	Y	N / N	No patient history<br>No family history	N	13 days	Y	NS	NS<br>NS(U+)<br>NS	89<br>625<br>280 (st)	NS<br>NS<br>NS	Y	Y	•IV insulin and fluid (NPH evening; and three premeal regular insulin,	N	Y	Full	NS	NS	NS
100	Sirois (2008) [152]	Glasgow Scale 5/15	PP (Risp.)	NS	N / NS	No patient history<br>No family history	NS	37 days	NS	NS (37.2/OB)	Normal<br>NS<br>Normalized	104.4<br>1,472.4<br>NS	NS<br>NS<br>NS	Y	Y	Insulin	Y, insulin	NS	Partial	NS	Venlafaxine	NS
101	Skwiersky et al. (2020) [153]	Generalized weakness, unsteady gait, dry mouth, polydipsia, polyuria	MT	NS	N / New onset DM	No patient history	N	10 months	Y	D	NS<br>NS<br>NS	NS<br>519.7 (st)<br>NS	5.3<br>20.1<br>NS	Y	Y	•Fenofibrate Insulin galargine as per DKA guidelines	Insulin drip	NS	NS	N	N	NS
102	Smith et al. (1999) [154]	Excessive sedation, nocturnal incontinence, postural hypertension,	MT	NS	N / NS	No patient history<br>No family history	N	16 days	NS	NS	NS<br>NS<br>Resolved	NS<br>1108.8 (st)<br>NS	NS<br>NS<br>NS	Y	Y	•Insulin (Insulin mixtard, 30 units twice daily)	Y, insulin	Y	Partial	NS	NS	NS

		confusion, smell of ketones.																				
103	Straker et al. (2002) [155]	Gastrointestinal distress, deterioration of mental status, hypotension	PP (Halop.)	Y, zipras.	N / NS	No patient history<br>No family history	After	1 month	Y (Olanz.)	I	NS<br>NS<br>NS	155<br>560 (st)<br>168 (st)	NS<br>5.9<br>NS	NS	Y	•IV Insulin drip (NPH insulin)	N	NS	Partial (diet mgt.)	•Hepatitis C<br>•Seizure disorder	Phenytoin	•Substance abuse<br>•Alcohol dependence
104	Strassnig et al. (2013) (Case 1) [156]	NS	MT	Y, typical APD	N / Transient T2DM	NS	After	6 months	Y	I	NS<br>NS<br>NS	NS<br>660.6<br>73.8	NS<br>NS<br>NS	NS	Y	•Insulin injections, oral hypoglycemics	N	Y	Full	NS	NS	NS
105	Takahashi et al. (2005) [157]	Somnolent, difficulty with speech	PP (Tiapride)	NS	N / N	No patient history<br>No family history	After	11 days	NS	NS (21.3)	NS<br>NS<br>Controlled	132<br>973<br>NS	NS<br>NS<br>NS	Y	Y	•IV insulin and fluids (Subc. insulin injection, 34-20 i.u./d for two weeks)	Y, glibenclamide, (temp).	Y	Full	NS	N	History of alcohol abuse
106	Taslipinar et al. (2008)[±] [158]	Confusion, lethargy	MT	Y, zipras.	N / N	No patient history<br>No family history	N	5 months	Y	NS (31/OB)	NS<br>NS<br>Normal.	NS<br>338<br>NS	NS<br>11.2<br>NS	Y	Y	•IV insulin and hydration (Intermittent subcutaneous insulin)	N	Y	Full	NS	NS	NS
107	Tavakoli & Arguisola (2003) [159]	NS	MT	NS	N / T1DM	Family history	NS	18 months	Y	I (OW)	NS<br>NS<br>NS	105.3 (st)<br>NS<br>NS	NS<br>NS<br>NS	NS	NS	NS	Y, insulin	Y	Partial	NS	•Valproic acid<br>•Venlafaxine	•History of Substance Abuse<br>•History alcohol abuse
108	Thanikonda et al. (2020) [160]	Polyuria, polydipsia, blurriness of vision, and dry mouth	MT	N	N / N	No patient history<br>Yes family History	N	13 years	N	D	NS<br>NS<br>NS	NS<br>1247.7 (st)<br>NS	NS<br>NS<br>NS	NS	Y	•Hydration, Insulin drip	NS	NS	Partial	NS	•Trileptal,<br>•Cogentin	NS
109	Torrey and Swalwell (2003) [161]	NS	PP (Risp.)	N/A	N / N/A	No patient history	N	1 month	N/A	NS	Normal<br>N/A<br>N/A	NS<br>N/A<br>N/A	NS<br>NS<br>N/A	Y	N/A	N/A	N/A	N/A	Fatal	N	•Lithium<br>•Fluoxetine<br>•Bupropion	•History of alcohol abuse<br>•History of substance abuse
110	Tsuchiyama et al. (2004) [162]	Thirst, malaise, mild confusion	MT	N	N / N	Family history	N	1 month	Y	D (28.7/OB)	NS<br>NS<br>Improved	NS<br>1209.6 (st)<br>140	NS<br>13.7<br>NS	Y	Y	•Insulin (100 U/day)	N	Y	Full	NS	NS	NS
111	Tugwell et al. (2020) [163]	Weakness, drowsiness, volume depletion	MT	N/A	Y, gest. DM / ketone prone T2DM	Yes patient history<br>Yes family history	N	4 months	Y	I (35/OB)	NS<br>NS<br>Normal	127.8<br>896.4<br>NS	NS<br>15.8<br>5.8	Y	Y	•Insulin	Y, metformin,empagliflozin	Y	Partial	NS	•Clonazepam<br>•Valproic acid	N
112	Varma et al. (2007) [164]	General malaise, drowsiness, difficulty rousing, tachycardic, hypotensive, dehydration. Glasgow score of 3	MT	Y, risp.	N / N/A	No patient history	N	6 weeks	Y	NS (28.4/OB)	NS (off scale)<br>U(+)<br>NS	NS<br>1844.6 (st)<br>189.2	NS<br>13.2<br>NS	Y	Y	•IV insulin, fluid, electrolyte resuscitation<br>•Broad spectrum antibiotics	Y	NS	Partial	N	NS	N
113	Vincent et al. (2017) [165]	Vomiting, nausea, fatigue, weakness, abdominal pain, dehydration	MT	Y, cloz.	N / NS	No patient history	N	1 year	Y	NS	NS<br>NS<br>Reduced	NS<br>457<br>195	NS<br>15.3<br>NS	Y	Y	•Insulin, fluids	NS	NS	Unsure	NS	NS	NS

114	Vuk et al. (2017) [43]	High blood pressure, tachycardia, disorientation, vomiting, somnolence, hypotension, tachycardia, tachypnea acetone smell	PP (Halop.)	Y, amilsul.	N / NS	No patient history<br>No family history	After (temp.)	3 weeks	Y (Cloz.) (Halop. briefly/reduced dose)	I	NS<br>NS<br>Normal	115.2<br>1024.1<br>NS	NS<br>8.5<br>NS	Y	Y	•Standard therapy (Long acting insulin, 10 IU)	Y	Y	Partial (diet mgt)	NS	Bisoprolol	NS
115	Waldman and Yaren (2002) [166]	Paleness, dyspnea	MT	Y, quet., then cloz.	N / NS	No patient history	After	3 months	Y	I	NS<br>NS<br>Fluct.	117<br>756 (st)<br>NS	NS<br>NS<br>NS	Y	Y	•Insulin	Y, glyburide	Y	Partial	NS	N	NS
116	Watkins et al. (2011) [167]	Altered mental status, nausea, vomiting, abdominal pain, slurred speech	MT	NS	N / New onset DM	No patient history	N	6 months	Y	I	NS<br>NS<br>NS	132<br>799.7 (st)<br>171.4 (st)	NS<br>13.5<br>NS	Y	Y	•IV insulin, saline (5 units/ twice per day)	Y, insulin	NS	Partial	Hyperlipidemia	Sertraline	N
117	Whicher et al. (2019) 45]	Vomiting, abdominal Pain, dehydration	PP (Arip.)	N/A	NS / T2DM	NS	Y	5 years	NS	NS	NS<br>NS<br>NS	NS<br>483.8 (st)<br>NS	NS<br>NS<br>NS	Y	Y	•Insulin	Y, metformin	Y	Partial	NS	•NS	N
118	Wilson et al. (2002) (Case 1) [168]	Thirst, urination, confusion	MT	N	N / NS	No patient history<br>No family history	N	40 weeks	N	I (26.5/OB)	NS<br>NS<br>Controlled	NS<br>~~483.8~~<br>404.3 (st)<br>NS	NS<br>NS<br>NS	NS	Y	•Low-dose insulin (5 U/day)	NS	Y	Unsure	NS	•NS	NS
119	Wilson et al. (2002) (Case 2) [168]	Thirst, urination, confusion	MT	NS	N / NS	No patient history<br>No family history	N	8 weeks	NS	I (34.7/OB)	NS<br>NS<br>Controlled	NS<br>647.4 (st)<br>NS	NS<br>NS<br>NS	NS	Y	•IV fluid and insulin	N	Y	Partial (diet mgt.)	•Mental retardation	•Lithium<br>•Valproic Acid	•History of alcohol abuse
120	Wilson et al. (2002) (Case 3) [168]	Unresponsiveness	MT	NS	N / NS	Unknown patient history<br>Yes family history	N	8 weeks	NS	I (30.2/OB)	NS<br>NS<br>NS	120<br>NS<br>NS	NS<br>NS<br>NS	NS	NS	NS	NS	Lost to follow up	Unsure	•Chronic obstructive pulmonary disease<br>•Prostate cancer	•Propranolol	NS
121	Wilson et al. (2002) (Case 4) [168]	Thirst, obuntusion	PP (Cloz.)	N, cloz. continued	N / NS	No patient history<br>No family history	N	7 weeks	Y (Risp.)	I (40.1/OB)	NS<br>NS<br>NS	NS<br>489.4 (st)<br>NS	NS<br>NS<br>NS	NS	Y	•Insulin therapy (Insulin 70/30 units/ day)	N	Y	Partial (diet mgt)	NS	•Mesoridazine<br>•Clonazepam<br>•Lithium<br>•Venlafaxine	•History of substance abuse
122	Wilson et al. (2002) (Case 5) [168]	Disorientation	PP (Olanz.)	N	N / NS	No patient history<br>No family history	N	4 weeks	N	I (36.8/OB)	NS<br>NS<br>NS	NS<br>355 (st)<br>NS	NS<br>NS<br>NS	NS	Y	•Insulin	Y, insulin	Y	Partial	NS	N	NS
123	Wong et al. (2007) [169]	General and chest discomfort, fatigue, vomiting, nausea, dehydration, breath smelled like ketones.	MT	Y, halop.	N / NS	No patient history<br>No family history	N	39 months	Y	I (28.5/OB)	Normal<br>U(+)<br>NS	NS<br>721.8<br>75.6	NS<br>11.9<br>5.7	Y	Y	•IV insulin, fluids, and electrolytes (29 U morning; 16 units evening)	Y	Y	Partial (diet mgt.)	NS	•Sodium valproate	NS

Abbreviations: BMI, body mass index; HbA1c, hemogolgin A1c; Pt, Patient; MT, monotherapy; OB, obesity; OW, overweight ; PCOS, polycystic ovary syndrome; PP, polypharmacy; +, Positive; subc, subcutaneous; temp, temporarily.
Amisul, Amisulpiride; Arip, Aripiprazole; Cloz, Clozapine; Flup, flupentixol; Fluph, Fluphenazine; Halop, Haloperidol; Olanz, Olanzapine; Levom, levomepromazine; Prom, Promazine; Quet, Quetiapine; Risp, Risperidone; Zipra, Ziprasidone; Zuclo, Zuclopenthixol.
D, decrease; I, increase; N, no; NS, not specified; Y, yes.
[1] One DKA case was identified through supplemental searching outside the predefined search strategy.
[2] The case report stated few months from the administration of the APD to time to complication; therefore, a mid-point of 6 months was assumed.
[3] Two cases were diagnoses as demonstrating both DKA and HHS and are included [94,112]
[4] Added after cross reference with Vuk et al. (2017) [158].
[5] Reinstatment of the APD is not considered a discontinuation for the purpose of this analysis.
[6] Standardized blood glucose values are shown (st).
[7] Complications occurring at or around the same time as DKA are not discussed (e.g., Neuroleptic Malignant Syndrome, pancreatitis).

Table S3. Summary of the characteristics of patients diagnosed with hyperglycemic hyperosmolar state associated with antipsychotic drug se (n = 30)[1-5].

Pt No	Citation	Clinical Signs and symptoms	Type of APD Therapy	APD Switch HHS	DM Diagnosis before/after complication	History of DM	Antidiabetic before / after HHS event[1]	Time to Complication (estimates when not stated)	APD Discontinued[3]	Overall Weight Changes (BMI, kg/m[2]/ Qualitative)	Glucose (Qualitative): •Before complication •At presentation •After resolution	Plasma glucose (Quantitative, mg/dl)[4] •Before complication •At presentation •After resolution	HbA1c (%) •Before complication •At presentation •After resolution [1]	Ketone +	Osmolality /Osmolarity (Units as Reported)	Treatment of HHS with Insulin	Treatment for HHS (Last recorded insulin dosage if specified)	Treatment with insulin or antidiabetic required after resolution	Was patient followed up?	Recovery after resolution	Co-morbidities[5]	Concomitant medication (other than APDs)[2]	Other known issues
1	Ahuja et al. (2010) [170]	•Polyuria, polydipsia, malaise, drowsiness, •Vomiting, unresponsive	MT	Y, risp.	N/ T2DM	No patient history No family history	N	5 weeks	Y	SM	NS U(+) Normal	99 1,647 NS	NS 13.2 NS	Y	373 mOsm/kg	Y	•Insulin, ventilator support and fluids	Y, Insulin	Y	Partial	NS	N	N
2	Balzan and Cacciottolo (1992) [171]	•Dehydration, muscle rigidity, Parkinsonian tremor, unresponsiveness , Comatose	PP (Triflu.)	NS	N / Y	No patient history	N	5 days	NS	NS	NS, U (heavy) Normal	NS 907.2 (St) NS	NS NS NS	N	368 mOsm/kg	Y	•IV insulin, fluids (40 U / Day)	Y, Insulin	N	Partial	N	•Tricyclic antidepressants	N
3	Campanella et al. (2009) [172]	•Cough, lethargy, confusion, waning consciousness. •Mild tongue fasciculations, fine resting tremors. •Gross purposeful movements with all 4 extremities, slurred speech, etc.	MT	Y, other APDs (NS)	N / N	No patient history	N	NS	Y	NS	NS Present U+) Normal	NS 2845 NS	NS NS NS	NS	NS	Y	•IV insulin, saline, antibiotics, oxygen, ventilation support, etc. (Subc: 10 U)	N	Y	Full	•Chronic alcoholism with multiple episodes of withdrawal	•Cogentin •Depakoate ER •Diphenhydramine hydrochloride	Alcoholism
4	Cerimele (2008) [173]	•General Malaise, vomiting, polyuria, polydipsia	MT	N	N / T2DM	No patient history	N	36 months	Y	I (OB)	NS NS NS	NS 1179 NS	NS 14.6 NS	NS	378 mOsm/kg	Y	•Insulin, fluids, electrolyte management	Y	NS	Partial	•Hypertension, •Hypercholesterolemia, •Seizure disorder	•Carbamazepine, •Hydrochlorothiazide •Ranitidine •Atenolol •Paroxetine	N
5	Chen et al. (2003) [174]	•Malaise, polyuria, polydipsia	PP (Halop.)	N	N / NS	No patient history No family history	Y	5 days	Y	NS	Normal U(+) Stable	NS 1121 NS	NS 13.6 NS	Y	324 mmol/kg	Y	•IV insulin, rehydration (10 U, 3 times/ day)	Y, Metformin Glibenclamide	NS	Partial	N	•Valproic acid •Lithium	NS
6	Chen et al. (2011) [175]	•Thirst, fatigue, poor appetite depressed mood	PP (Arip./Sulp.)	N	N/ New Onset	Family history	After	5 months	Y (Quet).	D (24.7)	NS (U+) Normal.	67 815 NS	NS 13.6 6.5	Y	1084 mmol/kg	Y	•IV insulin, volume ressuscitation	Y, Glucose lowering agents	Y	Partial	NS	•Valproic acid •Citalopram	NS
7	Cheslock et al. (2022) [176][1]	•Generalized weakness and altered mental status, lethargy, polydipsia, and decreased appetite, dry mouth, afebrile,	MT	N	N / Y (NS)	No patient history No family history	After	2 weeks	Y	NS (29/OB)	NS U+ Improved	220 864.6 (st) NS	NS NS NS	N	375 mOsm/Kg	Y	IV insulin, aggressive IV fluids basal-bolus regimen of insulin	Y, insulin, oral hypoglycemic	Y	Partial	N	NS	NS

		tachycardia,<br>tachypnea , agitated and disoriented to person,<br>place, and time																					
8	Endoh et al. (2012) [177]	•Decreased consciousness Glasgow Coma Scale score: 10	MT	NS	N / N	Family History	Y	6 weeks	Y	NS (30.7/OB)	NS<br>U (4+)<br>NS	NS<br>1797.6 (st)<br>100	NS<br>12.3<br>5.4	Y	426 mOsm/kg	Y	•IV insulin and saline<br>•Subc. 70/30 (8 U/d)	N	Y	Full	•Depression	•Bromazepam<br>•Vamoxapine<br>•Lithium<br>•Mianserin<br>•Methylphenidate<br>•Paroxetine hydrochloride	N
9	Franco et al. (2012) [178]	•Weak, change in appetite, changes in mental status, episodes of unresponsiveness, twitching of left arm	MT	N	NS / NS	NS	N	NS	Y	NS	NS<br>NS<br>Normal.	NS<br>957<br>NS	NS<br>NS<br>NS	Y	428 mOsm/L	Y	•Insulin	NS	NS	Partial	•Hypertension<br>•Osteoporosis	•Simvastatin<br>•Memantine<br>•Donepezil<br>•Diltiazem<br>•Alendronate	N
10	Franco et al. (2015) [179]	•Fatigue, polyuria, polydipsia	MT	Y, zipras.	Before, T2DM	Patient history	NS	6 months	Y	NS (OB)	NS<br>NS<br>Controlled	NS<br>597 (st)<br>200	NS<br>10.4<br>NS	NS	NS	Y	•IV insulin (NS)	Y, Insulin	Y	Partial	•Hyperlipidemia<br>•Irritable bowel syndrome<br>•Generalised anxiety disorder	NS	N
11	Hanyu et al. 2022 [180]	•Nausea, akathisia, drowsiness, vomiting, decreased consciousness	PP (Asen.)	NS	Before, DM	Patient history	Before	one week	Y (Luras.)	NS	NS<br>NS<br>Improved	104<br>698<br>NS	NS<br>NS<br>NS	N	343 mOsm/Kg	Y	IV fluids and insulin	NS	NS	Partial	•Depression, ischemic gastritis	Lamotrigine	NS
12	Hui Fang et al. [94][2]	•Vomitting, abdominal pain, thirst, polyuria, polydipsia, tachycardic, tachypnoeic and drowsy with a Glasgow Coma Scale (GCS) of 10/15.	PP (Quet.)	N	N / Ketone prone DM, T2D	Family history	Y (temp)	2 weeks	Y (Risp./Quet.)	NS (33.2/OB)	NS<br>NS<br>Normalized	NS<br>826.6 (st)<br>100.8 (st)	NS<br>10.8<br>5.35	Y	402 mosm/L	Y	intravenous insulin, fluids and potassium	Y, insulin, metformin temp.	Y	Partial, (diet mgt)	Depression	•Sertraline<br>•Pregabalin<br>•Zolpidem	NS
13	Kaino et al. (2017) [181]	•Thirst, disturbed consciousness with fever, hyperglycemia, hyperosmolarity and elevated creatine phosphokinase. Glasgow Coma Scale: 11	MT	NS	N/ NS	No patient history	Y	4 months	Y	I	Normal<br>U (+)<br>Normal	94<br>762<br>NS	4.9<br>13.4<br>NS	Y	430 mOsm/L	Y	•IV insulin, rehydration, ventilator support.<br>•Oral therapy with vildagliptine	NS	NS	U	NS	NS	NS
14	Kaya et al. (2014) [182]	•Decreased oral intake, confusion, somnolence, tachycardia, dryness, reduced turgor<br>Glasgow coma scale: 14	MT	NS	Before, T2DM	Patient history	Y	25 days	Y	NS	Normal<br>(U+)<br>NS	NS<br>776<br>137	7.2 12<br>NS	N	400 mOsm/kg	Y	•IV insulin, hydration	Y, Metformin,gliclazide	NS	Partial	Dementia	•Oral antidiabetics	NS

15	Khan et al. (2011) [183]	•Tremor, agitation, coma	MT	N/A	NS / N/A	NS	NS	NS	N/A	NS (OB)	NS<br>NS<br>NS	NS<br>2,198<br>NS	NS<br>NS<br>NS	N	376 mOsmol/Kg	Y	•IV fluids, insulin, K, phosphate replacement	N/A	N/A	Fatal	•Childhood ADHD	NS	NS
16	Khanal et al. (2020) [184]	•Difficulty swallowing, weakness	MT	NS	Before, T2DM	NS	NS	2 weeks	Y	NS	NS	NS<br>1447 (st)<br>NS	5.1<br>10<br>7.4	NS	428 mOsm/kg	Y	Lantus and short-acting insulin	NS	N (lost to follow up)	Partial	•Dementia<br>•Depression<br>•Hypertension	•Hydrochlorothiazide<br>•Nifedipine,<br>•Spironolactone.<br>•Citalopram<br>•Donepezil<br>•Thiazide	NS
17	Létourneau et al. (2011) [185]	•Vomiting, dizziness, diminished level of consciousness, polyuria, polydipsia	MT	Y, halop.	N / T2DM	Family history	Y	10 weeks	Y	I (38/OB)	NS<br>NS<br>NS	132.6 (st)<br>1370.9 (st)<br>114.9 (st)	5.9<br>13.5<br>5.9	NS	NS	Y	•IV insulin hydration, (10U, tapered over 72 hours)	Y, Metformin	Y	Partial	NS	NS	N
18	Lim et al. (2025) [112][2]	•Nausea, vomiting	MT	Y, zipra.	NS / NS	NS	NS	2 years	Y	I	NS<br>U (+)<br>NS	NS<br>1720<br>NS	NS<br>13<br>NS	Y	349	Y	Fluids, Subcutaneous insulin glargine (Lantus) 20 units nocte and insulin aspart (NovoRapid) 5 units three times daily with meals.	NS	NS	NS	NS	NS	NS
19	Maust et al. (2015) [186]	•Fatigue, dizziness, increased urinary frequency	MT	NS	Before (on antidiabetic)	No family history	Y	1 week	Y	NS (27/OB)	Normal<br>NS<br>NS	NS<br>995<br>NS	5.6<br>9.7<br>6.1	Y	328 mOsm/Kg	Y	•Insulin, rehydration	Y, Sitagliptin, metformin	Y	Partial	NS	Metformin	N
20	McCall and Bourgeois (2004) [187]	•Unresponsiveness, polyuria, polydipsia, blurred vision, headache, nausea, vomiting	MT	N	N / NS	Family history	N	3 months	Y	NS (OB)	NS<br>(U+)<br>NS	NS<br>2123.5 (st)<br>118.7 (st)	NS<br>NS<br>NS	Y	399 mOsm/kg	Y	•IV insulin, fluids, antibiotics, (NPH insulin 29 U SQ qam and 17 U SQ qpm, and regular insulin 5 U SQ at each meal and use insulin sliding scale as needed.)	Y, Insulin	Y	Partial	•Seizure disorder<br>•hypertension	•Benztropine<br>•Valproic acid<br>•Hydrochlorothiazide	History of substance abuse
21	Meatherall et al. (2002) [188]	•Loss of appetite, abdominal discomfort, polydipsia, polyuria	MT	N/A	N / NA	No patient<br>No family history	N	3 weeks	N/A	I (28/OB)	NS<br>N/A<br>NS	130<br>284<br>NS	NS<br>12.3<br>N/A	Y	N/A	N/A	•N/A	N/A	N	Fatal	NS	N	History of alcohol and substance abuse

22	Milano et al. (2016) [189]	•Polydipsia, polyuria, malaise, vomiting diarrhea. Altered and agitated state, febrile, tachycardic, normotensive, Kussmaul respirations.	PP (Luras/Quet.)	NS	N / NS	No patient history	N	NS	NS	NS	NS<br>NS<br>NS	NS<br>1,403<br>N/A	NS<br>NS<br>NS	NS	371 mmol/kg	Y	•IV insulin, fluids	NS	Y	Fatal	N	NS	NS
23	Raza S. (2007) [190]	•Dehydration, abdominal pain, vomiting and drowsiness. Glasgow coma scale: 10	MT	NS	N / NS	No patient No family history	NS	1 week	Y	NS	NS<br>U(+)<br>Normal	104.8 (st)<br>2278.1 (st)<br>NS	NS<br>NS<br>NS	N	NS	Y	•IV insulin, saline, potassium replacement, prophylactic anticoagulant	NS	NS	Partial	N	NS	N
24	Rock et al. (2009) [191]	•Hyperthermia, decreased level of consciousness.<br>•Comatose on admission	MT	N/A	Before, T2DM	Patient history	Y	NS	N/A	SM	NS<br>NS<br>N/A	NS<br>880<br>N/A	NS<br>NS<br>N/A	N	380 mOsm/kg	Y	•IV insulin, saline (NS)	N/A	N/A	Fatal	NS	•Metformin<br>•Biperiden	NS
25	Roefaro and Mukherjee (2001) [192]	•Hallucinations, polydipsia, polyuria	MT	N	N / N	No patient history No family history	N	7.5 months	Y	SM (OB)	Normal<br>U (4)<br>NS	108<br>1596<br>105	5 13.3<br>NS	N	405 mOsm/L	Y	•IV Insulin, fluids (NPHS: 15 U morning; 10 U evening)	No	Y	Full	•Hypertension<br>•Coronary artery disease<br>•Esophageal strictures	•Gabapentin<br>•Isosorbide dinitrate<br>•Venlafaxine<br>•Lansoprazole	Ethanol abuse
26	Short and Nolan (1995) [193]	•Thirst, disorientation, fluctuating consciousness level, drowsiness, urinary incontinence, incoherence in speech	PP (Triflu/Chlorp.)	NS	N / NS	NS	Y	2 weeks	U	NS (OW)	Normal<br>NS<br>Normal	NS<br>1,541<br>NS	NS<br>NS<br>NS	N	NS	Y	•Actrapid insulin, IV saline, heparin	Y, Gibencalamide	NI NS	Partial	•Mild hypertension	N	N
27	Takanobu et al. (2015) [194]	•Obtundation, lethargy, afebrile, muscle pain, rigidity, restlessness, monology, high fever, stupor	PP (Levom/Chlorp.)	NS	Y / Mild DM prior	Patient history No family history	After	NS	NS	NS	NS<br>(U+)<br>Normal	118<br>449.1 (st)<br>NS	5.9 7<br>NS	N	402 mOsm/L	Y	•IV Insulin, fluids (Subc.)	N	Y	Partial (diet mgt.)	N	•Promethazine<br>•Phenobarbital<br>•Flunitrazepam<br>•Nitrazepam<br>•Biperiden<br>•Mianserin<br>•Antiuclerative	NS
28	Tollefson et al. (1983) [195]	•Polyuria, polydipsia. transient coma, delirium	MT	N	N / N	No patient No family history	N	9 days	Y	NS	Normal<br>NS<br>NS	87<br>1055.04 (st)<br>150	NS<br>NS<br>NS	N	314 mOsm/L	Y	•IV insulin	N	Y	Full	NS	Lithium	NS
29	Vakharia et al. 2022 [196]	fatigue, polyuria, and lethargy	MT	Y, arip.	N / Y, new onset	Family history	After	10 years	Y (Arip.)	D (17.9)	NS<br>NS<br>NS	NS<br>844<br>NS	NS<br>11.5<br>6.7	Y	398 mOsm/Kg	Y	IV fluid resuscitation, saline, IV insulin subcutaneous insulin regimen: insulin glargine (10units/day and insulin lispro, 3 times per day) with meals based on an insulin-to-carbohydrate ratio of 1:10	Y, insulin	Y	Partial	•Rett syndrome,<br>•Autism<br>•Spectrum disorder<br>•Dystonia,<br>•Seizure disorder<br>•Dysphagia/gastrostomy-tube dependence<br>•Neurocognitive deficits	•Oxcarbazepine,<br>•Clonazepam,<br>•Benztropine,<br>•Baclofen<br>•Dextroamphetamine<br>•Amphetamine<br>•Sertraline<br>•Polyethyleneglycol<br>•Levonorgestrel	NS
30	Yeung and Lee. (2015) [197]	•Fever and increased heart rate	MT	NS	NS/ NS	NS	N	NS	NS	NS	NS<br>NS<br>NS	NS<br>1568 (st)<br>NS	NS<br>NS<br>NS	NS	NS	Y	•IV Insulin, fluids	Y	NS	Partial	NS	•Methadone<br>•Trazodone	•Substance abuse

Abbreviations: BMI, body mass index; HbA1c, hemogolgin A1c; Pt, Patient; MT, monotherapy; OB, obesity; OW, overweight ; PP, polypharmacy; +, Positive; subc, subcutaneous; temp, temporarily.
Asen, Asenapine; Arip, Aripiprazole; Chlorp, Chlorpromazine; Halop, Haloperidol; Levom, Levomepromazine; Luras, Lurasidone; Quet, Quetiapine; Risp, Risperidone; Thior, Thioridazine; Triflu, Trifluoperazine; Zipra, Ziprasidone.
D, decrease; I, increase; N, no; NS, not specified; Y, yes.
[1]One HHS case was identified through supplemental searching outside the predefined search strategy.

[2]Two cases were diagnoses as demonstrating both DKA and HHS and are included [94,112]
[3]Reinstatment of the APD is not considered a discontinuation for the purpose of this analysis.
[4]Standardized blood glucose values are shown (st).
[5] Complications occurring at or around the same time as DKA are not discussed (e.g., Neuroleptic Malignant Syndrome, pancreatitis).

Table S4. Critical appraisal of case reports (n = 151) against eight criteria detailed in the Joanna Briggs Tool[1,2].

	Criteria								Overall Assessment
Citation	(1) Were the patient's demographic characteristics clearly described?	(2) Was the patient's history clearly described and presented as a timeline?	(3) Was the current clinical condition of the patient on presentation clearly described?	(4) Were diagnostic tests or assessment methods and the results clearly described?	(5) Was the intervention(s) or treatment procedure(s) clearly described	(6) Was the post-intervention clinical condition clearly described?	(7) Were adverse events (harms) or unanticipated events identified and described?	(8) Does case report provide takeaway lessons?	Does it meet all 8 criteria
Adhoni et al. (2021)	Y	N	Y	Y	y	y	y	y	N
Agrawal et al. (2016)	Y	N	Y	Y	Y	Y	N	Y	N
Ahuja et al. (2010)	Y	Y	Y	Y	N	Y	N	Y	N
Ai et al. (1998)	Y	N	Y	Y	N	Y	N	Y	N
Akunjee et al. (2018)	N	N	N	N	N	Y	N	Y	N
Al-Amri (2009) [45]	Y	Y	Y	Y	Y	Y	N	Y	N
Alex et al. (2018) [46]	Y	Y	Y	Y	N	Y	N	Y	N
Almahmood et al.	N	Y	Y	Y	N	Y	N	Y	N
Ananth et al. (2004)	Y	Y	Y	Y	N	Y	N	N	N
Aruna et al. (1995)	Y	Y	Y	Y	Y	Y	Y	Y	Y
Atabay and Arman (2019)	N	Y	Y	N	N	N	N	Y	N
Avella et al. (2004) (Case 1)	Y	Y	N/A	N/A	N/A	N/A	N/A	N	N
Avella et al. (2004) (Case 2)	Y	N	N/A	N/A	N/A	N/A	N/A	N	N
Avella et al. (2004) (Case 3)	Y	N	N/A	N/A	N/A	N/A	N/A	N	N

Avram (2004)	Y	Y	Y	Y	Y	Y	N	Y	N
Bae et al. (2024)	N	N	Y	Y	N	Y	N	Y	N
Balzan & Cacciottolo (1992)	Y	Y	Y	Y	Y	Y	N	Y	N
Buch et al. (2003)	Y	Y	Y	Y	Y	Y	N	Y	N
Campanella et al. (2009)	Y	N	Y	Y	Y	Y	N	N	N
Carbera et al. 2021	N	N	Y	Y	N	N	N	N	N
Cardinale et al. (2019)	Y	N	N	Y	Y	N	N	N	N
Cerimele (2008) [137]	Y	Y	Y	Y	N	N	N	Y	N
Chellamuthu et al.(2010)	Y	N	Y	Y	Y	Y	N	N	N
Chen et al. (2003)	Y	Y	Y	Y	N	Y	N	Y	N
Chen et al. (2011)	Y	Y	Y	Y	N	Y	N	Y	N
Cheslock et al. 2022	N	Y	Y	Y	Y	Y	N	Y	N
Cho & Lindenmayer (2009)	Y	Y	Y	N	N	Y	N	Y	N
Church et al. (2005)	Y	N	Y	Y	Y	Y	N	Y	N
Colli et al. (1999)	Y	Y	Y	Y	Y	Y	N	Y	N
Courvoisie et al. (2004)	Y	Y	Y	Y	Y	Y	Y	Y	Y
Croarkin et al. (2000)	Y	N	Y	Y	N	N	N	N	N
Crown et al. (2007)	N	Y	Y	Y	Y	Y	N	Y	N
Dahri et al. (2002)	Y	Y	Y	Y	N	N	N	Y	N
Das et al. (2018)	N	Y	Y	Y	Y	N	N	Y	N

de Boer & Gaete (1992)	N	Y	Y	Y	N	N	N/A	N	N
Dhamija et al. (2008)	Y	N	N	Y	N	N	N	Y	N
Dibben et al. 2005) (Case 1)	Y	Y	Y	Y	Y	Y	N	Y	N
Dibben et al. (2005) (Case2)	Y	Y	Y	Y	N	Y	N	Y	N
Doodnauth et al. (2021)	Y	N	Y	Y	N	Y	N	Y	N
Endoh et al. (2012)	Y	Y	Y	Y	Y	Y	N	Y	N
Franco et al. (2012)	Y	N	Y	N	N	N	N	Y	N
Franco et al. (2015)	Y	N	Y	Y	N	Y	N	Y	N
Fulbright and Breedlove (2006)	Y	Y	Y	Y	N	Y	N	Y	N
Ghandi and Ghanesh (2019)	N	Y	Y	N	N	N	N	Y	N
Gatta et al. (1999)	Y	Y	Y	Y	N	Y	N	Y	N
Goldstein et al. (1999) (Case 1)	Y	Y	Y	Y	N	Y	N	Y	N
Goldstein et al. (1999) (Case 2)	Y	Y	Y	Y	N	Y	N	Y	N
Greenfield et al. (2002)	N	N	N	Y	N	N	N	N	N
Hanyu et al. (2022)	N	Y	Y	Y	N	N	N	Y	N
Hepburn and Brzozowska (2016)	Y	Y	Y	Y	Y	Y	N	Y	N
Hörber et al. (2018)	N	Y	Y	Y	N	Y	N	Y	N

Howes & Rifkin (2004)	N	Y	Y	Y	N	Y	N	Y	N
Hui Fang et al. (2018)	Y	Y	Y	Y	Y	Y	N	N	N
Hussain et al. (2024)	N	N	Y	Y	N	N	N	Y	N
Itoh et al. (2019)	Y	Y	Y	Y	Y	Y	Y	Y	Y
Jain et al. 2024	Y	Y	Y	Y	Y	Y	Y	Y	Y
Iwaku et al. (2017)	N	Y	Y	Y	Y	Y	N	N	N
Jalota et al. 2015)	N	Y	Y	Y	N	N	Y	Y	N
Johnson et al. (2002	Y	Y	Y	Y	N	Y	N	N	N
Juneja et al. (2021)	N	N	Y	Y	Y	Y	N	Y	N
Kahn & Bourgeois (2007)	Y	Y	Y	Y	N	Y	N	Y	N
Kaino et al. (2017)	Y	Y	Y	Y	N	Y	N	N	N
Kanagaretnam et al. (2022)	N	N	Y	Y	N	Y	N	Y	N
Kasmi (2013)	N	N	N	N	N	N	N	N	N
Kaya et al. (2014)	Y	Y	Y	Y	N	N	N	Y	N
Khan et al. (2011)	N	N	Y	Y	N	N/A	N/A	Y	N
Khanal et al. (2020)	Y	Y	Y	Y	N	N	N	Y	N
Kibbey et al. (2010)	Y	Y	Y	Y	N	Y	N	Y	N
Kinoshita et al. (2014)	Y	Y	Y	Y	Y	Y	N	Y	N
Kostakoğlu et al. (1996)	Y	Y	Y	Y	Y	Y	Y	Y	Y

Koval et al. (1994)	Y	N	Y	Y	Y	Y	Y	N	N
Kyriazis et al. (2006)	Y	Y	Y	Y	Y	Y	N	Y	N
Lafayette et al. (2003)	Y	N	Y	Y	Y	Y	N	Y	N
Laghate & Gupta (2004)	N	N	Y	Y	N	Y	N	Y	N
Létourneau et al. (2011)	Y	Y	Y	Y	Y	Y	N	Y	N
Lim et al. (2025)	N	Y	Y	Y	N	N	N	Y	N
Lindenmayer & Patel. (1999)	Y	Y	Y	Y	Y	Y	N	Y	N
Lu & Yan (2009)	N	N	N	Y	N	N/A	N/A	Y	N
Macfarlane & Fisher (2006)	Y	Y	Y	Y	N	Y	N	N	N
Madsen (2014)	Y	Y	Y	N	N/A	N/A	Y	E	N
Mahmoud et al. (2023)	N	N	Y	Y	N	Y	N	Y	N
Makhzoumi et al. (2008)	Y	Y	Y	Y	N	Y	N	Y	N
Maksimoviæ and Pavliæ-Renar (2006)	Y	N	Y	Y	N	Y	N/A	N	N
Marlowe et al. (2007)	N	N	N	Y	N	Y	N	Y	N
Maule et al. (1999)	Y	Y	Y	Y	N	Y	Y	Y	N
Maust et al. (2015)	Y	N	Y	Y	N	Y	N	Y	N
McCalmon and Weide (2021)	Y	Y	N	Y	Y	Y	N	Y	N

McCall & Bourgeois (2004)	N	N	Y	Y	Y	Y	N	Y	N
Meatherall et al. (2002)	Y	Y	Y	Y	N/A	N/A	N/A	N	N
Milano et al. (2016)	N	N	Y	Y	N	N	N	Y	N
Miller et al. (2008)	Y	N	Y	Y	N	N	N	Y	N
Mithat et al. (2005)	N	Y	N	Y	Y	Y	N	N	N
Mohan (1999)	Y	Y	Y	Y	N	Y	N	N	N
Muench & Carrey (2001)	Y	N	Y	Y	Y	Y	N	Y	N
Murakami et al. 2025	N	N	Y	Y	Y	Y	N	Y	N
Nagamine (2021)	Y	Y	Y	Y	N	Y	N	Y	N
Nahas et al. (2010)	N	Y	Y	N	N	N	N	Y	N
Nakanishi (2025)	N	Y	N	Y	Y	Y	N	Y	N
Ng and Broussard (2024)	N	N	Y	Y	N	N	N	Y	N
Niazy et al. (2007)	Y	Y	Y	Y	N	Y	Y	Y	N
Nicolai (2001)	Y	Y	Y	Y	N	Y	N	N	N
Ogunnaya et al. 2024	N	N	Y	Y	N	Y	N	N	N
Patel et al. (2011)	N	N	N	Y	Y	N	N	Y	N
Pathmanathan (2013)	N	N	Y	Y	Y	Y	N	Y	N
Peterson & Bird (1996)	Y	Y	Y	Y	N	Y	N	N	N
Pierides (1997)	Y	Y	Y	Y	N	N	N	N	N
Pillai et al. (2006)	N	Y	Y	Y	N	Y	N	Y	N

Popli et al. (1997)	Y	N	Y	Y	N	Y	N	Y	N
Ragucci & Wells (2001)	Y	Y	Y	Y	Y	Y	N	Y	N
Rahat et al. (2005)	Y	N	Y	Y	N	N	N	N	N
Rashid et al. (2009)	Y	Y	Y	Y	N	N	N	Y	N
Ratnakaran (2015)	N	Y	Y	Y	Y	Y	N	Y	N
Raza S. (2007)	N	N	Y	Y	N	Y	N	Y	N
Reddymasu et al. (2006)	N	Y	Y	Y	N	N	N	Y	N
Rock et al. (2009)	N	N	Y	Y	N	N/A	N/A	Y	N
Roefero & Mukherjee(2001)	Y	Y	Y	Y	Y	Y	N	Y	N
Sa et al. (2013)	Y	Y	Y	Y	N	Y	N	Y	N
Sato et al. (2008)	N	Y	Y	Y	N	Y	N	N	N
Seaburg et al. (2001)	Y	Y	Y	Y	Y	Y	Y	Y	N
Selva & Scott (2001)	Y	Y	Y	Y	Y	N	N	Y	Y
Shin et al. 2025	N	Y	Y	Y	Y	Y	Y	Y	N
Short & Nolan (1994)	Y	Y	Y	Y	Y	Y	N	N	N
Singh et al. (2013)	N	Y	Y	Y	N	Y	Y	N	N
Singh et al. (2020)	N	Y	Y	Y	N	N	N	Y	N
Sirois (2008)	Y	Y	Y	Y	N	N	N	N	N
Smith et al. (1999)	N	Y	Y	Y	N	N	N	Y	N

Strassnig et al. (2013) (Case 1)	N	Y	N	Y	N	Y	N	Y	N
Straker et al. (2002)	Y	Y	Y	Y	N	N	Y	Y	N
Takahashi et al. (2005)	Y	Y	Y	Y	Y	Y	N	Y	N
Takanobu et al. (2015)	N	Y	Y	Y	N	Y	N	Y	N
Tavakoli & Arguisola (2003)	Y	Y	N	N	N	N	N	Y	N
Thanikonda et al. (2020)	Y	Y	Y	N	N	N	N	Y	N
Tollefson et al. (1983)	Y	Y	Y	Y	N	Y	N	N	N
Torrey et al. (2003)	Y	Y	N	N	N	N/A	N/A	Y	N
Taslipinar et al. (2008)	N	Y	Y	Y	N	Y	N	Y	N
Tsuchiyama et al. (2004)	Y	Y	Y	Y	Y	Y	N	Y	N
Tugwell et al. (2020)	Y	N	Y	Y	Y	Y	Y	Y	N
Vakharia et al. 2022	Y	Y	Y	Y	Y	Y	N	Y	N
Varma et al. (2007)	N	U	Y	Y	N	N	N	Y	N
Vincent et al. (2017)	N	Y	Y	Y	N	Y	N	Y	N
Vuk et al. (2017)	Y	Y	Y	Y	N	Y	N	N	N
Waldman & Yaren (2002)	Y	Y	Y	Y	N	Y	N	Y	N
Watkins et al. (2011)	Y	Y	Y	Y	Y	Y	N	Y	N
Whicher et al. (2019)	Y	N	Y	N	Y	Y	Y	Y	N
Wilson et al. (2002) (Case 1)	Y	N	Y	Y	Y	N	N	N	N

Wilson et al. (2002) (Case 2)	Y	N	Y	Y	Y	N	N	N	N
Wilson et al. (2002) (Case 3)	N	Y	N	N	N	N	N	N	N
Wilson et al. (2002) (Case 4)	N	Y	Y	N	Y	N	N	N	N
Wilson et al. (2002) (Case 5)	N	Y	N	Y	N	Y	N	N	N
Wong et al. (2007)	Y	Y	Y	Y	Y	Y	N	Y	N
Yeung & Lee. (2015)	N	N	Y	Y	N	N	N	N	N

[1]The Joanna Briggs Tool for Critical Appraisal was used as a quality assessment tool for each case report [55,56].
[2]Cases where fatalities occurred could not be assessed for every criterion.
